# Safety of Simultaneous vs Sequential mRNA COVID-19 and Inactivated Influenza Vaccines: A Randomized Clinical Trial

**DOI:** 10.1001/jamanetworkopen.2024.43166

**Published:** 2024-11-06

**Authors:** Emmanuel B. Walter, Elizabeth P. Schlaudecker, Kawsar R. Talaat, Wes Rountree, Karen R. Broder, Jonathan Duffy, Lisa A. Grohskopf, Marek S. Poniewierski, Rachel L. Spreng, Mary A. Staat, Rediet Tekalign, Oidda Museru, Anju Goel, Grace N. Davis, Kenneth E. Schmader

**Affiliations:** 1 Department of Pediatrics, Duke University School of Medicine, Durham, North Carolina; 2 Duke Human Vaccine Institute, Durham, North Carolina; 3 Division of Infectious Diseases, Department of Pediatrics, University of Cincinnati College of Medicine, Cincinnati Children’s Hospital Medical Center, Cincinnati, Ohio; 4 Center for Immunization Research, Department of International Health, The Johns Hopkins Bloomberg School of Public Health, Baltimore, Maryland; 5 Immunization Safety Office, National Center for Emerging and Zoonotic Infectious Diseases, Centers for Disease Control and Prevention, Atlanta, Georgia; 6 Influenza Division, Centers for Disease Control and Prevention, Atlanta, Georgia; 7 Loyal Source Government Services, Centers for Disease Control and Prevention, Orlando, Florida; 8 Department of Medicine, Duke University School of Medicine, Durham, North Carolina

## Abstract

**Importance:**

Limited randomized clinical trial data exist on the safety of simultaneous administration of COVID-19 and influenza vaccines.

**Objective:**

To compare the reactogenicity, safety, and changes in health-related quality of life (HRQOL) after simultaneous vs sequential receipt of messenger RNA (mRNA) COVID-19 vaccine and quadrivalent inactivated influenza vaccine (IIV4).

**Design, Setting, and Participants:**

This randomized, placebo-controlled clinical trial was conducted between October 8, 2021, and June 14, 2023, at 3 US sites. Participants were nonpregnant persons aged 5 years or older with the intention of receiving both influenza and mRNA COVID-19 vaccines.

**Interventions:**

Intramuscular administration in opposite arms of either IIV4 or saline placebo simultaneously with mRNA COVID-19 vaccine at visit 1. Those who received placebo at visit 1 received IIV4 and those who received IIV4 at visit 1 received placebo 1 to 2 weeks later at visit 2.

**Main Outcomes and Measures:**

The primary composite reactogenicity outcome was the proportion of participants with fever, chills, myalgia, and/or arthralgia of moderate or greater severity within 7 days after vaccination visits 1 and/or 2, using a 10% noninferiority margin. Secondary outcomes were solicited reactogenicity events and unsolicited adverse events (AEs) for 7 days after each visit separately and HRQOL after visit 1, assessed by the EuroQol 5-Dimension 5-Level (EQ-5D-5L) Index. Serious AEs (SAEs) and AEs of special interest (AESIs) were assessed for 121 days. Outcomes were compared between groups.

**Results:**

A total of 335 persons (mean [SD] age, 33.4 [15.1] years) were randomized (169 to the simultaneous group and 166 to the sequential group); 211 (63.0%) were female, and 255 (76.1%) received bivalent BNT162b2 mRNA COVID-19 vaccine. The proportion with the primary composite reactogenicity outcome in the simultaneous group (25.6% [n = 43]) was noninferior to the proportion in the sequential group (31.3% [n = 52]) (site-adjusted difference, –5.6 percentage points [pp]; 95% CI, −15.2 to 4.0 pp). Respective proportions in each group were similar after each visit separately (visit 1, 40 [23.8%] vs 47 [28.3%]; visit 2, 5 [3.0%] vs 9 [5.4%]). No significant group differences in participants with AEs (21 [12.4%] vs 16 [9.6%]), SAEs (1 [0.6%] vs 1 [0.6%]), and AESIs (19 [11.2%] vs 9 [5.4%]) were observed in the simultaneous vs sequential groups, respectively. Among participants with severe reactogenicity, the mean (SD) EQ-5D-5L Index score decreased from 0.92 (0.08) to 0.92 (0.09) prevaccination to 0.81 (0.09) to 0.82 (0.12) postvaccination.

**Conclusions and Relevance:**

In this randomized clinical trial assessing simultaneous vs sequential administration of mRNA COVID-19 and IIV4 vaccines, reactogenicity was comparable in both groups. These findings support the option of simultaneous administration of these vaccines.

**Trial Registration:**

ClinicalTrials.gov Identifier: NCT05028361

## Key Points

**Question:**

What are the comparative reactogenicity, safety, and short-term effects on health-related quality of life of simultaneous vs sequential receipt of messenger RNA (mRNA) COVID-19 and influenza vaccines?

**Findings:**

In this randomized, placebo-controlled clinical trial of 335 persons receiving mRNA COVID-19 and inactivated influenza vaccines simultaneously vs sequentially 1 to 2 weeks apart, the proportion of participants with at least 1 moderate or severe reaction of fever, chills, myalgia, or arthralgia was not higher after simultaneous compared with sequential receipt.

**Meaning:**

The findings support simultaneous administration of mRNA COVID-19 and inactivated influenza vaccines as an acceptable option to achieve timely vaccination.

## Teaser

This randomized clinical trial compares reactogenicity, safety, and short-term health-related quality-of-life outcomes after simultaneous vs sequential receipt of messenger RNA (mRNA) COVID-19 and influenza vaccines.

## Introduction

In the challenge against seasonal respiratory illnesses, influenza and COVID-19 vaccines have been associated with decreased hospitalizations and deaths resulting from infection.^1,2,3,4,5,6^ Both vaccines are recommended for use in persons aged 6 months or older in the US.^7,8,9^ Since both influenza and SARS-CoV-2 viruses tend to cause winter peaks of disease, prevention efforts target administering both vaccines during the late summer and early fall.^10^ To facilitate the convenient delivery of these vaccines to the public, the Centers for Disease Control and Prevention (CDC) Advisory Committee on Immunization Practices (ACIP) guidance allows for their simultaneous administration at different anatomic sites.^11,12,13^

At the time the current trial was designed and implemented, limited data existed describing the safety of simultaneous administration of influenza and messenger RNA (mRNA) COVID-19 vaccines. This trial was conducted to provide additional evidence on the safety of the current recommendation for simultaneous influenza and mRNA COVID-19 vaccination.

## Methods

### Study Design and Participants

We conducted a prospective, randomized, placebo-controlled clinical trial between October 8, 2021, and June 14, 2023, at 3 CDC-sponsored Clinical Immunization Safety Assessment (CISA) Project^14^ sites during the October 8, 2021, to July 28, 2022, and September 16, 2022, to June 14, 2023, influenza seasons: Cincinnati Children’s Hospital Medical Center, Duke University, and The Johns Hopkins University. The trial protocol (Supplement 1), which included a statistical analysis plan, was approved by the institutional review board (IRB) at each site; the CDC executed a reliance agreement with the Duke University Health System IRB. Written informed consent was obtained from all participants or their legal guardians. This report is in accordance with the Consolidated Standards of Reporting Trials (CONSORT) reporting guideline for randomized studies.

Eligibility criteria included intention of receiving both an influenza and mRNA COVID-19 vaccine as recommended by the CDC and ACIP and age of 5 years or older if receiving a primary 2-dose mRNA COVID-19 vaccine series or age of 12 years or older if receiving a booster mRNA COVID-19 vaccine. Persons who by self-report were currently pregnant, planning to become pregnant within 3 months, or likely to become pregnant per screening criteria were excluded. Persons with a contraindication to either vaccine or with immunosuppression were also excluded. A complete listing of eligibility criteria is included in the eMethods in Supplement 2.

After obtaining written informed consent, study staff reviewed participant eligibility and collected demographics, medical history, SARS-CoV-2 infection history, and COVID-19 and influenza vaccination status. Race and ethnicity, ascertained by self-report, were included to describe the diversity of the study population. Race categories were Asian, Black or African American, White, multiracial, and unknown or not reported, and ethnicity categories were Hispanic, non-Hispanic, and unknown or not reported. A prevaccination blood sample was obtained to determine baseline SARS-CoV-2 serostatus. All participants received an open-label mRNA COVID-19 vaccine that the US Food and Drug Administration (FDA) had approved or authorized for emergency use (BNT162b2 or mRNA-1273 [original monovalent] during the 2021-2022 season and BNT162b2 or mRNA-1273 [bivalent], both containing original and Omicron BA.4/BA.5, during the 2022-2023 season^15,16,17,18^). In accordance with the evolving FDA authorizations and approvals and CDC recommendations,^11,19^ the vaccines were used as a primary series or booster.^15,16,17,18^

The mRNA COVID-19 vaccine was administered intramuscularly at day 1 (visit 1) either as standard of care or as a study procedure. Participants were randomized (1:1) to receive either the respective season’s FDA-approved, egg-based quadrivalent inactivated influenza vaccine (IIV4) (simultaneous group) or saline placebo (sequential group) on day 1 in the arm opposite of where the mRNA COVID-19 vaccine was administered. Persons aged 65 years or older received influenza vaccine high-dose quadrivalent (0.7 mL)^20^; those younger than 65 years received standard-dose influenza vaccine or quadrivalent (0.5 mL).^20,21^ At day 8 to 15 (visit 2), those administered IIV4 at visit 1 received placebo, and those administered placebo at visit 1 received IIV4. A comparable volume of saline placebo was administered as an injection according to participant age. We used a permuted block randomization scheme stratified by age (5-11 years, 12-17 years, 18-64 years, and ≥65 years), receipt of primary series or booster dose, and study site. Those receiving an mRNA COVID-19 vaccine primary series received a second dose per the CDC recommended schedule. Vaccine and placebo were administered by unblinded study staff who did not participate in outcome measurements. Participants and remaining study staff were blinded to treatment allocation.

### Safety and Reactogenicity Assessments

Solicited reactions and unsolicited adverse events (AEs) were monitored on day 1 (vaccination day) through day 7 using a memory aid. Participants chose either to submit memory aid information daily electronically or to bring it to a subsequent clinic visit. Solicited reactions assessed included local injection site (pain, induration and/or swelling, erythema [redness], and axillary swelling and/or tenderness) and systemic (fever, chills, myalgia, arthralgia, headache, fatigue, nausea and/or vomiting, and diarrhea) reactions. Participants received a thermometer, ruler, and education about memory aid completion. The severity of each solicited reaction was graded based on criteria used by mRNA COVID-19 vaccine trials^22,23^ (eMethods in Supplement 2). Serious adverse events (SAEs) and adverse events of special interest (AESIs) and corresponding medication changes were assessed through day 121. SAEs were defined in accordance with the FDA.^24^ AESIs were COVID-19 illness, multisystem inflammatory syndrome, Guillain-Barré syndrome, allergic-type reactions (anaphylaxis, hives, and/or facial and limb swelling occurring within 7 days of vaccination), and myocarditis and/or pericarditis. Trial investigators assessed AE severity and relatedness to receipt of vaccines or placebo.

### Health-Related Quality-of-Life Assessment

For persons aged 12 years or older, health-related quality of life (HRQOL) was assessed prevaccination on day 1 (in clinic) and then daily for 7 days using the EuroQol 5-Dimension 5-Level (EQ-5D-5L) Index and EuroQol 5-Dimension 5-Level Visual Analogue Scale (EQ-VAS). The EQ-5D-5L is a standardized, generic measure of health status (score range, −0.109 to 1, with higher scores indicating better quality of life). The EQ-5D-5L provides information on HRQOL and activities of daily living relevant to adolescents and adults: mobility, self-care, usual activities, pain or discomfort, and anxiety and/or depression.^25,26^ The EQ-VAS (score range, 0 to 100, with higher scores indicating better quality of life) measures the respondent’s self-rated health. Permission from EuroQol was obtained prior to using the HRQOL surveys.

### Baseline COVID-19 Serostatus

Prevaccination serum samples were assessed by chemiluminescent microparticle immunoassays for the presence of preexisting SARS-CoV-2 nucleocapsid immunoglobulin G (IgG) antibodies using the Alinity I SARS-CoV-2 IgG assay (Abbott Laboratories).^27^ Results were interpreted as positive or negative.

### Outcome Measures

The primary study outcome was a composite reactogenicity outcome, defined as the occurrence of fever, chills, myalgia, and/or arthralgia graded as moderate (grade 2), severe (grade 3), or potentially life threatening (grade 4) during days 1 to 7 after visits 1 and 2 combined. These solicited reactions were considered to be clinically meaningful and were solicited in both BNT162b2 and mRNA-1273 mRNA COVID-19 vaccine prelicensure trials. We hypothesized that the proportion of participants with the primary composite reactogenicity outcome would be noninferior in the simultaneous group vs the sequential group. Secondary analyses included the proportion of participants with the composite reactogenicity outcome during days 1 to 7 after each vaccination visit separately and the mean maximal change in HRQOL scores from baseline within days 1 to 7 after visit 1. Other outcomes described the proportions of participants with solicited local and systemic reactions of any severity and by severity grade and unsolicited AEs on days 1 to 7 and the proportion with AESIs and SAEs on days 1 to 121 after vaccination visits. An additional exploratory post hoc analysis was conducted assessing the composite reactogenicity outcome after visits 1 and 2 combined and after each visit separately stratified by sex with no adjustment made to the α level.

For all outcomes, the comparison assessed was between the simultaneous and sequential groups. The AE and SAE comparisons were analyzed using the modified intention-to-treat (MITT) population 2, which consisted of all participants randomized and vaccinated. Reactogenicity outcomes were analyzed using the MITT population 1, which consisted of all participants who were randomized, received at least 1 study vaccine at visit 1, and provided at least 1 day of complete data on the symptom diary.

### Statistical Analysis

The planned sample size of at least 428 evaluable participants (214 per group) provided at least 80% power to reject the null hypothesis that the proportion of participants with the primary composite reactogenicity outcome in the simultaneous group would be inferior to the sequential group. Anticipating dropout, the study aimed to enroll at least 450 participants.

The statistical testing for the primary composite reactogenicity outcome was conducted at the 1-sided α = .025 level using the upper bound of a stratified-by-site Newcombe binomial CI^28^ with Cochran-Mantel-Haenszel weighting and a noninferiority margin of 10%. Additional comparisons of proportions between the treatment groups used an exact Mantel-Haenszel statistic in a stratified analysis by site to control for the randomization blocks at the 2-sided α = .05 level. Study site adjusted odds ratios (AORs) and corresponding 95% CIs for proportions were also calculated. The changes in HRQOL after visit 1 were evaluated using the Mann-Whitney *U* test. For the HRQOL comparisons, we used a 2-sided α = .05 level. Summary statistics were used to describe reactogenicity and AE outcomes, and 95% CIs of the difference between vaccination groups were calculated. The data were analyzed using SAS/STAT software, version 9.4 (SAS Institute Inc). One-sided *P* < .025 was significant.

## Results

### Study Population

**Figure 1.**
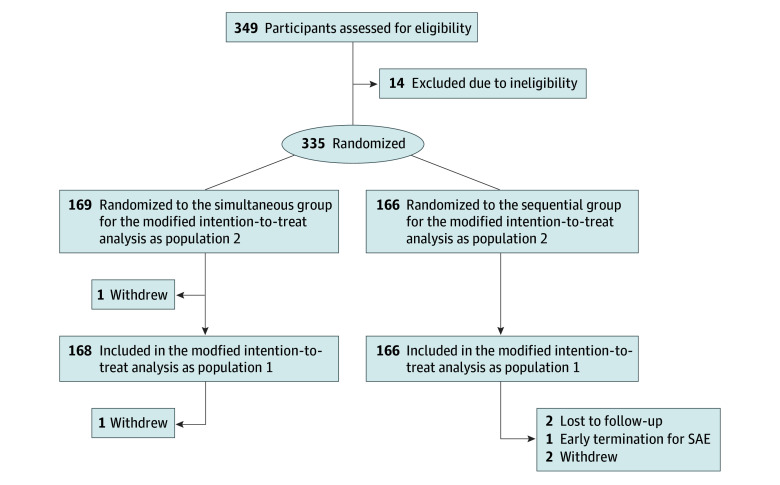
Trial Flow Diagram SAE indicates serious adverse effect.

We assessed 349 persons for eligibility, of whom a total of 335 (mean (SD) age of 33.4 [15.1] years) were randomized to either the simultaneous group (n = 169) or the sequential group (n = 166) (Figure 1). One individual in the simultaneous group (0.6%) voluntarily withdrew after randomization. Most of the study population was female (211 [63.0%] compared with 124 [37.0%] male), with more female participants randomized to the sequential than the simultaneous group (Table). Twenty-seven participants (8.1%) were Asian; 60 (17.9%), Black or African American; 229 (68.4%), White; 16 (4.8%), multiracial; and 3 (0.9%), of unknown or not reported race. Twenty-one participants (6.3%) were of Hispanic ethnicity, 312 (93.1%) of non-Hispanic ethnicity, and 2 (0.6%) of unknown or not reported ethnicity. Most participants were aged 18 to 64 years (289 [86.3%]) and were enrolled in the 2022-2023 study season (262 [78.2%]). All but 4 participants (1.2%) completed a COVID-19 vaccine primary series prior to entering the study, and all but 11 (3.3%) received the BNT162b2 mRNA COVID-19 vaccine during the study; 255 (76.1%) received bivalent BNT162b2 mRNA COVID-19 vaccine. Over half of participants (191 [57.0%]) reported a history of SARS-CoV-2 infection or had detectable nucleocapsid IgG antibody at enrollment. Enrollment did not reach the targeted sample size due to logistical issues that prevented 1 site from having a population of people receiving mRNA COVID-19 vaccine from which to recruit.

**Table.** Participant Characteristics at Enrollment Across 3 CISA Project Sites During the 2021-2022 and 2022-2023 Influenza Seasons

Characteristic	Participants, No. (%)		
	Simultaneous group (n = 169)^a^	Sequential group (n = 166)^b^	Total (N = 335)
Sex			
Female	96 (56.8)	115 (69.3)	211 (63.0)
Male	73 (43.2)	51 (30.7)	124 (37.0)
Race			
Asian	12 (7.1)	15 (9.0)	27 (8.1)
Black or African American	30 (17.8)	30 (18.1)	60 (17.9)
White	117 (69.2)	112 (67.5)	229 (68.4)
Multiracial	9 (5.3)	7 (4.2)	16 (4.8)
Unknown or not reported	1 (0.6)	2 (1.2)	3 (0.9)
Ethnicity			
Hispanic	12 (7.1)	9 (5.4)	21 (6.3)
Non-Hispanic	156 (92.3)	156 (94.0)	312 (93.1)
Unknown or not reported	1 (0.6)	1 (0.6)	2 (0.6)
Age, y			
5-11	4 (2.4)	2 (1.2)	6 (1.8)
12-17	11 (6.5)	13 (7.8)	24 (7.2)
18-64	146 (86.4)	143 (86.1)	289 (86.3)
≥65	8 (4.7)	8 (4.8)	16 (4.8)
Mean (SD)	33.7 (15.3)	33.1 (14.9)	33.4 (15.1)
CISA study site			
CCHMC	67 (39.6)	63 (38.0)	130 (38.8)
Duke University	79 (46.7)	81 (48.8)	160 (47.8)
JHU	23 (13.6)	22 (13.3)	45 (13.4)
Influenza season			
2021-2022	37 (21.9)	36 (21.7)	73 (21.8)
2022-2023	132 (78.1)	130 (78.3)	262 (78.2)
mRNA COVID-19 vaccine brand			
BNT162b2 monovalent (original)	34 (20.1)	35 (21.1)	69 (20.6)
BNT162b2 bivalent	130 (76.9)	125 (75.3)	255 (76.1)
mRNA-1273 monovalent (original)	4 (2.4)	4 (2.4)	8 (2.4)
mRNA-1273 bivalent	1 (0.6)	2 (1.2)	3 (0.9)
Prior COVID-19 vaccine	165 (97.6)	166 (100)	331 (98.8)
Prior COVID-19 vaccine brand			
BNT162b2	116 (68.6)	125 (75.3)	241 (71.9)
mRNA-1273	49 (29.0)	41 (24.7)	90 (26.9)
Unknown	4 (2.4)	0	4 (1.2)
Prior influenza vaccine	139 (82.2)	139 (83.7)	278 (83.0)
Prior COVID-19 diagnosis	76 (45.0)	82 (49.4)	158 (47.2)
Prior positive COVID-19 test	74 (43.8)	80 (48.2)	154 (46.0)
Positive nucleocapsid IgG antibody at baseline	51 (30.2)	49 (29.5)	100 (29.8)
Prior COVID-19 diagnosis or positive nucleocapsid IgG antibody at baseline	96 (56.8)	95 (57.2)	191 (57.0)

Abbreviations: CCHMC, Cincinnati Children’s Hospital Medical Center; CISA, Clinical Immunization Safety Assessment; IgG, immunoglobulin G; JHU, The Johns Hopkins University; mRNA, messenger RNA.

^a^ Simultaneous vaccination with mRNA COVID-19 and inactivated influenza vaccines.

^b^ Sequential vaccination with mRNA COVID-19 and inactivated influenza vaccines.

### Solicited Reactogenicity

The proportion (percentage) of participants experiencing the primary composite reactogenicity outcome in the 7-day period after visit 1 and/or visit 2 was noninferior, but not statistically superior, when comparing the simultaneous group (43 participants [25.6%]) with the sequential group (52 participants [31.3%]) (site-adjusted difference, −5.6 percentage points [pp]; 95% CI, −15.2 to 4.0 pp). Similar percentages of participants in each group had the composite reactogenicity outcome during the 7-day period after visit 1 (40 [23.8%] and 47 [28.3%], respectively; AOR, 0.80 [95% CI, 0.49-1.30]; *P* = .39). Fewer but similar percentages of participants in each group reported the composite reactogenicity outcome in the 7-day period after visit 2 (simultaneous, 5 [3.0%]; sequential, 9 [5.4%]; AOR, 0.54 [95% CI, 0.18-1.63]; *P* = .29). In an exploratory post hoc analysis, noninferiority was still met, and the proportions of participants reporting these composite reactogenicity outcomes after visits 1 and 2 separately did not differ when assessing each sex alone.

**Figure 2.**
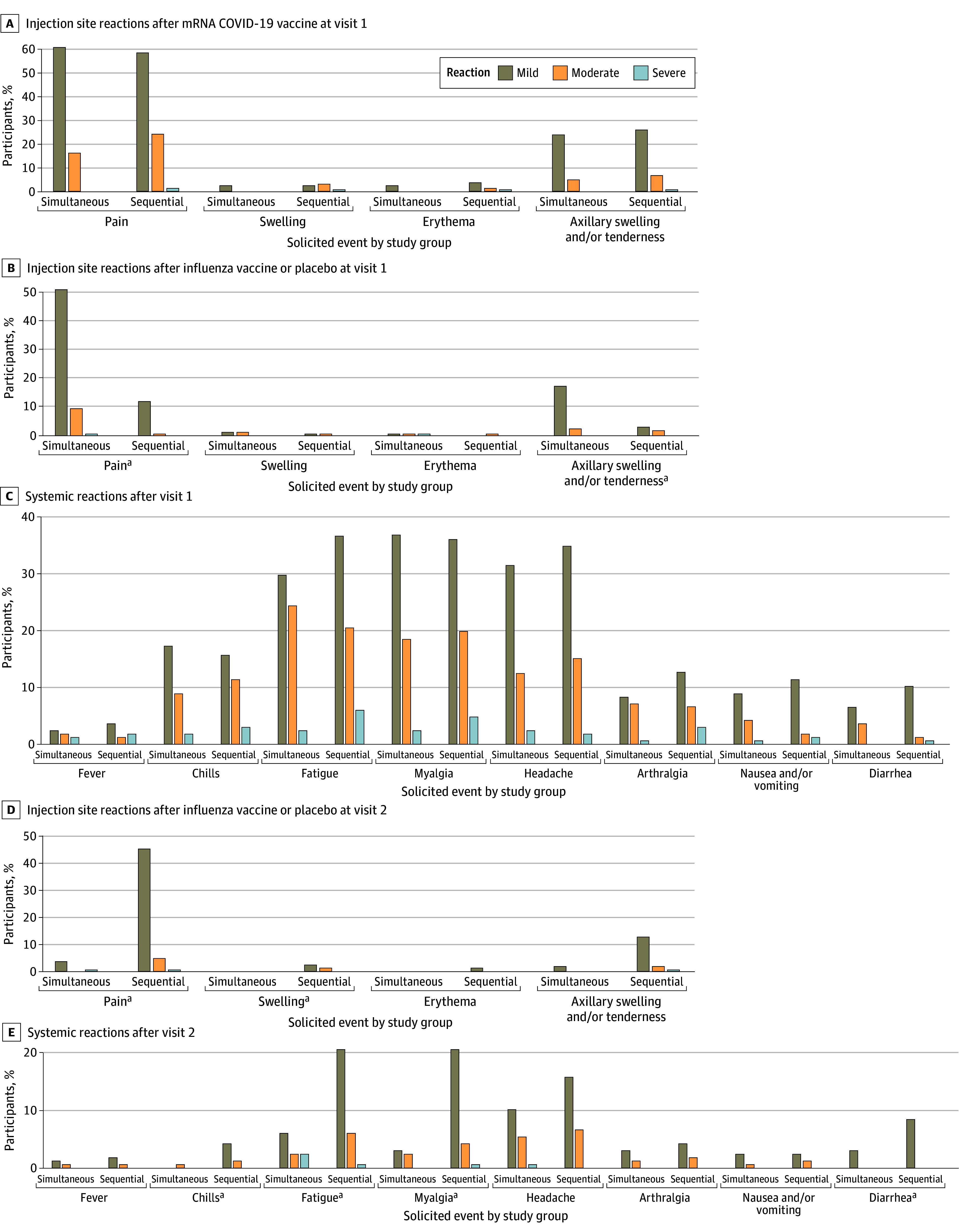
Injection Site and Systemic Reactogenicity Within 7 Days After Visit for the Full Analysis Population, by Severity The simultaneous group received messenger RNA (mRNA) COVID-19 and quadrivalent inactivated influenza vaccine at visit 1 and saline placebo at visit 2. The sequential group received mRNA COVID-19 vaccine and saline placebo at visit 1 and influenza vaccine at visit 2. Severity grading is described in eTable 1 in Supplement 2. ^a^The 95% CI of the difference in the percentages of participants with a reaction does not include 0.

Local (injection site) and systemic reactogenicity for both groups during the 7-day periods after visits 1 and 2 are presented in Figure 2 and eTables 1 and 2 in Supplement 2. Most injection site and systemic symptoms were mild to moderate; no life-threatening reactogenicity was reported. The mean (SD) durations for reactogenicity events were 2.4 (1.1) days or less except for arthralgia after visit 2, with a mean (SD) reported duration of 2.7 (2.9) days in the simultaneous group and 3.0 (2.3) days in the sequential group. In general, more participants reported local and systemic reactogenicity after visit 1 compared with visit 2. After visit 1, the percentages of participants in both groups with any local COVID-19 vaccine injection site or systemic reactogenicity symptoms were similar. However, when compared with those receiving placebo at visit 1, a higher percentage of participants receiving IIV4 at visit 1 reported pain at the injection site and axillary swelling and/or tenderness. Overall, approximately 10% to 25% of participants in our study reported moderate or severe injection site pain, fatigue, myalgia, headache, or chills following receipt of mRNA COVID-19 vaccine. After visit 2, when compared with those receiving placebo, a higher percentage of participants receiving IIV4 reported chills; fatigue; myalgia; diarrhea; and/or pain, induration, and/or swelling at the injection site, as well as axillary swelling and/or tenderness.

After visit 1, a higher percentage of participants in the sequential group reported moderate or severe COVID-19 vaccine injection site pain and swelling, while a higher percentage in the simultaneous group reported moderate or greater influenza vaccine or placebo injection site pain (eTable 1 in Supplement 2). After visit 2, a higher percentage of participants in the sequential group reported moderate or severe influenza vaccine or placebo injection site pain and axillary swelling and/or tenderness (eTable 2 in Supplement 2). No differences in the percentages of participants reporting moderate or severe systemic symptoms were observed after visit 1 or 2. This was true both among participants with a prior SARS-CoV-2 infection or who tested positive for nucleocapsid antibodies at baseline and among those without a prior SARS-CoV-2 infection and who tested negative for nucleocapsid antibodies. Across both visits, there were no significant differences in the percentages of participants reporting severe injection site or systemic reactogenicity. Fewer than 13% of participants in either group (simultaneous, 14 [8.3%]; sequential, 21 [12.6%]) had a severe reaction for any of the solicited reactions, and no participants sought medical attention for a solicited reaction.

### Unsolicited Adverse Events

In total, 45 unsolicited AEs were reported by 37 participants (11.0%) within the 7-day periods after the vaccination visits. The percentages of participants in the simultaneous and sequential groups reporting any AE within these periods were 21 (12.4%) and 16 (9.6%), respectively (difference, 2.8 pp; 95% CI, −3.9 to 9.5 pp). Three AEs deemed as either related or possibly related to vaccination occurred in each group (simultaneous: dizziness, lip lesion, and lip swelling; sequential: dizziness, presyncope, and injection site pruritus). One SAE occurred in each group; neither was considered to be related to vaccination (eTable 3 in Supplement 2). No significant differences in the percentages of participants in the simultaneous and sequential groups were observed with respect to the occurrence of SAEs (1 [0.6%] vs 1 [0.6%], respectively; difference, −0.01 pp [95% CI, −1.66 to 1.64 pp]).

Participant-reported AESIs were comparable between the simultaneous and sequential groups (19 [11.2%] vs 9 [5.4%], respectively; difference, 5.8 pp [95% CI, −0.1 to 11.7 pp]). All but 1 (3.6%) of the 28 AESIs was COVID-19 illness, with 18 participants (10.7%) in the simultaneous group reporting 19 illness events and 9 participants (5.4%) in the sequential group reporting 10 illness events; all but 1 of these participants (3.7%) had onset of COVID-19 illness more than 2 weeks after receipt of COVID-19 vaccine in the study, and all AESIs were considered to be of mild or moderate clinical severity. One person in the simultaneous group (0.6%) reported an allergic reaction with lip swelling noted on day 4 after visit 1, which was not considered anaphylaxis. This event resolved within a day and was deemed as possibly related.

### Health-Related Quality of Life

**Figure 3.**
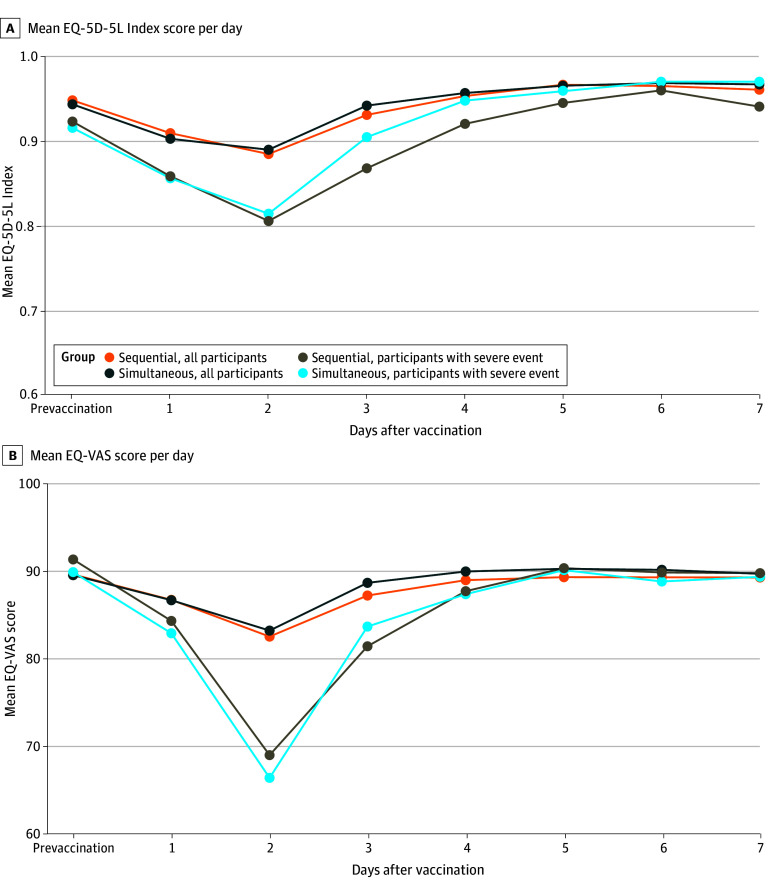
Mean Health-Related Quality-of-Life Scores in the 7 Days After Visit 1 At visit 1, the simultaneous group received the messenger RNA (mRNA) COVID-19 and quadrivalent inactivated influenza vaccine and the sequential group received the mRNA COVID-19 vaccine and saline placebo. Health-related quality of life was assessed by the EuroQol 5-Dimension 5-Level Index (EQ-5D-5L), which was scored from −0.109 to 1, and the EQ-5D-5L Visual Analogue Scale (EQ-VAS), which was scored from 0 to 100, with higher scores indicating better quality of life. Severe events were any grade 3 reactogenicity event.

There were no differences between the treatment groups in EQ-5D-5L Index and EQ-VAS scores during the week after visit 1 for all participants and for those who experienced any grade 3 reaction. For those with grade 3 reactions, the mean (SD) EQ-5D-5L Index score decreased from 0.92 (0.08) to 0.92 (0.09) prevaccination to 0.81 (0.09) to 0.82 (0.12) by day 2 and recovered to baseline by day 3 or 4 (Figure 3A). Likewise, the mean (SD) EQ-VAS score decreased from 89.9 (7.9) to 91.4 (5.0) prevaccination to 66.4 (24.2) to 69.0 (15.6) by day 2 and recovered to baseline by day 4 (Figure 3B).

## Discussion

To our knowledge, this is the first US placebo-controlled trial comparing simultaneous administration of inactivated influenza and mRNA COVID-19 vaccines. Simultaneous and sequential administration were comparable with respect to reactogenicity. The occurrence of at least 1 moderate or more severe reactogenicity event of fever, chills, myalgia, or arthralgia was noninferior (not higher) after simultaneous administration of mRNA COVID-19 and influenza vaccines at the same visit (25.6%) compared with sequential administration of mRNA COVID-19 vaccine and influenza vaccine (31.3%) on 2 visits spaced 1 to 2 weeks apart. Solicited reactions were short-lived, and no participant sought medical care for a reaction.

Approximately 10.7% to 26.8% of participants in the trial reported moderate or severe injection site pain, fatigue, myalgia, headache, or chills after receipt of mRNA COVID-19 vaccine, consistent with prelicensure trials.^22,23^ Overall, reactogenicity was similar to that observed in individuals aged 16 to 55 years receiving the primary original monovalent BNT162b2 COVID-19 vaccine series in a phase 3 clinical trial^22^ with the exception of myalgia, which we observed more frequently. Our trial queried for any muscle pain rather than new or increasing muscle pain, possibly contributing to the increase. More participants in the sequential group who received a placebo at visit 1 reported moderate or greater pain at the COVID-19 vaccine injection site. Receipt of influenza vaccine was associated with reactogenicity but at a lower frequency and severity than seen after mRNA COVID-19 vaccines and at a rate comparable to that described in the package insert.^21^ Participants in our trial reported axillary swelling and/or tenderness after both the COVID-19 and influenza vaccines; axillary swelling and tenderness have been reported after both vaccines.^29,30^

We did not note any statistically significant differences between simultaneous vs sequential administration in the occurrence of unsolicited AEs within 7 days of either vaccination visit, HRQOL within 7 days after the first vaccination visit, or SAEs or AESIs during the 121-day study period. We observed a numerical, but not statistically significant, imbalance in the number of persons having COVID-19 illness events in the simultaneous group vs the sequential group (18 vs 9). The reason for and clinical importance of this finding is unclear; assessment of comparative SARS-CoV-2 immunogenicity is under way.

Other studies describing the safety of simultaneous administration of influenza vaccine and COVID-19 vaccines have been reported.^31,32,33,34,35,36^ Unlike our small trial, which did not observe increased reactogenicity with simultaneous receipt of influenza vaccine, increased reactogenicity was noted in a large retrospective cohort study that evaluated simultaneous administration of a monovalent mRNA COVID-19 booster and seasonal influenza vaccine.^31^ Passive reporting to the US Vaccine Adverse Event Reporting System after simultaneous administration of monovalent mRNA COVID-19 and influenza vaccines did not reveal any notable patterns of adverse events.^32^ Additionally, in surveillance data comparing prespecified health outcomes between persons receiving COVID-19 and influenza vaccines simultaneously vs a COVID-19 vaccine alone in the CDC’s Vaccine Safety Datalink, a singular statistically significant elevated rate ratio was noted for the occurrence of Bell palsy when influenza vaccine was coadministered with a second dose of mRNA COVID-19 vaccine.^33^ However, this finding could only be confirmed in a small number of participants. Other, smaller observational studies assessing the outcomes of mRNA vaccine administered simultaneously with influenza vaccine did not find any AE concerns.^34,35^ A nested study where high-dose IIV4 was administered to older adults receiving either mRNA-1273 vaccine or placebo also found acceptable tolerability and safety when both vaccines were administered at the same time.^36^

We observed no differences between groups in the impact of reactogenicity on HRQOL as assessed by the EQ-5D-5L Index and EQ-VAS. We did note a minimal decline in mean index and VAS scores among all participants and a more clinically meaningful decline in mean index and VAS scores among participants with grade 3 reactions by day 2, but scores recovered to baseline by days 3 to 4. This pattern of decline and recovery in EQ-5D-5L Index and EQ-VAS scores has been observed among older adults with grade 3 reactogenicity after receiving a recombinant zoster vaccine.^37^

### Limitations

The present trial did not meet the targeted sample size and was underpowered to meet the noninferiority objective if our initial assumptions for the null hypothesis that the primary composite reactogenicity outcome would be more common in the simultaneous group had been correct. Furthermore, given the relatively small size, the trial was limited with respect to assessing less common or rare clinical outcomes potentially of more clinical concern.^38^ We enrolled a small number of children and of adults older than 65 years and excluded individuals pregnant at enrollment; thus, our findings are not generalizable to these populations. A similar study during pregnancy is planned.^39^ Additionally, the study population was underrepresentative of some racial and ethnic groups, and therefore our findings also may not be generalizable to these populations. The BNT162b2 mRNA COVID-19 vaccine was predominantly used in our trial, and most of the vaccines administered were bivalent; however, some participants did receive a monovalent vaccine during the first season. The FDA approved and authorized updated COVID-19 vaccines (2023-2024 formulation) that include a monovalent component that corresponds to the Omicron variant XBB.1.5 of SARS-CoV-2; these vaccines were administered during the 2023-2024 season after our trial completed enrollment. Vaccine safety monitoring for the updated COVID-19 vaccines will continue.

## Conclusions

In this prospective, randomized, placebo-controlled clinical trial, safety, including reactogenicity, after simultaneous or sequential administration of mRNA COVID-19 and IIV4 vaccines was comparable. This trial lends support to the option of simultaneous administration of these vaccines, which is a strategy to achieve high levels of vaccination coverage during anticipated periods of increased influenza and SARS-CoV-2 virus transmission.

## Notes

Supplement 1.
Trial Protocol

Supplement 2. **eMethods.** Participant Eligibility Criteria and Participant Reactogenicity Assessments **eTable 1.** Local Injection Site and Systemic Reactions by Severity, Including Moderate or Greater Severity, Following Visit 1 **eTable 2.** Local Injection Site and Systemic Reactions by Severity, Including Moderate or Greater Severity, Following Visit 2 **eTable 3.** Description of Serious Adverse Events

Supplement 3.
Data Sharing Statement
