# Supplementary material for: Safety of Simultaneous vs Sequential mRNA COVID-19 and Inactivated Influenza Vaccines: A Randomized Clinical Trial
Source: JAMA Netw Open. 2024 Nov 6;7(11):e2443166. doi: 10.1001/jamanetworkopen.2024.43166 (PMC11541642; doi:10.1001/jamanetworkopen.2024.43166)
Supplement: Supplement 1. — Trial Protocol [file jamanetwopen-e2443166-s001.pdf]

Safety of Simultaneous versus Sequential Administration of mRNA COVID-19 Vaccines and Quadrivalent Inactivated Influenza (IIV4) in Adults, Adolescents and Children: A Randomized Observer Blinded Study

Short Title: Simultaneous mRNA COVID-19 and IIV4 Vaccination Study

Centers for Disease Control & Prevention
Clinical Immunization Safety Assessment (CISA) Project

Lead Site (Duke University) Principal Investigator: Emmanuel B. Walter, MD, MPH
Lead Site Sub-principal Investigator: Ken Schmader, MD

Contributing Site (Johns Hopkins University) Principal Investigator: Kawsar Talaat, MD

Contributing Site (Cincinnati Children's Hospital Medical Center) Principal Investigator: Elizabeth Schlaudecker, MD, MPH
Contributing Site Sub-principal Investigator: Mary Staat, MD, MPH

Centers for Disease Control and Prevention (CDC) Principal Investigator: Karen R. Broder, MD
CDC Sub-principal Investigator: Jonathan Duffy, MD, MPH

NCT #: NCT05028361

Version Number: 7.0
15 March 2023

STATEMENT OF COMPLIANCE

- This trial will be conducted in compliance with the protocol, the International Conference on Harmonization (ICH) Guideline E6—Good Clinical Practice (GCP), and the applicable guidelines and regulatory requirements from the United States (US) Code of Federal Regulations (CFR), 45 CFR Part 46.
- All study personnel with subject contact have completed Human Subjects Protection Training.

TABLE OF CONTENTS

1 BACKGROUND 10
1.1 Background 10
1.2 Summary and Rationale 18
2 STUDY OBJECTIVES AND OUTCOME MEASURES 19
Primary 19
Secondary 19
Exploratory 21
3 STUDY DESIGN 23
3.1 Main study design 23
3.2 Laboratory Studies 23
4 STUDY ENROLLMENT AND WITHDRAWAL 25
4.1 Subject Inclusion Criteria 25
4.2 Subject Exclusion Criteria 25
4.3 Temporary Delay Criteria (Visit 1, 2 and 3 (for primary COVID-19 vaccine series)) 26
4.4 Recruitment 26
4.5 Reasons for and Handling of Withdrawals 28
4.6 Termination of Study 28
5 STUDY SCHEDULE, PROCEDURES, & EVALUATIONS 28
5.1 Schedule of events and data collection 28
5.2 Treatment Assignment Procedures 41
5.2.1 Randomization 41
5.3 Data Collection 41
5.3.1 Study Product Supply, Storage, and Administration 41
5.3.2 Blinding 42
5.4 Reactogenicity and Safety Assessment 43
5.4.1 Causality (relatedness) Assessment 47
5.4.2 Reporting of Adverse Events 47
5.4.3 Safety Monitoring Plan 47
5.5 Health-Related Quality of Life (HRQOL) 48
5.5.1 Generic Measures of HRQOL 48
5.6 Biospecimens Collection & Handling 49
5.6.1 Serum 49
6 LABORATORY ANALYSES 49
6.1 Baseline SARS-CoV-2 antibody 49
6.2 Influenza Hemagglutination Inhibition (HAI) Assay 49
6.3 COVID-19 Immunogenicity 50
6.4 Future Studies 51
7 STATISTICAL CONSIDERATIONS 51

7.1 Analysis Plan 51
7.1.1 Sample Size 51
7.1.2 Analysis Populations 52
7.1.3 Primary Objective 1 52
7.1.4 Secondary Objective 1 53
7.1.5 Secondary Objective 2 53
7.1.6 Secondary Objective 3 53
7.1.7 Secondary Objective 4 54
7.1.8 Exploratory Objectives 54
7.2 Data Management 54
7.2.1 Research Electronic Data Capture (REDCap) 54
7.3 Role of the CDC Investigators in the Project 55

8 HUMAN SUBJECTS 55

8.1 Human Subjects Involvement, Characteristics, and Design 55
8.2 Sources of Material 56
8.3 Potential Risks and Benefits 56
8.4 Adequacy of Protection Against Risks 57
8.4.1 Protections against Risk 58
8.4.2 ClinicalTrials.gov Requirements 58
8.5 Human Subjects 59
8.5.1 Vulnerable Subjects Research 59

REFERENCES 60

APPENDIX 64

Appendix A: EQ-5D-5L and VAS 65
Appendix B: Corticosteroid Dose Charts 66

Protocol Summary

**Title:**	Safety of Simultaneous versus Sequential Administration of mRNA COVID-19 Vaccines and Quadrivalent Inactivated Influenza (IIV4) in Adults, Adolescents and Children: A Randomized Observer Blinded Study
**Phase:**	Phase IV
**Population:**	450 persons ≥5 years of age for whom both COVID-19 and influenza vaccines are indicated and for whom COVID-19 vaccine is recommended by the ACIP.
**Clinical Sites:**	Three: Duke University (Lead); Johns Hopkins University (Contributing); Cincinnati Children's Hospital (Contributing)
**Study Duration:**	36 months total<br>• 12 months to recruit/enroll, 6 months during each of two influenza seasons<br>5 months to perform analysis and laboratory assays after the end of follow-up period
**Participant Duration and Number of Participants:**	• 120 days for 450 total participants at 3 sites
**Description of Study Procedures:**	This study is a prospective, randomized clinical trial. During this study, participants will be randomly assigned to receive quadrivalent inactivated influenza vaccine (IIV4) and mRNA COVID-19 vaccine either simultaneously or sequentially, 7-14 days apart. For participants receiving their primary dose series, a second dose of mRNA COVID-19 vaccine will be administered either 3 to 8 weeks or 4 to 8 weeks following the first dose, depending upon the mRNA COVID-19 vaccine provided. Participants receiving a booster dose of mRNA COVID-19 vaccine will receive a single COVID-19 dose within the study per current CDC guidelines.<br><br>Solicited symptoms of reactogenicity will be assessed on vaccination day and daily during the 7 days following each Vaccination Visit using either electronic or paper symptoms diaries, depending on study participant preference. Quality of life data will be collected using electronic or paper diaries on day of Vaccination Visit 1 and daily during the 7 days following the visit. Serious adverse events and adverse events of special interest will be collected throughout the duration of the study.

	Serum samples from participants will be collected for determination of COVID-19 serostatus at baseline. Serum samples will be taken throughout the study to determine IIV4 immunogenicity and for potential future studies.
**Objectives:**	Primary Objective:<br>- To compare the proportion of participants with moderate or more severe fever, chills, myalgia, or arthralgia in the group receiving IIV4 simultaneously with mRNA COVID-19 vaccine at Vaccination Visit 1 (Simultaneous group) with the group receiving IIV4 alone one to two weeks later at Vaccination Visit 2 (Sequential group) following both Vaccination Visit 1 and 2<br><br>Secondary Objective:<br>- To compare the proportion of participants with moderate or more severe fever, chills, myalgia, or arthralgia in the Simultaneous versus the Sequential Group following the first vaccination visit<br>- To compare the proportion of participants with moderate or more severe fever, chills, myalgia, or arthralgia in the Simultaneous versus Sequential Group following the second vaccination visit<br>- To describe the proportions of participants in the Simultaneous and Sequential vaccination groups with solicited local and systemic reactogenicity events according to severity grade after the first and second vaccination visit and third vaccination visit for those receiving two doses of mRNA COVID-19 vaccine<br>- To describe the proportions of participants in the Simultaneous and Sequential vaccination groups experiencing at least one serious adverse event and a description of these events<br><br>Exploratory Objective:<br>- To compare the proportion of participants with moderate or more severe fever, chills, myalgia, or arthralgia in the Simultaneous versus Sequential Group following the third vaccination visit for those receiving two doses of mRNA COVID-19 vaccine<br>- To further characterize and describe the proportion of participants in the Simultaneous and Sequential groups with local or systemic reactogenicity events of greater severity following each vaccination visit and cumulatively<br>- To describe the proportion of participants in Simultaneous and Sequential groups experiencing at

	least one unsolicited adverse event and one adverse event of special interest and to characterize these events<br>- To compare the change of health related quality of life (HRQOL) from baseline in the Simultaneous versus Sequential groups following the Vaccination Visit 1<br>- To assess the safety profiles in the simultaneous and sequential group participants by COVID-19 serostatus at baseline<br>- To assess the safety profiles in the simultaneous and sequential group participants by vaccine product (Pfizer or Moderna).<br>- To assess the effect of simultaneous administration of IIV4 and COVID-19 vaccine on IIV4 immunogenicity as assessed by hemagglutination inhibition assay (HAI)<br>- To assess serum antibody levels to SARS-CoV-2 antigens
**Outcome Measures:**	Primary Outcome Measure:<br>- Comparison of the proportion of participants reporting at least one solicited reactogenicity event to include fever, chills, myalgia, or arthralgia of moderate or greater severity in the Simultaneous group with the Sequential group within 1-7 days following both Vaccination Visit 1 and Vaccination Visit 2<br><br>Secondary Outcome Measure:<br>- Comparison of the proportion of participants reporting at least one solicited reactogenicity event to include fever, chills, myalgia, or arthralgia of moderate or greater severity in the Simultaneous group with the Sequential group within 1-7 days following Vaccination Visit 1 visit during which participants receive either IIV4 or placebo with an mRNA COVID-19 vaccine<br>- Comparison of the proportion of participants reporting at least one solicited reactogenicity event to include fever, chills, myalgia, or arthralgia of moderate or greater severity in the Simultaneous group with the Sequential group within 1-7 days following Vaccination Visit 2 during which participants receive either IIV4 or placebo without an mRNA COVID-19 vaccine<br>- The proportion of participants in each vaccination group reporting specified solicited local and systemic reactogenicity events of any severity and by severity grade within 1-7 days following the first vaccination visit during which participants receive either IIV4 or placebo with an mRNA COVID-19 vaccine<br>- The proportion of participants in each vaccination group reporting specified solicited local and systemic

	reactogenicity events of any severity and by severity grade within 1-7 days following the second vaccination visit during which participants receive either IIV4 or placebo<br>- The proportion of participants in each vaccination group reporting specified solicited local and systemic reactogenicity events of any severity and by severity grade within 1-7 days following the third vaccination visit during which all participants receive an mRNA COVID-19 vaccine alone for those receiving two doses of mRNA COVID-19 vaccine<br>- The proportion of participants in Simultaneous and Sequential Groups with at least one serious adverse event occurring during the study period and a description of each event<br><br>Exploratory Outcome Measure:<br>- Comparison of the proportion of participants reporting at least one solicited reactogenicity event to include fever, chills, myalgia, or arthralgia of moderate or greater severity in the Simultaneous group with the Sequential group within 1-7 days following Vaccination Visit 3 during which all participants receive an mRNA COVID-19 vaccine alone for those receiving two doses of mRNA COVID-19 vaccine<br>- The proportion of participants reporting at least one moderate or greater or at least one severe or greater solicited local or systemic reactogenicity event in each vaccination group within 1-7 days following the first vaccination during which participants receive either IIV4 or placebo with an mRNA COVID-19 vaccine<br>- The proportion of participants reporting at least one moderate or greater or at least one severe or greater solicited local or systemic reactogenicity event in each vaccination group within 1-7 days following the second vaccination visit which participants receive either IIV4 or placebo<br>- The proportion of participants reporting at least one moderate or greater or at least one severe or greater solicited local or systemic reactogenicity event in each vaccination group within 1-7 days following the third vaccination visit during which all participants receive an mRNA COVID-19 vaccine alone for those receiving two doses of mRNA COVID-19 vaccine<br>- The proportion of participants reporting at least one moderate or greater or at least one severe or greater solicited local or systemic reactogenicity event in each

	vaccination group within 1-7 days following all vaccination visits combined<br>- The proportion of participants in each vaccination group with an unsolicited adverse event occurring during the 7 days post each vaccination visit according to severity and system organ classification<br>- The proportion of participants in each vaccination group with an adverse event of special interest occurring during the study period according to event type<br>- Comparison between vaccination groups of the mean maximal change in score from baseline on the EuroQOL 5 dimensions-5 level (EQ-5D-5L) and EuroQOL visual analogue scale (EQ VAS) within 1-7 days following the first vaccination visit<br>- To compare secondary outcomes 3a, 3b, 3c, and 4a according to baseline COVID-19 serostatus<br>- To compare secondary outcomes 3a, 3b, 3c, and 4a according to vaccine product (Pfizer or Moderna).<br>- The proportion of participants in each vaccination group with a seroprotective HAI titer (≥ 1:40) pre- and post-IIV4 immunization for each IIV4 antigen<br>- The proportion of participants in each vaccination group achieving seroconversion following IIV4 (an HAI titer > 1:40 following IIV4 if the baseline titer is < 1:10 or a four-fold rise in HAI titer if the baseline titer is > 1:10) for each IIV4 antigen<br>- The geometric mean HAI titer (GMT) for each IIV4 antigen pre- and post-IIV4 in each vaccination group<br>- The geometric mean fold rise (GMFR) in HAI titer for each vaccination group<br>- The proportion of participants in each vaccination group seropositive to SARS-CoV-2 variants tested prior to and following mRNA COVID-19 vaccine<br>- The geometric mean ID_{50} and ID_{80} titers of neutralizing antibodies for each SARS-CoV-2 variant tested pre- and post-mRNA COVID-19 vaccine in each vaccination group
**Estimated Time to Complete Enrollment:**	Approximately 6 months for enrollment for the 2021-2022 influenza season and 6 months for enrollment during the 2022-2023 influenza season

1 BACKGROUND

1.1 Background

COVID-19

Coronavirus disease 2019 (COVID-19) is caused by a coronavirus called Severe Acute Respiratory Syndrome Coronavirus-2 (SARS-CoV-2). COVID-19 was first noted in late 2019 among clusters of pneumonia cases occurring in Wuhan China. The virus spread globally, in a rapid manner, with the first case among a US resident reported in January 2020[1]. On March 11, the World Health Organization declared COVID-19 a pandemic[2]. During the course of 2020 and 2021, the pandemic occurred in multiple distinct waves in the US, with the most recent wave starting in late 2021 and persisting into early 2022. As of April 3rd, 2022, over 79 million COVID-19 cases and over 950,000 deaths have been reported in the United States[3], making this the worst pandemic to occur in this country since the 1918 Spanish flu. With the emergence of numerous viral variants including the B1.1.529 Omicron variant and B.1.617.2 Delta variant in addition to other variants being monitored, the current trajectory of the pandemic remains uncertain [4].

Typical symptoms of COVID-19 include fever, chills, cough, shortness of breath, difficulty breathing, fatigue, myalgia, body aches, headache, new loss of taste or smell, sore throat, congestion, runny nose, nausea, vomiting, and diarrhea. Complications of COVID-19 are varied and include pneumonia, acute respiratory failure, acute respiratory distress syndrome, acute liver, kidney and/or cardiac injury, secondary infections, septic shock, disseminated intravascular coagulation, blood clots, multisystem inflammatory syndrome in children and adults, rhabdomyolysis and neurologic symptoms. Among known risk factors for severe illness are advanced age, cancer, chronic kidney disease, chronic obstructive pulmonary disease, Down syndrome, obesity, serious heart conditions (heart failure, coronary artery disease or cardiomyopathies), immunocompromised state from solid organ transplant, sickle cell disease and type 2 diabetes mellitus, smoking and pregnancy [5].

Influenza

Epidemic influenza is a highly infectious disease that occurs annually throughout the temperate regions in both hemispheres in their respective winter months [6]. Influenza infection occurs in all age groups and is a major public health threat, as it has the ability to spread rapidly through populations and affect a large number of people. While infection rates are generally highest among school age children, the highest rates of serious morbidity and mortality occur among individuals over 65 years of age and in children less than 1 year of age [7]. Underlying medical conditions such as chronic cardiac, pulmonary, kidney and metabolic disorders, and various types of immunosuppression increase the likelihood of potential complications[8].

Uncomplicated influenza illness is characterized by the abrupt onset of constitutional symptoms including: fever, myalgia, headache, malaise, nonproductive cough, sore throat, and rhinitis. Influenza virus infection can also lead to more serious illness such as primary viral pneumonia and exacerbation of underlying medical conditions. Common complications include bacterial pneumonia, sinusitis, and otitis media. The estimated annual number of influenza-associated hospitalizations in the United States is 226,000 and the estimated annual number of deaths is

34,470 [9,10]. Hospitalizations due to influenza are typically more prevalent in seasons during which A(H3N2) subtype predominates.

COVID-19 and Influenza

In the spring of 2020 COVID-19 emerged late in the course of the 2019-2020 seasonal influenza epidemic, with co-infection being reported in some individuals[11]. Of potential concern, was the possibility of a simultaneous surge in both COVID-19 cases and influenza cases during the 2020-2021 season. However, influenza activity in the United States during the 2020-2021 season remained relatively low and to date has remained relatively low during the 2021-22 season. If both COVID-19 and influenza infections peaked simultaneously during subsequent seasons, there could be a significant future impact on utilization of health care resources potentially overburdening the health care system.

COVID-19 Vaccines

The Pfizer-BioNTech COVID-19 vaccine (BNT162b2) (marketed as Comirnaty) received FDA-licensure for individuals 16 years and older on August 23rd, 2021 for primary dose series. The Moderna COVID-19 vaccine (marketed as Spikevax) received FDA approval on January 31st, 2022 for individuals 18 years and older for primary dose series. Numerous other COVID-19 vaccines are currently in different phases of preclinical and clinical development. Six vaccines have recently completed or are currently undergoing late stage Phase 3 clinical testing in the United States including the two mRNA vaccines, two viral vectored vaccines, and two adjuvanted recombinant protein-based vaccines[12]. Prior to approval, the Moderna mRNA vaccine (mRNA-1273) received US Food and Drug Administration (FDA) Emergency Use Authorization (EUA) for persons 18 years of age and older[13], and the Pfizer-BioNTech COVID-19 vaccine received an initial EUA in December 2020 for persons 16 years of age and older and expanded its EUA to include individuals 12 -15 years of age on May 10, 2021 and individuals 5 - 11 years of age on October 29, 2021[14]. Both Pfizer-BioNTech and Moderna vaccines are based on the SARS-CoV-2 spike glycoprotein (S) antigen encoded by RNA and formulated in lipid nanoparticles (LNPs). Among study participants without evidence of SARS-CoV-2 infection, the Pfizer/BioNTech vaccine (BNT162b2) was 95.0% (95% CI 90.3%, 97.6%) effective at preventing confirmed COVID-19 occurring at least 7 days after the second dose of vaccine [15]. The Moderna vaccine (mRNA-1273) demonstrated a vaccine efficacy of 94.1% (95% CI 89.3%, 96.8%) for the prevention of symptomatic SARS-COV-2 infection with onset at least 14 days after the second injection, with 11 COVID-19 cases in the vaccine group and 185 COVID-19 cases in the placebo group [16]. The Janssen adenovirus 26 vectored COVID-19 vaccine (Ad26 COV2.S) received EUA from the FDA on February 27, 2021 but due to being a viral vector vaccine it will not be further addressed by this study evaluating the simultaneous administration of influenza and mRNA COVID-19 vaccines.

BNT162b2 is currently authorized under the FDA EUA for persons 5 – 15 years of age and approved for persons 16 years of age and older, while the mRNA-1273 is approved for persons 18 years of age and older. CDC's Advisory Committee on Immunization Practices (ACIP) has recommended use of these vaccines, according to the specifications in the EUAs; CDC has also published interim clinical considerations for use of COVID-19 vaccines[17,18]. Both vaccines are administered intramuscularly with the Pfizer-BioNTech vaccine (30 µg, 0.3mL each for persons

≥ 12 years of age and 10 µg, 0.2mL each for persons 5-11 years of age) being administered 21 days apart and the Moderna vaccine (100 µg, 0.5mL each) being administered 28 days apart as a primary series in particular, for populations that include moderately or severely immunocompromised, adults over 65 years of age and those who need rapid protection against COVID-19. Doses should not be administered earlier than recommended but if given within a 4-day period prior to the scheduled time may be considered valid. The second dose can be administered between 3 and 8 weeks after the first dose for the Pfizer- BioNTech vaccine and between 4 and 8 weeks after the first dose for the Moderna vaccine. The vaccines are not considered to be interchangeable, however under extenuating circumstances such as when the brand of the first dose of vaccine is unknown, any mRNA COVID-19 vaccine may be administered 28 days following the first dose[17].

On August 12, 2021, the FDA amended the Pfizer-BioNTech and Moderna mRNA COVID-19 vaccine EUAs to include the use of a third dose of vaccine in specific immunocompromised populations for persons 12 years of age and older and persons 18 years of age or older for the respective vaccines.[19] Additionally, the CDC also recommended that moderate or severely immune compromised populations receive an additional third dose of the mRNA COVID-19 vaccines to enhance the immune response[20]. Subsequent to these actions, the U.S. government has expanded authorizations and recommendations for a third dose of COVID-19 vaccine as infections rose from the coronavirus Delta variant and then the Omicron variant, citing data indicating diminishing protection from the vaccines over time. The FDA initially authorized a booster dose for both Pfizer-BioNTech and Moderna mRNA COVID-19 vaccines for all persons 18 years of age and older on November 19, 2021. Subsequent to this action, the EUA was extended for the Pfizer-BioNTech mRNA COVID-19 vaccine to include 16-17 year olds on December 9, 2021 and 12-15 year olds on January 3, 2022. The CDC has made comparable recommendations to follow the EUAs[21-24]. Both the EUA and recommendations for the interval between the primary series and booster dose have also been shorted to 5 months. On March 29, 2022 FDA authorized a second booster dose for persons aged 50 and older and certain immunocompromised individuals; CDC updated guidance for second booster doses, with a 4-month interval between a first and second booster dose[13,17].

Local and systemic side effects occurring within the week after vaccination as reported from the BNT162b2 Phase2/3 reactogenicity subset are noted in Table 1. In general, both local and systemic side effects were reported more frequently in those who were younger and following the second dose of vaccine.

Table 1. BNT162b2 Reactogenicity (Phase 2/3) [25]

	Dose 1		Dose 2	
	**16-55 years**	**>55 years**	**16-55 years**	**>55 years**
**Local**				
Pain	83.1%	71.1%	77.8%	66.1%
Redness	4.5%	4.7%	5.9%	7.2%
Swelling	5.8%	6.5%	6.3%	7.5%
**Systemic**				
Fever	3.7%	1.4%	15.8%	10.9%
Fatigue	47.4%	34.1%	59.4%	50.5%
Headache	41.9%	25.2%	51.7%	39.0%

Chills	14.0%	6.3%	35.1%	22.7%
Vomiting	1.2%	0.5%	1.9%	0.7%
Diarrhea	11.1%	8.2%	10.4%	8.3%
Muscle pain	21.3%	13.9%	37.3%	28.7%
Joint pain	11.0%	8.6%	21.9%	18.9%

Overall, the frequency of any severe systemic event after Dose 1 was ≤0.9% or less. After Dose 2, severe systemic events had frequencies of <2% with the exception after Dose 2 of fatigue (3.8%) and headache (2.0%). Severe fever (>38.9°C to 40.0°C) was reported in the BNT162b2 group after Dose 1 for 0.2% and after Dose 2 for 0.8% [15].

A higher frequency of unsolicited, non-serious adverse events was reported in the vaccine group compared to placebo group and was primarily attributed to local reactions and systemic adverse events in subjects not in the reactogenicity subset and are consistent with solicited reactions/events reported by reactogenicity subset participants during the first 7 days following vaccination. In the all-enrolled population of (total N=43,448), the proportions of participants who reported at least 1 serious adverse event (SAE) during the time period from Dose 1 to the data cutoff date (November 14, 2020) were 0.6% in the BNT162b2 vaccine group and 0.5% in the placebo group. Three SAEs reported in the BNT162 group were considered by the investigator as related to vaccine or vaccine administration: shoulder injury, ventricular arrhythmia, and lymphadenopathy.

Local and systemic side effects as reported from the mRNA-1273 Phase 3 reactogenicity subset are noted in **Table 2**. Similar to the Pfizer-BioNTech vaccine, in general, both local and systemic side effects were reported more frequently in those who were younger and following the second dose of vaccine.

Table 2. mRNA-1273 Reactogenicity (Phase 3) [16]

	**Dose 1**		**Dose 2**	
	**18-<64 years**	**≥65 years**	**18-<64 years**	**≥65 years**
**Local**				
Any	87.4%	74.6%	90.3%	83.8%
Pain	86.9%	74.0%	89.9%	83.2%
Redness	3.0%	2.3%	8.9%	7.5%
Swelling	6.7%	4.4%	12.6%	10.8%
Axillary swelling and tenderness of the vaccination arm	11.6%	6.1%	16.2%	8.6%
**Systemic**				
Any	57.0%	48.3%	81.9%	71.9%
Fever	0.9%	0.3%	17.4%	10.0%
Headache	35.3%	24.5%	62.8%	46.2%
Fatigue	38.4%	33.3%	67.6%	58.3%
Myalgia	23.7%	19.7%	61.6%	47.1%

Table 2. mRNA-1273 Reactogenicity (Phase 3) [16]				
	Dose 1		Dose 2	
	18-<64 years	≥65 years	18-<64 years	≥65 years
Arthralgia	16.6%	16.4%	45.5%	35.0%
Nausea and Vomiting	9.4%	5.2%	21.4%	11.8%
Chills	9.2%	5.4%	48.6%	30.9%

Overall, the frequency of any Grade 3 systemic event after Dose 1 ranged from 2.0 to 2.9% depending upon age. After Dose 2, Grade 3 systemic events were reported by 15.7% in the younger group and 2.0% in the older group[26] .

The proportion of participants who reported severe unsolicited AEs was 1.4% following any vaccine dose (275 participants) and 1.3% following any placebo dose (225 participants). The most frequently reported severe AEs that occurred in greater numbers of vaccine than placebo recipients were headache, myalgia, arthralgia, injection site erythema, and injection site pain. The frequency of non-fatal serious adverse events was low and without meaningful imbalances between study arms (1% in the mRNA-1273 group and 1% in the placebo group).

A more detailed comparison of selected systemic reactogenicity events and their grading following Dose 1 of mRNA-1273 and BNT162b2 is included below (**Table 3)**:

Table 3. Selected Systemic Reactogenicity Events and Grading following BNT162b2 and mRNA-1273				
**Systemic Symptom**	**Grade**	**BNT162b2 [15 25]**	**Grade**	**mRNA-1273[16]**
		**18 to 55 years of age**		**18-<65 years**
Fever				
	> 38.0°C	3.7%	≥ 38.0°C	0.9%
	> 38.0°C to 38.4°C	2.8%	38.0°C to 38.4°C	0.6%
	> 38.4°C to 38.9°C	0.7%	38.5°C to 38.9°C	0.2%
	> 38.9°C to 40.0°C	0.3%	> 39.0°C to 40.0°C	<0.1%
	> 40.0°C	0.0%	> 40.0°C	<0.1%
	> 38.4°C	*1.0%*	*≥ 38.5°C*	*≈0.4%*
Chills	Any	14.0%	Any	9.2%
	Mild	10.0%	Grade 1	6.8%
	Moderate	3.6%	Grade 2	2.2%
	Severe	0.4%	Grade 3	0.1%
	Moderate or Severe	*4.0%*	*Grade 2 or 3*	*2.3%*
+*				

Table 3. Selected Systemic Reactogenicity Events and Grading following BNT162b2 and mRNA-1273				
**Systemic Symptom**	**Grade**	**BNT162b2** [15 25]	**Grade**	**mRNA-1273**[16]
		**18 to 55 years of age**		**18-<65 years**
Muscle pain	Any	21.3%	Any	23.7%
	Mild	11.2%	Grade 1	16.5%
	Moderate	9.5%.	Grade 2	6.6%
	Severe	0.6%	Grade 3	0.6%
	Moderate or Severe	*10.1%*	*Grade 2 or 3*	*7.2%*
Joint pain	Any	11.0%	Any	16.6%
	Mild	6.4%	Grade 1	12.0%
	Moderate	4.3%	Grade 2	4.2%
	Severe	0.2%	Grade 3	0.4%
			Grade 4	<0.1%
	Moderate or Severe	*4.5%*	*Grades 2,3 or 4*	*≈4.7%*

In a study comparing the safety and immunogenicity of BNT162b2 vaccine in adolescents to that in adults local and systemic events were generally mild to moderate in severity and were reported at similar frequencies in both age cohorts. As with adults, systemic events were reported more often after BNT162b2 dose 2 than after dose 1. The vaccine produced a greater immune response in adolescents than adults and was highly effective[27].

A study assessing two 10 µg doses of BNT162b2 in children 5 to 11 years of age each administered 3 weeks apart, showed the vaccine to be safe, immunogenic and efficacious in this age group as well. Solicited injection site and systemic reactions were generally mild to moderate and lasted 1 to 2 days. In general, systemic events were reported more commonly after the second dose than the first dose. Fever occurred in 8.3% of recipients after the first and second dose.[28]

Hypersensitivity Reactions and mRNA COVID-19 Vaccines
In the Pfizer mRNA phase III trial, more participants reporting hypersensitivity-related adverse events in the vaccine group (137 [0.63%]) compared with the placebo group (111 [0.51%]). There were no anaphylactic or severe hypersensitivity reactions reported (FDA EUA).

In the Moderna mRNA COVID vaccine phase III trial, more participants reported hypersensitivity-related adverse events in the vaccine group 1.5% of vaccine recipients (258 events in 233 participants) compared with the placebo group 1.1% of placebo recipients (185 events in 166 participants). There were no anaphylactic or severe hypersensitivity reactions with close temporal relation to the vaccine. There was one anaphylactic reaction occurring 63 days after the second dose of the vaccine that did not meet the Brighton Collaboration Anaphylaxis Case Definition (FDA EUA).

There have been numerous reports of anaphylactic reactions after the Pfizer and Moderna vaccine after the start of vaccination campaigns (MMWR January 22, 2021, Vol. 70; MMWR January 15, 2021, Vol. 70). The update on anaphylaxis reports to VAERS following mRNA COVID Vaccines through January 18, 2021 (January ACIP meeting), showed following results: **Table 4**. Anaphylaxis reports to VAERS following COVID-19 vaccines.

Table 4. BNT162b2 and Moderna Anaphylactic Reactions		
Characteristic	Pfizer-BioNTech (N = 50)	Moderna (N = 21)
Median age, years (range)	38.5 (26-63)	39 (24-63)
Female (%)	47 (94)	21 (100)
Minutes to symptom onset, median	10	10
Symptom onset ≤15 minutes (%)	37 (74)	18 (86)
Symptom onset ≤30 minutes (%)	45 (90)	19 (90)
Documented h/o of allergies	40 (80)	18 (86)
Documented h/o of prior anaphylaxis	12 (24)	5 (24)
Dose number (1st 2nd, unk)	42, 3, 5	19, 1, 1

The anaphylaxis reporting rate (analytic period Dec 14 – Jan 18) for the Pfizer mRNA vaccine is 4.7 per million doses and for Moderna mRNA vaccine, 2.5 per million doses[29]. Both mRNA vaccines include a warning that anaphylaxis may occur after mRNA vaccines and that appropriate medical treatment for management must be immediately available[30,31].

Myocarditis and Pericarditis and mRNA COVID-19 Vaccines

Since April 2021, increased cases of myocarditis and pericarditis have been reported in the United States after mRNA COVID-19 vaccination (Pfizer-BioNTech and Moderna), particularly in adolescents and young adults after the second dose[32]. In June 2021, the FDA updated the EUAs for both mRNA COVID-19 vaccines to describe the risk for myocarditis and pericarditis after mRNA COVID-19 vaccines[30,31].

While absolute risk remains small, evidence suggests the risk might be reduced by extending the interval between the first and second dose. Studies in adolescents (ages 12-17 years) and adults have shown the small risk of myocarditis associated with mRNA COVID-19 vaccines might be reduced and peak antibody responses and vaccine effectiveness may be increased with an interval up to 8 weeks following the initial dose. There are currently no data available for children ages 11 years and younger. A 3- or 4-week interval continues to be the recommended interval for people who are moderately to severely immunocompromised, adults ages 65 years and older, and others who need rapid protection due to increased concern about community transmission or risk of severe disease[33].

Booster Doses

The safety of a booster dose (dose 3) of the Pfizer COVID-19 vaccine was assessed in about 300 adults aged 18-55 years. The reactogenicity and safety profile observed after the booster dose was generally similar to that observed after Dose 2 of the initial two-dose vaccination series. Lymphadenopathy was more frequent after dose 3 (5.2%), compared with 0.4% in persons aged ⩾16 years after dose 1 or 2. Most cases had axillary lymphadenopathy that resolved within 5 days after onset.[13,34] The safety of a booster (dose 3) of the Moderna COVID-19 vaccine at half the dose (50ug) was assessed in 168 persons over 18 years of age. The reactogenicity and safety profile observed after the booster dose was generally similar to that observed after Dose 2 of the initial two-dose vaccination series[35]. FDA identified no new safety concerns following second booster dose of Pfizer COVID-19 vaccine administered to older adults[13,36].

Influenza and Influenza Vaccine

Inactivated influenza virus vaccines (IIVs) have been available in the United States for over 50 years and influenza vaccination remains the most effective strategy for reducing the burden of disease caused by influenza. In the United States, vaccination of all persons aged 6 months and older is recommended on an annual basis[37]. In the 2021-22 influenza season all influenza vaccine formulations are quadrivalent (IIV4) and contain hemagglutinin (HA) from four prototypic influenza types/subtypes that commonly circulate including: influenza A (H1N1pdm09), influenza A (H3N2), and 2 different influenza B lineages (B Yamagata and B Victoria) [37]. For adults 65 years of age and older, who may not develop as robust an immune response as younger individuals, both high dose and adjuvanted formulations of IIV4 have been developed. High dose and adjuvanted formulations have been shown to be more immunogenic when compared to standard dose formulations in older adults[8]. High-dose influenza vaccine has also been shown to be more effective than standard dose influenza vaccine in a randomized clinical trial.

On the basis of review of the evidence concerning high-dose inactivated influenza vaccine (HD-IIV), recombinant influenza vaccine (RIV), and MF59-adjuvanted inactivated influenza vaccine (aIIV), the CDC's Advisory Committee on Immunization Practices (ACIP) recently modified recommendations for influenza vaccination of persons aged ≥65 years[38]. ACIP now recommends that adults aged ≥65 years preferentially receive any one of the following higher dose or adjuvanted influenza vaccines: quadrivalent high-dose inactivated influenza vaccine (HD-IIV4), quadrivalent recombinant influenza vaccine (RIV4), or quadrivalent adjuvanted inactivated influenza vaccine (aIIV4). If none of these three vaccines is available at an opportunity for vaccine administration, then any other age-appropriate influenza vaccine should be used. Higher dose vaccines include HD-IIV4 and RIV4, both of which contain a higher dose of HA antigen per virus than standard-dose vaccines (60 μg for HD-IIV4 and 45 μg for RIV4, compared with 15 μg for standard-dose inactivated vaccines). Adjuvanted inactivated influenza vaccine (aIIV4) contains MF59 adjuvant.

Side effects after an inactivated influenza vaccine may include soreness, redness and swelling at the injection site, fever, muscle aches, and headache. There also may be a very small increased risk of Guillain- Barré Syndrome (GBS) or anaphylaxis after an inactivated influenza vaccine[39].

1.2 Summary and Rationale

In clinical practice, the FDA approved the Pfizer-BioNTech and Moderna COVID-19 vaccines and granted an Emergency Use Authorization for Janssen COVID-19 vaccine in February 2021. Hence, vaccines for both COVID-19 and influenza prevention have the potential for being administered at the same time seasonal influenza vaccine is available. In the pre-licensure efficacy studies, neither the Pfizer-BioNTech or Moderna mRNA vaccines were evaluated for either safety or immunogenicity when co-administered with a non-COVID-19 vaccine. The Phase 3 trial of the AstraZeneca chimpanzee adenovirus-vectored COVID-19 vaccine did not prohibit influenza vaccination but simultaneous administration was not systematically evaluated. Initial guidance from the CDC stated that COVID-19 vaccines should be given alone with at least 14 days either before or after receipt of any other vaccines, including an influenza vaccine, because of limited information on the safety and effectiveness of co-administration. Current guidance now states that COVID-19 vaccines can be administered on the same day as other vaccines but mentions that it is unknown if simultaneous administration will affect reactogenicity[17,37,40]. A single study has assessed the safety and immunogenicity of a 3rd dose of Moderna's mRNA COVID-19 vaccine and High-Dose quadrivalent influenza vaccine when administered simultaneously to adults 65 years and older. No safety concerns or immune interference were observed.[41]

Due to the potential for severe influenza complications and resulting morbidity and mortality, it remains particularly important that influenza vaccine is encouraged for all persons 6 months of age and older without any contraindications. Likewise, as the pandemic continues and COVID-19 vaccines become available it is critically important that their use be encouraged in those populations for whom there is an authorization and an ACIP recommendation. To date, we are unaware of any studies evaluating the safety and immune responses following concomitant administration of both mRNA COVID-19 and influenza vaccines in a more general population. Hence, we aim to study the safety of simultaneous versus sequential administration of mRNA COVID-19 vaccine and IIV4. Participants receiving mRNA COVID-19 vaccines as either standard of care or a study procedure will be randomized to receive either IIV4 or placebo within 8 hours of their initial dose of mRNA COVID-19 vaccine. Those receiving IIV4 will receive placebo 14 days later and those receiving placebo will receive IIV4 14 days later.

We have elected to evaluate the mRNA COVID-19 vaccines for evaluations for three reasons: 1) there is greater accumulated experience with using mRNA COVID-19 vaccines in the population; 2) the different reactogenicity profiles for the mRNA COVID-19 vaccines as compared to the Janssen adenoviral vector COVID-19 vaccine decreases the ability of a single small study to address the question of co-administration safety[13] and 3) the preferential recommendation for the use of mRNA COVID-19 vaccines over the Janssen vaccine for both primary and booster vaccination due to concerns related to thrombosis with thrombocytopenia syndrome following Janssen COVID-19 vaccine[42]. In addition, we hope to assess the effects of

pre-existing COVID-19 antibody before vaccination on the occurrence of vaccine side effects and to assess the effects of COVID-19 vaccine administration on IIV4 immunogenicity.

The current protocol allows for inclusion of persons receiving a booster dose of mRNA COVID-19 vaccine. Administration of booster dose mRNA COVID-19 vaccines to participants who are enrolled in this protocol, will adhere to current FDA prescribing recommendations for licensed or EUA mRNA COVID-19 vaccines and to ACIP recommendations for their use as the approvals and recommendations are made. Inclusion of persons receiving a booster dose of COVID-19 vaccine will allow for enhanced recruitment during the influenza vaccination season in which it is expected that most adults and many children receiving an mRNA COVID-19 vaccine in this study will be receiving a booster dose rather than an initial vaccine two-dose series.

2 STUDY OBJECTIVES AND OUTCOME MEASURES

OBJECTIVE	OUTCOME
**Primary**	
1. To compare the proportion of participants with moderate or more severe fever, chills, myalgia, or arthralgia in the group receiving IIV4 simultaneously with mRNA COVID-19 vaccine at Vaccination Visit 1 (Simultaneous group) with the group receiving IIV4 alone one to two weeks later at Vaccination Visit 2 (Sequential group) following both Vaccination Visit 1 and 2<br>Hypothesis: The proportion of participants with moderate or more severe fever, chills, myalgia or arthralgia will be noninferior (not higher) in the Simultaneous group versus the Sequential group.	1a. Comparison of the proportion of participants reporting at least one solicited reactogenicity event to include fever, chills, myalgia, or arthralgia of moderate or greater severity in the Simultaneous group with the Sequential group within 1-7 days following both Vaccination Visit 1 and Vaccination Visit 2
**Secondary**	
1. To compare the proportion of participants with moderate or more severe fever, chills, myalgia, or arthralgia in the Simultaneous versus the Sequential Group following the first vaccination visit	1a. Comparison of the proportion of participants reporting at least one solicited reactogenicity event to include fever, chills, myalgia, or arthralgia of moderate or greater severity in the Simultaneous group with the Sequential group within 1-7 days following Vaccination Visit 1 visit during which participants receive either IIV4 or placebo with an mRNA COVID-19 vaccine

2. To compare the proportion of participants with moderate or more severe fever, chills, myalgia, or arthralgia in the Simultaneous versus Sequential Group following the second vaccination visit	2a. Comparison of the proportion of participants reporting at least one solicited reactogenicity event to include fever, chills, myalgia, or arthralgia of moderate or greater severity in the Simultaneous group with the Sequential group within 1-7 days following Vaccination Visit 2 during which participants receive either IIV4 or placebo without an mRNA COVID-19 vaccine
3. To describe the proportions of participants in the Simultaneous and Sequential vaccination groups with solicited local and systemic reactogenicity events according to severity grade after the first and second vaccination visit and third vaccination visit for those receiving two doses of mRNA COVID-19 vaccine	3a. The proportion of participants in each vaccination group reporting specified solicited local and systemic reactogenicity events of any severity and by severity grade within 1-7 days following the first vaccination visit during which participants receive either IIV4 or placebo with an mRNA COVID-19 vaccine<br><br>3b. The proportion of participants in each vaccination group reporting specified solicited local and systemic reactogenicity events of any severity and by severity grade within 1-7 days following the second vaccination visit during which participants receive either IIV4 or placebo<br><br>3c. The proportion of participants in each vaccination group reporting specified solicited local and systemic reactogenicity events of any severity and by severity grade within 1-7 days following the third vaccination visit during which all participants receive an mRNA COVID-19 vaccine alone for those receiving two doses of mRNA COVID-19 vaccine
4. To describe the proportions of participants in the Simultaneous and Sequential vaccination groups experiencing at least one serious adverse event and a description of these events	4a. The proportion of participants in Simultaneous and Sequential Groups with at least one serious adverse event occurring during the study period and a description of each event

**Exploratory**	
1. To compare the proportion of participants with moderate or more severe fever, chills, myalgia, or arthralgia in the Simultaneous versus Sequential Group following the third vaccination visit for those receiving two doses of mRNA COVID-19 vaccine	1a. Comparison of the proportion of participants reporting at least one solicited reactogenicity event to include fever, chills, myalgia, or arthralgia of moderate or greater severity in the Simultaneous group with the Sequential group within 1-7 days following Vaccination Visit 3 during which all participants receive an mRNA COVID-19 vaccine alone for those receiving two doses of mRNA COVID-19 vaccine
2. To further characterize and describe the proportion of participants in the Simultaneous and Sequential groups with local or systemic reactogenicity events of greater severity following each vaccination visit and cumulatively	2a. The proportion of participants reporting at least one moderate or greater or at least one severe or greater solicited local or systemic reactogenicity event in each vaccination group within 1-7 days following the first vaccination during which participants receive either IIV4 or placebo with an mRNA COVID-19 vaccine<br><br>2b. The proportion of participants reporting at least one moderate or greater or at least one severe or greater solicited local or systemic reactogenicity event in each vaccination group within 1-7 days following the second vaccination visit which participants receive either IIV4 or placebo<br><br>2c. The proportion of participants reporting at least one moderate or greater or at least one severe or greater solicited local or systemic reactogenicity event in each vaccination group within 1-7 days following the third vaccination visit during which all participants receive an mRNA COVID-19 vaccine alone for those receiving two doses of mRNA COVID-19 vaccine<br><br>2d. The proportion of participants reporting at least one moderate or greater or at least one severe or greater solicited local or systemic reactogenicity event in each vaccination group within 1-7 days following all vaccination visits combined

3. To describe the proportion of participants in Simultaneous and Sequential groups experiencing at least one unsolicited adverse event and one adverse event of special interest and to characterize these events	3a. The proportion of participants in each vaccination group with an unsolicited adverse event occurring during the 7 days post each vaccination visit according to severity and system organ classification<br><br>3b. The proportion of participants in each vaccination group with an adverse event of special interest occurring during the study period according to event type
4. To compare the change of health-related quality of life (HRQOL) from baseline in the Simultaneous versus Sequential groups following the Vaccination Visit 1	4a. Comparison between vaccination groups of the mean maximal change in score from baseline on the EuroQOL 5 dimensions-5 level (EQ-5D-5L) and EuroQOL visual analogue scale (EQ VAS) within 1-7 days following the first vaccination visit
5. To assess the safety profiles in the simultaneous and sequential group participants by baseline COVID-19 serostatus	5a. To compare secondary outcomes 3a, 3b, 3c, and 4a according to baseline COVID-19 serostatus
6. To assess the safety profiles in the simultaneous and sequential group participants by vaccine product (Pfizer or Moderna).	6a. To compare secondary outcomes 3a, 3b, 3c, and 4a according to vaccine product (Pfizer or Moderna).
7. To assess the effect of simultaneous administration of IIV4 and COVID-19 vaccine on IIV4 immunogenicity as assessed by hemagglutination inhibition assay (HAI)	7a. The proportion of participants in each vaccination group with a seroprotective HAI titer (≥ 1:40) pre- and post-IIV4 immunization for each IIV4 antigen<br><br>7b. The proportion of participants in each vaccination group achieving seroconversion following IIV4 (an HAI titer ≥ 1:40 following IIV4 if the baseline titer is < 1:10 or a four-fold rise in HAI titer if the baseline titer is ≥ 1:10) for each IIV4 antigen<br><br>7c. The geometric mean HAI titer (GMT) for each IIV4 antigen pre- and post-IIV4 in each vaccination group<br><br>7d. The geometric mean fold rise (GMFR) in HAI titer for each vaccination group
8. To assess serum antibody levels to SARS-CoV-2 antigens	8a. The proportion of participants in each vaccination group seropositive to SARS-CoV-2 variants tested prior to and following mRNA COVID-19 vaccine

	8b. The geometric mean ID_{50} and ID_{80} titers of neutralizing antibodies for each SARS-CoV-2 variant tested pre- and post-mRNA COVID-19 vaccine in each vaccination group

3 STUDY DESIGN

3.1 Main study design

This study is a prospective, randomized, clinical trial to assess the safety of simultaneous versus sequential administration of mRNA COVID-19 and IIV4 vaccines in 450 persons age ≥5 years enrolled at Duke University Medical Center (Lead Contractor), Johns Hopkins University (Contributing Contractor), and Cincinnati Children's Hospital Medical Center (Contributing Contractor). Participants will be enrolled in the 2021-2022 and 2022-2023 influenza seasons. Individuals will be enrolled who have not received their influenza vaccine during the influenza season during which they are recruited and are either receiving a booster dose of mRNA COVID-19 vaccine (for ages booster is authorized and recommended) or are receiving a primary two-dose mRNA COVID-19 vaccine series (5 years of age or older). Future mRNA COVID vaccines may include SARS-CoV-2 viral variants which may be included in this study if authorized or approved by the FDA and recommended by the CDC. Health, demographic and health-related quality of life (HRQOL) data will be collected from study participants 12 years of age or older at baseline. All participants receiving an mRNA COVID-19 vaccine on Day 1 (Visit 1) will be randomized to receive quadrivalent inactivated influenza vaccine (IIV4) or placebo. Persons aged <65 years will receive standard dose IIV4 (SD-IIV4) or saline placebo injection. To qualify for the study, children < 9 years of age must only need to receive a single dose of IIV4 per ACIP recommendations for the 2022-2023 season. Persons aged ≥65 years will receive high-dose IIV4 (HD-IIV4) or saline placebo injection. Participants will receive the vaccines in an observer-blinded manner on the same day as they receive mRNA COVID-19 vaccine. Seven to fourteen days later (Visit 2) those who received IIV4 at Visit 1 will receive placebo and those who received placebo on at Visit 1 will receive IIV4 (SD-IIV4 or HD-IIV4, depending upon age). Participants will then receive a second mRNA COVID-19 vaccine (Visit 3), if applicable, and according to the recommended schedule for vaccination for the respective mRNA COVID-19 vaccine product used.

With Day 1 serving as the initial day of vaccination, participants will be followed through Day 7 (total 7 days) for solicited symptoms of reactogenicity as described in Section 5.4. HRQOL data will be collected during this time period for persons aged ≥ 12 years. Solicited reactogenicity events will also be monitored through Day 7 (total 7 days) following Visit 2 (all participants) and Visit 3 (only those participants receiving primary 2-dose COVID-19 vaccine series). Participants will also be followed for 7 days after each vaccination visit for unsolicited adverse events, through Day 120 for serious adverse events (SAEs) and through Day 120 for adverse events of special interest (AESI), as described in Section 5.4.

3.2 Laboratory Studies

3.2.1 Influenza Hemagglutination Inhibition Assay

mRNA COVID-19 vaccine naïve participants will have blood draws on Day 1 (before vaccination) and Day 21 (2021-2022 season) or Day 29 (2022-2023 season) (Pfizer/BioNTech mRNA vaccine) or Day 29 (Moderna mRNA post-IIV4 vaccination to be stored for serum hemagglutination inhibition (HAI) antibody titers. Those receiving a booster dose of mRNA COVID-19 vaccine will have blood draws on Day 1 (before vaccination) and Day 29. HAI antibody titers will be compared between groups receiving COVID-19 and IIV4 simultaneously or sequentially for each of the four influenza vaccine strains contained in the respective vaccines for that season. Participants will not receive individual HAI antibody titer results; these are not routinely used in clinical practice.

3.2.2 SARS-CoV-2 Antibody Assay

3.2.2.1 Qualitative baseline assessment

Participants will have blood draws on day 1 (before vaccination) as noted in 3.2.1
Serum will be assayed for the presence of SARS-CoV-2 antibody using the AdviseDx SARS-CoV-2 IgG II assay and the Alinity I SARS-CoV-2 IgG assay. The assays are intended for use as an aid in identifying individuals with an adaptive immune response to SARS-CoV-2 indicating recent or prior infection. Serologic testing will be completed in periodic batches throughout the course of the study and will therefore not be available in real-time or for use in clinical decision-making. The current tests described above are allowed under the FDA's Emergency Use Authorization during the COVID-19 pandemic. Duke will share participant results with each study site as permitted by FDA and CDC. Study sites may share results with participant as permitted by FDA, CDC, and local site regulations.

3.2.2.2 Quantitative SARS-CoV-2 assessments

Participants will have blood draws on Day 1 (before vaccination) and following each vaccination according to schedules outlined in Section 5.1 to be stored for serum COVID-19 neutralization assays. COVID-19 neutralizing antibody titers will be compared between groups receiving COVID-19 and IIV4 simultaneously or sequentially for each variant tested. Participants will not receive individual COVID-19 antibody titer results; these are not routinely used in clinical practice.

3.2.3 Future studies

In addition to the specified analyses described thus far, there may be other tests or assays that have yet to be identified that may be important for interpreting our study findings or of relevance to vaccine outcomes. Additional laboratory assays may test for antibodies against other bacteria or viruses, markers of inflammation, or used in research on the health of the participants. Specimens banked for use in other studies will be linked to information (including identifying information) that participants provided to the study. Subjects/subject's legally authorized representative (LAR) must agree to potential future use of samples in order to be in the study. Because it is unknown if future testing will be of any utility, results of future testing will not be provided.

4 STUDY ENROLLMENT AND WITHDRAWAL

4.1 Subject Inclusion Criteria

Subjects who meet all of the following criteria will be eligible to participate in this interventional study.

1. Persons aged ≥5 years if receiving primary two-dose mRNA COVID-19 vaccine series or persons aged ≥12 years if receiving a booster mRNA COVID-19 vaccine dose according to FDA authorization or approval and ACIP recommendation. Note: receipt of an mRNA COVID-19 vaccine within 8 hours of enrollment is permitted
   * Individuals age 5-11 receiving a booster may be enrolled in the event a booster for individuals age 5-11 is authorized or approved and recommended by the ACIP.
2. English or Spanish literate
3. Intention of receiving influenza vaccine and mRNA COVID-19 vaccine based on ACIP-CDC guidelines
4. Willing to provide written informed consent
5. Intention of being available for entire study period and complete all relevant study procedures, including follow-up phone calls and clinic visits

4.2 Subject Exclusion Criteria

Subjects who meet any of the following criteria will not be eligible to participate in this study:

1. Currently pregnant, planning to become pregnant within the first three months of the study per participant self-report or likely to be pregnant per screening criteria as defined in Section 5.1 at Visit 1
2. Prior receipt of IIV4 during the respective influenza season in which they are being enrolled
3. < 9 years of age and recommended to receive two doses of IIV4 during the respective influenza season in which they are being enrolled
4. Prior receipt of non-mRNA COVID-19 vaccine
5. Documented COVID-19 infection within 6 weeks prior to enrollment confirmed by either medical history or lab testing
6. History of severe allergic reaction after a previous dose of any influenza vaccine; or to an influenza vaccine component, including egg protein
7. History of severe adverse reaction associated with a vaccine and/or severe allergic reaction (e.g. anaphylaxis) to any component of an mRNA vaccine
8. Receipt of any licensed inactivated vaccine within 2 weeks prior to enrollment in this study, receipt of any licensed live vaccine within 4 weeks prior to enrollment in this study, or receipt of Shingrix (Zoster Vaccine Recombinant, Adjuvanted) or HEPLISAV-B (Hepatitis B Vaccine (Recombinant), Adjuvanted) vaccine within 6 weeks prior to enrollment in this study or planning receipt of any vaccines following enrollment until 6 weeks after receipt of the second dose of mRNA COVID-19 vaccine
9. Has an active neoplastic disease (excluding non-melanoma skin cancer or prostate cancer that is stable in the absence of therapy) or a history of any hematologic malignancy*
   **Participants with a history of malignancy may be included if, after previous treatment by surgical excision, chemotherapy or radiation therapy, the participant has been observed for a period that in the investigator's estimation provides a reasonable assurance of sustained cure*

10. Thrombocytopenia, bleeding disorder, or anticoagulant use contraindicating intramuscular injection (a daily aspirin may be acceptable).
11. Has immunosuppression as a result of an underlying illness or medications, such as antirejection/transplant regimens or immunomodulatory agents. Stable HIV disease is permitted per the following parameters:
    a. Confirmed stable HIV disease defined as document viral load <50 copies/mL and CD4 count >200 within 6 months before enrollment, and on stable antiretroviral therapy for at least 6 months
12. Has known hepatitis B (HBV) or hepatitis C (HBC). Stable HBV or HBC are permitted per the following parameters:
    a. If known HBV: confirmed inactive chronic HBV infection: HBsAg present for ≥6 months and HBeAg negative, anti-HBe positive; serum HBV DNA <2000 IU/mL; persistently normal ALT or AST levels; in those who had liver biopsy, findings that confirm absence of significant necroinflammation
    b. If known HCV: evidence of sustained virological response for ≥12 weeks after treatment or without evidence of HCV RNA viremia (undetectable HCV RNA)
13. Use of oral, parenteral, or high-dose inhaled glucocorticoids*
    **For definition of high-dose inhaled glucocorticoids, reference Appendix B.*
14. History of Guillain-Barré syndrome
15. Prior enrollment in this study during the 2021-22 flu season
16. Anyone who is already enrolled or plans to enroll in another clinical trial with an investigational product during the study period.*
    **Per protocol, co-enrollment in observational or behavioral intervention studies are permitted at any time. An investigational product may be permitted for therapy of an illness condition that occurs during the study period e.g. COVID-19 illness.*
17. Hearing loss determined by the investigators to prevent successful communication over the phone
18. History of myocarditis or pericarditis
19. History of multisystem inflammatory syndrome in children (MIS-C) or adults (MIS-A).
20. Has injury or other reason why deltoid site on both arms cannot be used for vaccinations.
21. Any condition which, in the opinion of the investigators, may pose a health risk to the subject or interfere with the evaluation of the study objectives.
22. Anyone who is a relative of any research study personnel.
23. Anyone who is an employee of any research study personnel.

4.3 Temporary Delay Criteria (Visit 1, 2 and 3 (for primary COVID-19 vaccine series))

1. History of febrile illness (> 100.0°F or 37.8°C) within the past 72 hours prior to vaccine administration

4.4 Recruitment

Potential participants ≥5 years of age will be recruited from several sources at Duke University Medical Center (DUMC), Johns Hopkins University (JHU), and Cincinnati using varying techniques. Study investigators will enroll at least 450 persons over two influenza seasons (2021-2022 and 2022-2023). Approximately 160 will be enrolled at Duke, 160 will be enrolled at Johns Hopkins, and 130 at Cincinnati Children's Hospital.

The general techniques for how subjects will be recruited include the following: Study staff, including PIs and clinical research coordinators will approach patients/patient's LAR(s) seen during clinic visits about the study; notify other health care professionals in their health system about the study via letters and flyers for potential referrals; notify potential subjects/ subject's LAR(s) about the study via study registries and recruitment service programs. More specific mechanisms to DUMC, JHU, and Cincinnati are described below. In addition, research staff will work with local COVID-19 vaccination clinics to recruit study participants.

At DUMC, we will plan to enroll persons for whom the COVID-19 vaccine is recommended by the ACIP. Duke will plan to work with Duke Employee Health and Wellness (OEHW) to identify employees that have not yet been vaccinated or may require a COVID-19 vaccine booster dose. Duke will then use that list of healthcare workers in an effort to assist with recruitment. In addition, Duke will partner with local COVID-19 vaccination clinics and other Duke clinics for potential participant referrals. For the proposed study, we will not enroll long-term care facility residents. These sources are likely to be sufficient, but if not, we will utilize other past successful strategies such as a letter campaign to households of older adults and advertising in senior citizen newspapers and general local newspapers.

At JHU, we will also enroll people for whom the COVID-19 vaccine is recommended by the ACIP. For community outreach, we partner with local broadcast, cable television, and OTT (over-the-top) providers to run 15 and 30 second commercials that are IRB-approved under our general screening protocol. In addition, the Center for Immunization Research (CIR) has its own website (https://centerforimmunizationresearch.org/) which includes a "Join A Study" page dedicated to recruitment for upcoming studies. The CIR has also partnered with local community organizations to increase awareness of and community involvement in clinical trials of coronavirus vaccines. This includes community organizations in the African American community as well as the LatinX community. We have Spanish-speaking staff who can enroll and consent subjects in Spanish.

The CIR maintains a recruitment tracking system that securely stores contact information for previous study participants and others who have previously contacted the Center. CIR staff may contact those people who have expressed permission to be contacted for future studies as an additional recruitment method. In addition to our dedicated recruitment phone line, people can contact us directly via our CIR Facebook, Twitter, and website to learn about our studies.

In order to recruit health care workers and other frontline workers, specifically targeted advertisements will be used in health care settings to recruit those workers. For the 2021-2022 season, in order to recruit healthcare workers, we may partner with Occupational health so that workers are informed of the study at the time of the annual influenza vaccination.

At Cincinnati Children's Hospital Medical Center (CCHMC), we will also enroll persons for whom the COVID-19 vaccine is recommended by the ACIP. We have been very successful in recruiting adults through email advertisements to our employee population. We have over 16,000 employees, and we have recruited successfully from this population and their friends and family members for many trials. We will also reach out to the public vaccination clinic at CCHMC, which is now vaccinating individuals ages 5 and over with COVID-19 vaccine. We may recruit pediatric participants with direct outreach to patients scheduled to receive a COVID-19 vaccine at our Pediatric Primary Care Clinic. In regard to older adults, we will continue to

collaborate with our local retirement communities to recruit and enroll older adults. We will not enroll long-term care facility residents for this study. We have successfully enrolled older adults for previous vaccine trials from our collaborating retirement community, Maple Knoll Village. We believe that these resources will be sufficient, but if not, we will utilize other past successful strategies like our vaccine trial participant database and advertisements in local medical offices, hospitals, and retirement communities.

At all sites, existing patients or their parent(s)/LAR(s) may be approached by phone call. The study will be reviewed with the patient or their parent(s)/LAR(s), and if the patient is interested, then initial eligibility screening will take place on the phone following an IRB-approved script. A waiver of documentation of consent will be approved from the reviewing IRB in order to carry out these screening activities.

4.5 Reasons for and Handling of Withdrawals

The following may be reason for study withdrawal:

- As deemed necessary by the principal investigator (PI).
- Subject or parent(s)/LAR(s) withdrawal of consent.
- Loss to follow-up.
- Subject unable to return for study appointments
- Termination of the study by the sponsor

Subjects or their parent(s)/LAR(s) may withdraw their consent for study participation at any time and for any reason, without penalty. Subjects who withdraw from the study prior to randomization will be replaced. Subjects who withdraw from the study after randomization will not be replaced. Data collected before withdrawal will still be used for analysis.

4.6 Termination of Study

This study may be terminated for safety concerns of the principal investigators from the Lead or Contributing sites, CDC, or participating Institutional Review Boards (IRBs).

5 STUDY SCHEDULE, PROCEDURES, & EVALUATIONS

5.1 Schedule of events and data collection

Persons meeting the proposed eligibility criteria (**Section 4**) will be recruited. Written informed consent will be obtained from study participants/participant's LAR(s) prior to conducting any study procedures. Prescreening may take place over the phone prior to Visit 1. **Tables 5-7** describe the schedule of study visits with further details below for the BNT162b2 and mRNA-1273 COVID-19 vaccines, respectively.

5.1.1 Primary Two-Dose COVID-19 Series Schedule of Events

Table 5: BNT162b2 (Pfizer-BioNTech) Schedule of Events

Procedure	Visit 1	Visit 1a	Visit 2	Visit 2a	Visit 3[1]	Visit 3a	Visit 3b	Visit 4[2]	Visit 5	Visit 6	Un-Scheduled
	Clinic	Phone/Text /Email/Data Review[3,4,5]	Clinic	Phone/Text /Email/Data Review[3,4,5]	Clinic	Phone/ Clinic	Phone/Text /Email/Data Review[3,4,5]	Clinic	Clinic	Phone	Clinic
Estimated Study Day (Relative to Visit 1)	**1**	**1-7**	**8-15**	**8-15(+6)**	**29**	**22-57**	**22-57(+6)**	**36-43**	**50-85**	**121**	
Time Following Visit 1 (Days) [Window]	0	0-6	7-14 [+2]		28 [+/-4]	21-56 [+/-4]				120 (+/-14)	
Time Following Visit 2 (Days) [Window]				0-6				28 [+/-4]			
Time Following Visit 3a (Days) [Window]							0-6		28 [+/-4]		
Informed consent & Medical Release of Information	X										
Review Eligibility Criteria	X										
Review Temporary Delay Criteria	X		X			X[6]					
Demographic and Health History	X										
Influenza and COVID-19 Vaccination History	X										
Obtain unsolicited adverse events			X		X	X		X			
Obtain SAE and AESI information			X		X	X		X	X	X	X
Concomitant medications	X		X		X	X		X	X	X	X
Additional COVID-19 vaccinations										X	
HRQOL measure[7]	X	X									
Measure temperature	X		X			X[6]					X
Venipuncture	X				X			X	X		
Randomization	X										
Vaccination with COVID-19 vaccine	X[8]					X[9,10]					

Table 5: BNT162b2 (Pfizer-BioNTech) Schedule of Events

Procedure	Visit 1	Visit 1a	Visit 2	Visit 2a	Visit 3[1]	Visit 3a	Visit 3b	Visit 4[2]	Visit 5	Visit 6	Un-Scheduled
	Clinic	Phone/Text /Email/Data Review[3,4,5]	Clinic	Phone/Text /Email/Data Review[3,4,5]	Clinic	Phone/ Clinic	Phone/Text /Email/Data Review[3,4,5]	Clinic	Clinic	Phone	Clinic
Estimated Study Day (Relative to Visit 1)	**1**	**1-7**	**8-15**	**8-15(+6)**	**29**	**22-57**	**22-57(+6)**	**36-43**	**50-85**	**121**	
Time Following Visit 1 (Days) [Window]	0	0-6	7-14 [+2]		28 [+/-4]	21-56 [+/-4]				120 (+/-14)	
Time Following Visit 2 (Days) [Window]				0-6				28 [+/-4]			
Time Following Visit 3a (Days) [Window]							0-6		28 [+/-4]		
Vaccination with IIV4 or Placebo	X		X								
Memory Aid, Link to electronic symptom diary given to participant	X		X			X					
Assess for any immediate reactogenicity symptoms	X		X			X					
Electronic Diary Review[3]		X		X			X				
Complete electronic or paper symptom diary[1]	X	X	X	X		X	X				

1. *Note that Visit 3 is timed relative to Visit 1 but may be conducted on the same day as Visit 3a if Visit 3a occurs 24-28 days after Visit 1. Visit 3 and Visit 4 should not occur on the same day.*
2. *Note that Visit 4 is timed relative to Visit 2 and may be conducted on the same day as Visit 3a if Visit 3a occurs 24-28 days after Visit 2. Visit 3 and Visit 4 should not occur on the same day.*
3. *Symptom diary (solicited local and systemic reactogenicity events) to be completed by participant on Days 1-7 after vaccination.*
4. *Participants completing paper diary only will be called 2-4 days following Visits 1, 2, and 3 as a reminder and to prompt to bring paper diary to next visit*
5. *Electronic diary records will be reviewed by study staff at days 2-4 days and 8-10 days following Visit 1, 2 and 3.*
6. *If Visit 3a is conducted by telephone as standard of care these procedures do not need to be performed*
7. *HRQOL will only be collected for participants 12 years of age and older.*
8. *Receipt of BNT162b2 within 8 hours of enrollment is permitted*
9. *Receipt of BNT162b2 within 36 hours prior to study visit is permitted*
10. *Extended interval beyond 25 days not recommended for persons < 12 years of age*

Table 6: mRNA-1273 (Moderna) Schedule of Events

Procedure	Visit 1	Visit 1a	Visit 2	Visit 2a	Visit 3[1]	Visit 3a	Visit 3b	Visit 4[2]	Visit 5	Visit 6	Un-Scheduled
	Clinic	Phone/Text /Email/Data Review[3,4,5]	Clinic	Phone/Text /Email/Data Review[3,4,5]	Clinic	Phone/ Clinic	Phone/Text /Email/Data Review[3,4,5]l	Clinic	Clinic	Phone	Clinic
Estimated Study Day (Relative to Visit 1)	**1**	**1-7**	**8-15**	**8-15(+6)**	**29**	**29-57**	**29-57(+6)**	**36-43**	**57-85**	**121**	
Time Following Visit 1 (Days) [Window]	0	0-6	7-14 [+2]		28 [+/-4]	28-56 [+/-4]				120 (+/-14)	
Time Following Visit 2 (Days) [Window]				0-6				28 [+/-4]			
Time Following Visit 3a (Days) [Window]							0-6		28 [+/-4]		
Informed consent & Medical Release of Information	X										
Review Eligibility Criteria	X										
Review Temporary Delay Criteria	X		X			X[6]					
Demographic and Health History	X										
Influenza and COVID-19 Vaccination History	X										
Obtain unsolicited adverse events			X		X	X		X			
Obtain SAE and AESI information			X		X	X		X	X	X	X
Concomitant medications	X		X		X	X		X	X	X	X
Additional COVID-19 vaccinations										X	
HRQOL measure[7]	X	X									
Measure temperature	X		X			X[6]					X
Venipuncture	X				X[4]			X[4]	X		
Randomization	X										
Vaccination with COVID-19 vaccine	X[8]					X[9,10]					
Vaccination with IIV4 or Placebo	X		X								

Table 6: mRNA-1273 (Moderna) Schedule of Events

Procedure	Visit 1	Visit 1a	Visit 2	Visit 2a	Visit 3[1]	Visit 3a	Visit 3b	Visit 4[2]	Visit 5	Visit 6	Un-Scheduled
	Clinic	Phone/Text /Email/Data Review[3,4,5]	Clinic	Phone/Text /Email/Data Review[3,4,5]	Clinic	Phone/ Clinic	Phone/Text /Email/Data Review[3,4,5]l	Clinic	Clinic	Phone	Clinic
Estimated Study Day (Relative to Visit 1)	**1**	**1-7**	**8-15**	**8-15(+6)**	**29**	**29-57**	**29-57(+6)**	**36-43**	**57-85**	**121**	
Time Following Visit 1 (Days) [Window]	0	0-6	7-14 [+2]		28 [+/-4]	28-56 [+/-4]				120 (+/-14)	
Time Following Visit 2 (Days) [Window]				0-6				28 [+/-4]			
Time Following Visit 3a (Days) [Window]							0-6		28 [+/-4]		
Memory Aid, Link to electronic symptom diary given to participant	X		X			X					
Assess for any immediate reactogenicity symptoms	X		X			X					
Electronic Diary Review[2]		X		X			X				
Complete electronic symptom diary[1]	X	X	X	X		X	X				

1. *Note that Visit 3 is timed relative to Visit 1 but may be conducted on the same day as Visit 3a if Visit 3a occurs 24-32 days after Visit 1. Visit 3 and Visit 4 should not occur on the same day.*
2. *Note that Visit 4 is timed relative to Visit 2 and may be conducted on the same day as Visit 3a if Visit 3a occurs 24-32 days after Visit 2. Visit 3 and Visit 4 should not occur on the same day.*
3. *Symptom diary (solicited local and systemic reactogenicity events) to be completed by participant on Days 1-7 after vaccination.*
4. *Participants completing paper diary only will be called 2-4 days following Visits 1, 2, and 3 as a reminder and to prompt to bring paper diary to next visit*
5. *Electronic diary records will be reviewed by study staff at days 2-4 days and 8-10 days following Visit 1, 2 and 3.*
6. *If Visit 3a is conducted by telephone as standard of care these procedures do not need to be performed*
7. *HRQOL will only be collected for participants 12 years of age and older.*
8. *Receipt of BNT162b2 within 8 hours of enrollment is permitted*
9. *Receipt of BNT162b2 within 36 hours prior to study visit is permitted*
10. *Extended interval beyond 32 days not recommended for persons < 12 years of age*

Visit 1, Study Day 1 - Screening, Enrollment, and Vaccination (Clinic Visit)

- Obtain written informed consent and release of medical record information
- Obtain information on preferred method of contact for follow-up (telephone, text or email)
- Review and confirm study eligibility and temporary delay criteria including pregnancy status and pregnancy intention for women of childbearing potential.
  For women of childbearing potential only, the study team can assess likelihood of pregnancy based upon the following criteria if there are concerns regarding pregnancy. If yes to any, can be reasonably certain not pregnant if no symptoms or signs of pregnancy.
    - Is ≤ 7 days after the start of menses
    - Has not had sexual intercourse since the start of last menses
    - Has been consistently using a reliable method of contraception
    - Is ≤ 7 days after spontaneous or induced abortion
    - Is within 4 weeks postpartum
    - Is fully or nearly fully breastfeeding (exclusively breastfeeding or the vast majority [≥ 85%] of feeds are breastfeeds), amenorrheic and < 6 months postpartum
- Obtain demographic and medical history including history of influenza and COVID-19 vaccination and reported PCR test positive COVID-19 infection
- Obtain concomitant medication use in preceding 30 days
- Obtain baseline HRQOL assessment (for participants 12 years of age and older)
- Obtain oral temperature
- Obtain 10mL blood sample for those ≥ 12 year of age and 5mL blood sample for those 5-11 years of age prior to vaccination for serologic analysis (**Section 5.6.1**)
- Randomize study participant to observer-blinded IIV4 or placebo (**Section 5.6.1**)
- Participant will either receive or have just received (within 8 hours) mRNA COVID-19 vaccine per local standard of care or mRNA COVID-19 vaccine will be administered in the deltoid muscle by trained, licensed research staff.
- Trained, licensed unblinded staff will administer study product (IIV4 or placebo) in the opposite deltoid muscle in which mRNA COVID-19 vaccine was administered
- Ensure participants/participant's LAR(s) receive the IIV4 Vaccine Information Sheet (VIS)[39] and/or the respective fact sheet for recipients and caregivers for mRNA COVID-19 vaccine during visit[13,43].
- Dispense paper memory aid (if subject selects paper), thermometer, and ruler (in order to standardize measurements). Review instructions for use of thermometer, ruler, and electronic memory aid completion.
- Observe participant for a minimum of 30 minutes and assess for any immediate reactogenicity symptoms
- Confirm date of next appointment

Visit 1a, Study Days 1 – 7 Post Visit 1

- Participants/participant's LAR(s) complete electronic or paper solicited symptom diary and HRQOL assessment at approximately the same time each day for days 1-7
- Participants/participant's LAR(s) using paper diary:

    - Study staff will call participants/participant's LAR(s) on 2-4 days following Visit 1 as a reminder to fill out the symptom diary and HRQOL assessments on day 1-7 post Visit 1 regarding reactogenicity events and to bring the diary card to the next visit.
    - Participants/participant's LAR(s) will be:
        - Asked to notify the study staff if they or their child is hospitalized or have a severe adverse event
        - Follow up with their or their child's healthcare provider if they have symptoms they find concerning
- Participants/participant's LAR(s) using REDCap web-based system:
    - Study staff will send daily reminders to fill out the symptom diary and HRQOL
    - Study staff will review REDCap system to confirm data capture and assess for any AE or SAEs 2-4 days and 8-10 days following Visit 1.
    - The study team will contact participants/participant's LAR(s) if they have any missing information. The study team may also contact participants if more information is needed to better describe AEs [including SAEs and severe (Grade 3) events] reported in the REDCap web-based system.
    - All participants/participant's LAR(s) using the REDCAP web-based system will be:
        - Asked to notify the study staff if they or their child is hospitalized or have a severe adverse event
        - Follow up with their or their child's healthcare provider if they have symptoms they find concerning

Visit 2, Study Day 8-15 Clinic Visit

- Review any temporary delay criteria
- Record any unsolicited AEs within the first 7 days post-vaccination, SAEs, AESI
- Obtain concomitant medication use
- Obtain oral temperature
- Trained, licensed unblinded staff will administer IIV4 or placebo in the deltoid muscle as described in **Section 5.3.1**.
- Ensure participants/participant's LAR(s) receive the IIV4 Vaccine Information Sheet (VIS)
- Ensure participants/participant's LAR(s) receive IIV4 Vaccine Verification
- Dispense paper memory aid, thermometer if needed, and ruler if needed (in order to standardize measurements). Review instructions for use of thermometer, ruler, and electronic memory aid completion.
- Observe participant for a minimum of 30 minutes and assess for any immediate reactogenicity symptoms
- Confirm date of next appointment

Visit 2a, Study Days 1 – 7 Post Visit 2

- Participants/participant's LAR(s) complete electronic or paper solicited symptom diary each day at approximately the same time each day for days 1-7
- Participants/participant's LAR(s) using paper diary:
    - Study staff will call participants/participant's LAR(s) on days 2-4 following Visit

2 as a reminder to fill out the symptom diary on day 1-7 post Visit 2 regarding reactogenicity events and to bring the diary card to the next visit.
  - Participants/participant's LAR(s) will be:
    - Asked to notify the study staff if they or their child is hospitalized or have a severe adverse event
    - Follow up with their or their child's healthcare if they have symptoms they find concerning
- Participants/participant's LAR(s) using REDCap web-based system:
  - Study staff will send reminders to fill out the symptom diary on day 1-7 post Visit 2 regarding reactogenicity events.
  - Study staff will review REDCap system to confirm data capture and assess for any AE or SAEs 2-4 days and 8-10 days following Visit 2
  - The study team will contact participants/participant's LAR(s) if they have any missing information. The study team may also contact participants/participant's LAR(s) if more information is needed to better describe AEs [including SAEs and severe (Grade 3) events] reported in the REDCap web-based system.
  - All participants/participant's LAR(s) using the REDCAP web-based system will be:
    - Asked to notify the study staff if they or their child is hospitalized or have a severe adverse event
    - Follow up with their or their child's healthcare provider if they have symptoms they find concerning

Visit 3 Study Day 29 (BNT162b2 or mRNA-1273) Clinic Visit

- Record any unsolicited AEs within the first 7 days post-vaccination, SAEs, AESI
- Obtain concomitant medication use
- Obtain 10mL blood sample for those ≥ 12 year of age and 5mL blood sample for those 5-11 years of age prior to vaccination for serologic analysis (**Section 5.6.1**)
- Confirm date of next appointment

Visit 3a, Study Day 22-57 (BNT162b2) or 29-57 (mRNA-1273) Phone/Clinic Visit

- Review and confirm study eligibility and temporary delay criteria
- Record any unsolicited AEs within the first 7 days post-vaccination, SAEs, AESI
- Obtain concomitant medication use
- Obtain oral temperature
- Participant will either receive or have just received (within 36 hours) mRNA COVID-19 vaccine per local standard of care or mRNA COVID-19 vaccine will be administered in the deltoid muscle by trained, licensed research staff.as described in Section 5.3.1.
- Ensure participants/participant's LAR(s) receive the respective fact sheet for mRNA COVID-19 vaccine during visit.
- Dispense paper memory aid, thermometer if needed, and ruler if needed (in order to standardize measurements). Review instructions for use of thermometer, ruler, and electronic memory aid completion.
- Observe participant for a minimum of 30 minutes (if mRNA COVID-19 vaccine is given at the study visit) and assess for any immediate reactogenicity symptoms
- Confirm date of next appointment

Visit 3b, Study Days 1 – 7 Post Visit 3

- Participants/participant's LAR(s) complete electronic or paper solicited symptom diary each day at approximately the same time each day for days 1-7
- Participants/participant's LAR(s) using paper diary:
  - Study staff will call participants/participant's LAR(s) on days 2-4 following Visit 3 as a reminder to fill out the symptom diary on day 1-7 post Visit 3 regarding reactogenicity events and to bring the diary card to the next visit
  - Participants/participant's LAR(s) will be:
    - Asked to notify the study staff if they or their child is hospitalized or have a severe adverse event
    - Follow up with their or their child's healthcare provider if they have symptoms they find concerning
- Participants/participant's LAR(s) using REDCap web-based system:
  - Study staff will send reminders to fill out the symptom diary on day 1-7 post Visit 3 regarding reactogenicity events.
  - Study staff will review REDCap system to confirm data capture and assess for any AE or SAEs on 2-4 days and 8-10 days following Visit 3
  - The study team will contact participants/participant's LAR(s) if they have any missing information. The study team may also contact participants/participant's LAR(s) if more information is needed to better describe AEs [including SAEs and severe (Grade 3) events] reported in the REDCap web-based system.
  - All participants/participant's LAR(s) using the REDCAP web-based system will be:
    - Asked to notify the study staff if they or their child is hospitalized or have a severe adverse event
    - Follow up with their or their child's healthcare provider if they have symptoms they find concerning

Visit 4, Study Day 36-43 (BNT162b2 or mRNA-1273 vaccine) Clinic Visit

- Record any unsolicited AEs within the first 7 days post-vaccination, SAEs, AESI
- Obtain concomitant medication use
- Obtain 10mL blood sample for those ≥ 12 year of age and 5mL blood sample for those 5-11 years of age prior to vaccination for serologic analysis (**Section 5.6.1**)
- Confirm date of next appointment

Visit 5, Study Day 50-85 (BNT162b2) or 57-85 (MRNA-1273) Clinic Visit

- Record any SAEs and AESI
- Obtain concomitant medication use
- Obtain 10mL blood sample for those ≥ 12 year of age and 5mL blood sample for those 5-11 years of age prior to vaccination for serologic analysis (**Section 5.6.1**)

Visit 6, Study Day 121 Phone Visit

- Record any additional COVID-19 vaccinations since Visit 3a
- Record any SAEs and AESI
- Obtain concomitant medication use

Unscheduled Visits

- Obtain vital signs including oral temperature
- Review any solicited (≤ day 7) AEs submitted electronically, unsolicited AEs, SAEs, AESI, and concomitant medications (throughout period of enrollment)

5.1.2 Booster Dose mRNA COVID-19 Vaccine Schedule of Events

Table 7: Booster Dose Schedule of Events

**Procedure**	**Visit 1**	**Visit 1a**	**Visit 2**	**Visit 2a**	**Visit 3**	**Visit 4**	**Visit 5**	**Un-Scheduled**
	Clinic	Phone/Text Email/Data Review[1,2,3]	Clinic	Phone/Text Email/Data Review[1,2,3]	Clinic	Clinic	Phone	Clinic
Estimated Study Day (Relative to Visit 1)	**1**	**1-7**	**8-15**	**8-21**	**29**	**36-43**	**121**	
Time Following Visit 1 (Days) [Window]	0	0-6	7-14 [+2]		28 [+/-4]		120 (+/-14)	
Time Following Visit 2 (Days) [Window]				0-6		28 [+/-4]		
Informed consent & Medical Release of Information	X							
Review Eligibility Criteria	X							
Review Temporary Delay Criteria	X		X					
Demographic and Health History	X							
Influenza and COVID-19 Vaccination History	X							
Obtain unsolicited adverse events			X		X	X		
Obtain SAE and AESI information			X		X	X	X	X
Concomitant medications	X		X		X	X	X	X
Additional COVID-19 vaccinations							X	
HRQOL measure[4]	X	X						
Measure temperature	X		X					X
Venipuncture	X				X	X		
Randomization	X							
Vaccination with COVID-19 vaccine	X[5]							
Vaccination with IIV4 or Placebo	X		X					
Memory Aid, Link to electronic symptom diary given to participant	X		X					
Assess for any immediate reactogenicity symptoms	X		X					
Electronic Diary Review[2]		X		X				

Table 7: Booster Dose Schedule of Events

Procedure	Visit 1	Visit 1a	Visit 2	Visit 2a	Visit 3	Visit 4	Visit 5	Un-Scheduled
	Clinic	Phone/Text Email/Data Review[1,2,3]	Clinic	Phone/Text Email/Data Review[1,2,3]	Clinic	Clinic	Phone	Clinic
Estimated Study Day (Relative to Visit 1)	**1**	**1-7**	**8-15**	**8-21**	**29**	**36-43**	**121**	
Time Following Visit 1 (Days) [Window]	0	0-6	7-14 [+2]		28 [+/-4]		120 (+/-14)	
Time Following Visit 2 (Days) [Window]				0-6		28 [+/-4]		
Complete electronic symptom diary[1]	X	X	X	X				

1 Symptom diary (solicited local and systemic reactogenicity events) to be completed by participant on Days 1-7 after vaccination.
2 Participants completing paper diary only will be called at days 3 [+2] and 17[+2] as a reminder and to prompt to bring paper diary to next visit
3 Electronic diary records will be reviewed by study staff at day 3[+2], 8[+2], 17[+2], and 22[+2] for completion or issues.
4 HRQOL will only be collected for participant 12 years of age and older.
5 Receipt of mRNA-1273 or BNT162b2 within 8 hours of enrollment is permitted

Visit 1, Study Day 1 - Screening, Enrollment, and Vaccination (Clinic Visit)

- Obtain written informed consent and release of medical record information
- Obtain information on preferred method of contact for follow-up (telephone or email)
- Review and confirm study eligibility and temporary delay criteria including pregnancy status and pregnancy intention for women of childbearing potential.

  For women of childbearing potential only, the study team can assess likelihood of pregnancy based upon the following criteria if there are concerns regarding pregnancy. If yes to any, can be reasonably certain not pregnant if no symptoms or signs of pregnancy.
    - Is ≤ 7 days after the start of menses
    - Has not had sexual intercourse since the start of last menses
    - Has been consistently using a reliable method of contraception
    - Is ≤ 7 days after spontaneous or induced abortion
    - Is within 4 weeks postpartum
    - Is fully or nearly fully breastfeeding (exclusively breastfeeding or the vast majority [≥ 85%] of feeds are breastfeeds), amenorrheic and < 6 months postpartum
- Obtain demographic and medical history including history of influenza and COVID-19 vaccination and reported PCR test positive COVID-19 infection
- Obtain concomitant medication use in preceding 30 days
- Obtain baseline HRQOL assessment (for participants 12 years of age and older)
- Obtain oral temperature
- Obtain 10mL blood sample for those ≥ 12 year of age and 5mL blood sample for those 5-11 years of age prior to vaccination for serologic analysis (**Section 5.6.1**)
- Randomize study participant to observer-blinded IIV4 or placebo (**Section 5.6.1**)
- Participant will either receive or have just received (within 8 hours) mRNA COVID-19 vaccine per local standard of care or mRNA COVID-19 vaccine will be administered in

the deltoid muscle by trained, licensed research staff.
- Trained, licensed unblinded staff will administer study product (IIV4 or placebo) in the opposite deltoid muscle in which mRNA COVID-19 vaccine was administered
- Ensure participants/participant's LAR(s) receive the IIV4 Vaccine Information Sheet (VIS)[39] and/or the respective fact sheet for recipients and caregivers for mRNA COVID-19 vaccine during visit[13,43].
- Dispense paper memory aid (if subject selects paper), thermometer, and ruler (in order to standardize measurements). Review instructions for use of thermometer, ruler, and electronic memory aid completion.
- Observe participant for a minimum of 30 minutes and assess for any immediate reactogenicity symptoms
- Confirm date of next appointment

Visit 1a, Study Days 1 – 7 Post Visit 1
- Participants/participant's LAR(s) complete electronic or paper solicited symptom diary and HRQOL assessment at approximately the same time each day for days 1-7
- Participants/participant's LAR(s) using paper diary:
    - Study staff will call participants/participant's LAR(s) on days 2-4 following Visit 1 as a reminder to fill out the symptom diary and HRQOL assessments on day 1-7 post Visit 1 regarding reactogenicity events and to bring the diary card to the next visit.
    - Participants/participant's LAR(s) will be:
        - Asked to notify the study staff if they or their child is hospitalized or have a severe adverse event
        - Follow up with their or their child's healthcare provider if they have symptoms they find concerning
- Participants/participant's LAR(s) using REDCap web-based system:
    - Study staff will send daily reminders to fill out the symptom diary and HRQOL
    - Study staff will review REDCap system to confirm data capture and assess for any AE or SAEs on 2-4 days and 8-10 days following Visit 1.
    - The study team will contact participants/participant's LAR(s) if they have any missing information. The study team may also contact participants/participant's LAR(s) if more information is needed to better describe AEs [including SAEs and severe (Grade 3) events] reported in the REDCap web-based system.
    - All participants/participant's LAR(s) using the REDCAP web-based system will be:
        - Asked to notify the study staff if they or their child is hospitalized or have a severe adverse event
        - Follow up with their or their child's healthcare provider if they have symptoms they find concerning

Visit 2, Study Day 8 - 15 Clinic Visit
- Review any temporary delay criteria
- Record any unsolicited AEs within the first 7 days post-vaccination, SAEs, AESI
- Obtain concomitant medication use
- Obtain oral temperature

- Trained, licensed unblinded staff will administer IIV4 or placebo in the deltoid muscle as described in **Section 5.3.1**.
- Ensure participants/participant's LAR(s) receive the IIV4 Vaccine Information Sheet (VIS)
- Ensure participants/participant's LAR(s) receive IIV4 Vaccine Verification
- Dispense paper memory aid, thermometer if needed, and ruler if needed (in order to standardize measurements). Review instructions for use of thermometer, ruler, and electronic memory aid completion.
- Observe participant for a minimum of 30 minutes and assess for any immediate reactogenicity symptoms
- Confirm date of next appointment

Visit 2a, Study Days 1 – 7 Post Visit 2

- Participants/participant's LAR(s) complete electronic or paper solicited symptom diary each day at approximately the same time each day for days 1-7 post Visit 2
- Participants/participant's LAR(s) using paper diary:
  - Study staff will call participants/participant's LAR(s) on day 3 post Visit 2 as a reminder to fill out the symptom diary on day 1-7 post Visit 2 regarding reactogenicity events and to bring the diary card to the next visit.
  - Participants/participant's LAR(s) will be:
    - Asked to notify the study staff if they or their child is hospitalized or have a severe adverse event
    - Follow up with their or their child's healthcare if they have symptoms they find concerning
- Participants/participant's LAR(s) using REDCap web-based system:
  - Study staff will send reminders to fill out the symptom diary on day 1-7 post Visit 2 regarding reactogenicity events.
  - Study staff will review REDCap system to confirm data capture and assess for any AE or SAEs on 2-4 days and 8-10 days following Visit 2.
  - The study team will contact participants/participant's LAR(s) if they have any missing information. The study team may also contact participants/participant's LAR(s) if more information is needed to better describe AEs [including SAEs and severe (Grade 3) events] reported in the REDCap web-based system.
  - All participants/participant's LAR(s) using the REDCAP web-based system will be:
    - Asked to notify the study staff if they or their child is hospitalized or have a severe adverse event
    - Follow up with their or their child's healthcare provider if they have symptoms they find concerning

Visit 3, Study Day 29 Clinic Visit

- Record any unsolicited AEs, SAEs, AESI
- Obtain concomitant medication use
- Obtain 10mL blood sample for those ≥ 12 year of age and 5mL blood sample for those 5-11 years of age prior to vaccination for serologic analysis (**Section 5.6.1**)
- Confirm date of next appointment

Visit 4, Study Day 36 - 43 Clinic Visit

- Record any unsolicited AEs, SAEs, AESI
- Obtain concomitant medication use
- Obtain 10mL blood sample for those ≥ 12 year of age and 5mL blood sample for those 5-11 years of age prior to vaccination for serologic analysis (**Section 5.6.1**)

Visit 5, Study Day 121 Phone Visit

- Record any additional COVID-19 vaccinations since Visit 1
- Record any SAEs and AESI
- Obtain concomitant medication use

Unscheduled Visits

- Obtain vital signs including oral temperature
- Review any solicited (≤ day 7) AEs submitted electronically, unsolicited AEs, SAEs, AESI, and concomitant medications (throughout period of enrollment)

5.2 Treatment Assignment Procedures

This study is a prospective, randomized, observer-blinded, clinical trial involving subjects aged ≥5 years of age who are to receive COVID-19 and IIV4 vaccines.

5.2.1 Randomization

Participants will be randomized (1:1) to receive either IIV4 or saline by an unblinded vaccinator at Visit 1. Those receiving IIV4 at visit 1 will receive saline placebo at Visit 2 and those receiving saline placebo at Visit 1 will receive IIV4 at Visit 2. Randomization will be stratified by age (5 to < 12 years of age, 12 to < 18 years of age, 18 to <65 years of age, and ≥ 65 years of age) and receipt of either a primary two-dose series or any booster dose of mRNA COVID-19 vaccine (for ages booster is authorized and recommended) using a permuted block randomization scheme stratified by Lead and Contributing Sites. The project statistician will generate permuted block randomization schemes which will be uploaded to REDCap. The randomization schedule will not be available to the study staff, so the next randomization allocation will not be known before randomization occurs. Following confirmation of study eligibility criteria during Visit 1, participant randomization will be through REDCap with treatment allocation recorded on the unblinded vaccinator CRF. In the event that REDCap is unavailable, manual randomization will occur through the use of envelopes. The project statistician will prepare 10 envelopes per age group and vaccine series per site (total of 60 per site) that will use the same randomization strategy as the primary scheme embedded in REDCap. When an unblinded team member is informed of the age group, he/she will pull the next envelope in order. In order to capture the allocation per subject, a separate form in REDCap will be used by the unblinded personnel to add the assignment. A log will need to be kept at the site capturing these instances.

5.3 Data Collection

5.3.1 Study Product Supply, Storage, and Administration

In order to ensure adherence to study randomization assignment, age-appropriate, licensed IIV4 or saline placebo will be administered as a study procedure. EUA or approved mRNA COVID-19 vaccine will be administered either per standard of care or as a study procedure depending

on the study site and location. Licensed SD-IIV4 and HD-IIV4 vaccine (prefilled syringes) will be purchased for study administration and maintained at the study sites and stored at 2°C to 8°C in a research-specific medication refrigerator. Licensed or EUA COVID-19 vaccine will be stored per manufacturer specifications. While research staff maintain daily temperature logs for the medication storage, it is also monitored 24/7 with alarm activation if out of range. Research staff are notified of any alarm activations and have an on-call system in place to report to the research center for further investigation. Any potentially compromised vaccine will be quarantined for further disposition based on site-specific SOPs and investigator assessment.

A licensed unadjuvanted standard dose egg-based IIV4 will be the designated study product for influenza vaccination of persons less than 65 years of age[44]. Study sites will work to coordinate the brand of IIV4 administered. A single administration of IIV4 comprises intramuscular delivery of 0.5mL total volume of the vaccine for persons 5 through 64 years of age.

Fluzone High-Dose (Sanofi) will be the designated study products for influenza vaccination of persons 65 years of age and older. A single administration of HD-IIV4 comprises intramuscular delivery of 0.7mL total volume of the vaccine for adults 65 years of age and older.

Sterile normal saline supplied by each site's respective pharmacy will be used as placebo. A single administration of placebo comprises intramuscular deliver of 0.5 mL total volume of normal saline for persons 5 through 64 years of age and 0.7 mL total volume for those 65 years of age and older.

mRNA COVID-19 vaccine will be administered per FDA and ACIP recommendations for administration. The primary two-doses mRNA COVID-19 vaccine series will be administered in two doses, approximately 21 to 56 days or 28 to 56 days apart depending upon the FDA or CDC recommendations. The booster dose of mRNA COVID-19 vaccine will be administered according to FDA authorization or EUA and ACIP recommendation.

Vaccine lot numbers, dose, and site of vaccine administration will be recorded by research staff. After administration, used study syringes will be disposed of according to standard operating procedure.

Emergency management supplies including epinephrine (1:1000) and trained staff will be available for initial treatment of an allergic reaction, if needed.

5.3.2 Blinding

SD-IIV4, HD-IIV4, and/or placebo will be administered to blinded participants in the opposite deltoid muscle, to which the mRNA COVID-19 vaccine was administered at Visit 1. SD-IIV4 or HD-IIV4 will be administered by unblinded licensed staff. In order to keep the participant blinded, the vaccine administrator will keep the prefilled syringes out of view of the participant at all times and will instruct the participant to turn their head in the opposite direction of the arm in which the vaccine is being administered. Similar instructions will be given to any persons accompanying the patient in the room. After administration, used study syringes will be disposed of according to site-specific SOPs. A licensed provider (MD, DO, NP, PA, RN, LPN), who will be trained on treating adverse reactions, will be immediately available at the time of vaccine administration along with emergency management supplies available for initial treatment of an allergic reaction if needed. Additionally, clinical members of the blinded data collection team will be present to assist study subjects.

5.3.3 Unblinding

A copy of participant treatment assignments will be retained at the site in a REDCap database file accessible only to the unblinded site staff. In the event of an occurrence when knowledge of the treatment assignment will influence subject's care, the Principal Investigator may be provided with the treatment assignment. The Principal Investigator must contact the Lead Site, Duke and document the event with information regarding the reasons for unblinding. After discussion with the Lead site, the Principal Investigator may receive the treatment assignment from the Lead site or if necessary, from the on-site secure file by the unblinded staff. Unblinding events should be reported to the CDC.

All subjects will be unblinded at the end of study and informed of whether they received the influenza and COVID vaccines either sequentially or simultaneously.

5.3.4 Incorrect Dose Administration

In the event that a participant who is ≥ 65 years of age inadvertently receives standard-dose influenza vaccine rather than high-dose influenza vaccine, using shared decision making, they will be offered the opportunity to receive the preferred high-dose influenza vaccine a minimum of 28 days following the receipt of the standard-dose vaccine. Although there is no current recommendation to follow receipt of a standard-dose vaccine by a high-dose vaccine, participants will be offered high-dose vaccine to assure they achieve optimal protection from infection. A minimum interval of 28 days was chosen based on the routine practice of administering two separate standard-doses influenza vaccine 28 days apart in children who are influenza vaccine naïve. Participants choosing to receive a subsequent high-dose influenza vaccine will be called on Days 3 and 8 following receipt of their vaccine to review for the occurrence of any unsolicited adverse events. They will continue to be followed for the occurrence of AESIs and SAES as outlined in Section 5.4 below.

5.4 Reactogenicity and Safety Assessment

Participants will be assessed for any immediate reactogenicity in a period at least 30 minutes after vaccine administration while at the study site. They will be assessed for the following potential systemic reactions such as anaphylaxis related symptoms (e.g., dyspnea, chest tightness, wheezing, cough, stridor, urticarial, flushing, nasal congestion, dizziness, syncope, diaphoresis, emesis) or other medical symptoms (e.g., feverishness, chills/shivering, fatigue, myalgia, arthralgia, headache, nausea/vomiting, diarrhea). They will be assessed for any potential injection site reactions including pain, tenderness, erythema or induration.

Frequency and occurrence of solicited local and systemic reactogenicity will be assessed daily through post-vaccination Day 7 following Visits 1, 2 and 3 (if primary two-dose COVID-19 vaccine series) using a standard memory aid. Participants/participant's LAR(s) will submit this information electronically on a daily basis. For participants unable to submit electronically, paper memory aid completion and collection will be acceptable but not preferred. Injection site and systemic solicited events will be graded as noted in **Tables 8 and 9** [45]. Participants/participant's LAR(s) will be further queried upon presence of symptoms. Unsolicited adverse events (AEs) will be assessed during the first 7 days following each vaccination visit and concomitant medications will be assessed from 30 days prior to Visit 1 through the study period. Serious AEs (SAEs) and new onset of adverse events special interest (AESI) will be assessed through the entire period of a subject's participation (see below). Unsolicited AEs and SAEs will also be graded as to whether they are noticeable but do not interfere with activity (Grade 1), interfere

with some activity but did not need a medical visit or absenteeism (Grade 2), or prevent daily activity and/or resulted in medical visit and/or absenteeism (grade 3) or require an emergency room visit or hospitalization (Grade 4).

At the time of study enrollment, participants/participant's LAR(s) will be given a thermometer and instructed on using the symptom diary to document oral temperatures and post-injection symptoms. Beginning on the evening of Study Visits 1, 2 and 3 (if primary two-dose COVID-19 vaccine series) (Day 1) following vaccination, participants/participant's LAR(s) will record their or their child's oral temperature using the study-supplied thermometer, the occurrence of solicited AEs, for the next 7 days (Day 1 – 7). Temperature will be recorded at roughly the same time each day. If a temperature ≥ 100.0°F (37.8°C) is recorded, a second measurement will be taken. If more than one temperature is taken on the same day, the highest temperature should be recorded. Fever will be defined as a measured temperature ≥ 100.4°F (38.0°C). Participants/participant's LAR(s) will be queried during Visits 2, 3, and 4 on any solicited adverse events following vaccination. Injection site reactogenicity will be assessed separately for COVID-19 vaccine and IIV4 or placebo as described in **Table 8**.

Table 8. Injection-site Reactogenicity Grading

Local Reaction to Injectable Product	Mild (Grade 1)	Moderate (Grade 2)	Severe (Grade 3)	Potentially Life Threatening (Grade 4)
**Pain**	Noticeable but does not interfere with activity	Interferes with activity but did not need a medical visit or absenteeism [i.e. missing work or school]	Significant; prevents daily activity and/or resulted in medical visit and/or absenteeism [i.e. missing work or school]	Requires an emergency room (ER) visit or hospitalization
**Induration/Swelling (≥ 12 years of age)**	2.5 – 5 cm	5.1 – 10 cm	> 10 cm	Requires an emergency room (ER) visit or hospitalization
**Induration/Swelling (< 12 years of age)**	0.5 – 2 cm	2.0 -7.0 cm	> 7 cm	Requires an emergency room (ER) visit or hospitalization
**Erythema/Redness (≥ 12 years of age)**	2.5 – 5 cm	5.1 – 10 cm	> 10 cm	Requires an emergency room (ER) visit or hospitalization
**Erythema/Redness (< 12 years of age)**	0.5 – 2 cm	2.0 -7.0 cm	> 7 cm	Requires an emergency room (ER) visit or hospitalization
**Axillary (underarm) swelling or tenderness ipsilateral to side of injection**	Noticeable but does not interfere with activity	Interferes with activity but did not need a medical visit or absenteeism [i.e. missing work or school]	Significant; prevents daily activity and/or resulted in medical visit and/or absenteeism [i.e.	Requires an emergency room (ER) visit or hospitalization

Table 8. Injection-site Reactogenicity Grading				
**Local Reaction to Injectable Product**	**Mild (Grade 1)**	**Moderate (Grade 2)**	**Severe (Grade 3)**	**Potentially Life Threatening (Grade 4)**
			missing work or school]	

Participants/participant's LAR(s) will also be queried during Visits 2, 3, and 4 on common post-injection systemic symptoms as described in **Table 9**.

Table 9. Systemic Reactogenicity Grading (FDA modified)				
**Systemic**	**Mild (Grade 1)**	**Moderate (Grade 2)**	**Severe (Grade 3)**	**Potentially Life Threatening (Grade 4)**
**Fever (°C)** **(°F)**	38.0 - 38.4 100.4 - 101.1	38.5 - 38.9 101.2 - 102.0	39.0 – 40.0 102.1-104.0	> 40.0 >104.0
**Nausea/vomiting**	Noticeable but does not interfere with activity or 1 – 2 episodes/24 hours	Some interference with activity or > 2 episodes/24 hours	Significant; prevents daily activity and/or resulted in medical visit and/or absenteeism [i.e. missing work or school]	Requires an ER visit or hospitalization
**Diarrhea**	Noticeable but does not interfere with activity or 2 – 3 loose stools/24 hours	Some interference with activity or 4-5 loose stools/24 hours	Significant; prevents daily activity and/or resulted in medical visit and/or absenteeism [i.e. missing work or school] or 6 or more watery stools or > 24 hours	Requires an ER visit or hospitalization
**Headache**	Noticeable but does not interfere with activity	Some interference with activity but did not need a medical visit or absenteeism [i.e. missing work or school]	Significant; prevents daily routine activity and/or resulted in medical visit and/or absenteeism [i.e. missing work or school]	Requires an ER visit or hospitalization
**Fatigue**	Noticeable but does not interfere with activity	Some interference with activity but did not need a medical visit or absenteeism [i.e. missing work or school]	Significant; prevents daily routine activity and/or resulted in medical visit and/or absenteeism [i.e. missing work or school]	Requires an ER visit or hospitalization
**Myalgia**	Noticeable but does not interfere with activity	Some interference with activity but did not need a medical visit or absenteeism [i.e. missing work or school]	Significant; prevents daily routine activity and/or resulted in medical visit and/or absenteeism [i.e. missing work or school]	Requires an ER visit or hospitalization
**Arthralgia**	Noticeable but does not interfere with activity	Some interference with activity but did not need a medical visit or absenteeism [i.e. missing work or school]	Significant; prevents daily routine activity and/or resulted in medical visit and/or absenteeism [i.e. missing work or school]	Requires an ER visit or hospitalization
**Chills**	Noticeable but does not interfere with activity	Some interference with activity but did not need a medical visit or absenteeism [i.e. missing work or school]	Significant; prevents daily routine activity and/or resulted in medical visit and/or absenteeism [i.e. missing work or school]	Requires an ER visit or hospitalization

During Visits 2, 3, 4 participants/participant's LAR(s) will be encouraged to report any significant adverse events occurring within 7 days of the vaccination visits and during Visits 2, 3, 4, 5, and 6 any serious adverse events in an open-ended question format, e.g. "How are you or your child doing? Are you or your child having any medical or clinical problems? If so, please tell me about them." Participants/participant's LAR(s) who report severe solicited or unsolicited adverse events or express any concern about symptoms/unsolicited events will be encouraged to follow up with their or their child's primary care provider. Study staff will assist with coordination of referral appointments as necessary. Medical records will be obtained and reviewed for the occurrence of any adverse events, serious adverse events or adverse events of special interest throughout the study period.

Unsolicited adverse events (AEs) will be collected and reported for the 7 days following each vaccine visit. Serious adverse events (SAEs) and adverse events of special interest (AESI) will be collected and reported during the entire study period [i.e. from enrollment through 120 days following enrollment]. Unsolicited AEs, SAEs, and AESIs will be graded as follows.

**Mild (Grade 1)**	**Moderate (Grade 2)**	**Severe (Grade 3)**	**Potentially Life Threatening (Grade 4)**
Noticeable but does not interfere with activity or measurement	Interferes with activity but did not need a medical visit or absenteeism [i.e. missing work or school]	Significant; prevents daily activity and/or resulted in medical visit and/or absenteeism [i.e. missing work or school]	Requires an ER visit or hospitalization

An adverse event (AE) is any untoward medical occurrence in a patient or clinical investigation subject administered a pharmaceutical product and which does not necessarily have to have a causal relationship with this treatment.

An SAE is defined as an AE that meets one of the following conditions:

- Results in death during the period of protocol-defined surveillance
- Is life-threatening (defined as immediate risk of death at the time of the event)
- Requires inpatient hospitalization or prolonged hospitalization during the period of protocol-defined surveillance
- Results in congenital anomaly or birth defect
- Results in a persistent or significant disability/incapacity
- Any other important medical event that may not result in death, be life threatening, or require hospitalization, may be considered an SAE when, based upon appropriate medical judgment, the event may jeopardize the participant and may require medical or surgical intervention to prevent one of the outcomes listed above. Examples of such medical events include allergic bronchospasm requiring intensive treatment in an emergency room or at home, blood dyscrasias or convulsions that do not result in inpatient hospitalization, or the development of drug dependency or drug abuse.

An adverse event of special interest (AESI) includes the following:

- COVID-19 illness
- Multisystem inflammatory syndrome
- Guillain-Barre syndrome

- Allergic type reactions (including anaphylaxis, hives, or facial and limb swelling occurring within 7 days of a vaccination visit)
- Myocarditis or pericarditis

5.4.1 Causality (relatedness) Assessment

Study site investigators will assess relatedness to vaccine or study procedures (related, possibly related, unlikely related, or not related) for unsolicited AEs, SAEs and AEs of special interest. Relatedness determinations of these events will inform IRB reporting and safety monitoring (Section 5.4.3). Solicited symptoms in **Tables 8 and 9** will all be considered to be related to vaccine and causality assessment will not be done for these events. The study investigators will use their clinical judgement to make causality assessments and may consult the Expert Safety Panel or CISA Project for assistance with causality determinations. The final causality assessment decision is the responsibility of the site PI where the subject was enrolled.

5.4.2 Reporting of Adverse Events

COVID-19 vaccine administration will follow site-specific vaccine campaign administration guidelines in accordance with current recommendations from the US Food and Drug Administration and the Advisory Committee on Immunization Practices (ACIP). There are no previously published COVID-19 trials evaluating the concomitant administration of influenza vaccine; however, we do not foresee having a significant issue with serious adverse events (SAEs).

SAEs and unanticipated problems will be reported to the CDC and all participating IRBs according to institutional requirements.

Adverse events that occur in a recipient following COVID-19 vaccination should be reported to CDC's Vaccine Adverse Event Reporting System (VAERS). Vaccination providers are required by the Food and Drug Administration to report the following that occur after COVID-19 vaccination under Emergency Use Authorization:

- Vaccine administration errors
- Serious adverse events
- Cases of Multisystem Inflammatory Syndrome
- Cases of COVID-19 that result in hospitalization or death

Reporting is encouraged for any other clinically significant adverse event even if it is uncertain whether the vaccine caused the event. Information on how to submit a report to VAERS is available at https://vaers.hhs.gov external icon or by calling 1-800-822-7967.

5.4.3 Safety Monitoring Plan

As a lead site, Duke will coordinate an interim safety review. This review will be performed by a panel of vaccine safety experts. Duke will provide one subject matter expert to serve on the safety panel. CDC will appoint two subject matter experts from other CISA sites that are not participating in this study. The safety experts will review SAEs and AESI and create an interim report. This review will occur once approximately 100 subjects are enrolled or at the end of the influenza season, whichever occurs first. As the Lead Site, Duke will prepare narratives of the SAEs and AESI for the safety panel. This plan is designed to monitor safety while minimizing introduction of bias into the study and minimizing burden to study investigators.

5.5 Health-Related Quality of Life (HRQOL)

5.5.1 Generic Measures of HRQOL

HRQOL assessments will be performed for persons aged ≥12 years at baseline and daily for 7 days following receipt of either COVID-19 vaccine plus placebo or following COVID-19 vaccine plus influenza vaccine. Assessments will include the EQ-5D-5L and the EQ-VAS.

EQ-5D-5L

The EQ-5D is a standardized, generic measure of health status that provides information on HRQOL and activities of daily living: mobility, self-care, usual activities, pain/discomfort and anxiety/depression (http://www.euroqol.org/) [46]. In addition, the instrument contains the EQ Visual Analogue Scale (EQ-VAS) which measures the respondent's self-rated health.

The EQ-5D-5L is the new version of the EQ-5D that increases the levels of severity from three to five to significantly increase reliability and sensitivity while maintaining feasibility and reducing ceiling effects (**Appendix A**) [47,48]. The descriptive system comprises 5 dimensions of mobility, self-care, usual activities, pain/discomfort, anxiety /depression. For each of these dimensions, there are 5 response levels: no problems, slight problems, moderate problems, severe problems, and extreme problems. The respondent is asked to indicate his/her health state by ticking in the box against the most appropriate statement in each of the 5 dimensions. This decision results in a 1-digit number expressing the level selected for that dimension. The digits for 5 dimensions can be combined in a 5-digit number describing the respondent's health state from 11111 as best health and 55555 as worst health These numbers are converted to a Utility Index that ranges from -0.109 (worst health) to 1.000 (best health) for US specific values. The minimum clinically important difference ranges from 0.05 to 0.1 depending on health conditions being studied. The EQ-5D-5L will be used for participants 12 years of age and older. HRQOL will not be collected for participants 5-11 years of age.

EQ-VAS

The EQ-VAS records the respondent's self-rated health on a 20 cm vertical, visual analogue scale with endpoints labelled 'the best health you can imagine' (100) and 'the worst health you can imagine' (0). The respondent marks an 'X' on the scale number to indicate how their health is 'today.' The minimum clinically important difference on the VAS is 8.

The EQ-5D-5L and EQ-VAS have several advantages for use in this study. The measure is applicable to a wide range of health conditions and treatments and provide a simple descriptive profile and a single index value for health status. It has been validated in US and international populations and in adolescents and adults of all ages[46,49,50]. The measure is useful for monitoring the health status of patient groups at different moments in time and assessing the seriousness of conditions at different moments in time. The measure is designed for self-completion by respondents. It is simple, straightforward, take only a few minutes to complete and can easily be completed by adolescents and older adults. The instrument was designed to reduce respondent burden while achieving standards of precision for purposes of group comparisons involving multiple health dimensions. It has been widely used throughout the world in many different studies, including randomized controlled clinical trials, vaccine studies, and HRQOL studies. HRQOL will not be collected for participants aged 5-11 years of age.

5.6 Biospecimens Collection & Handling

5.6.1 Serum

Blood specimens will be collected during study visits as described in Tables 5 and 6.
All blood samples (≈ 5 or 10 mL, depending upon age of the participant) will be collected into serum separator tubes and processed as follows:

- Allow blood to clot at room temperature for at least 30 minutes while standing upright in a rack.
- Centrifuge tube within 8 hours of collection at 1100 to 1300 RCF(g) for 10 minutes.
- Gently remove the vacutainer stopper avoiding serum contamination with red blood cells. Using a single-use pipette, transfer 1.0 mL aliquots of serum (top layer) into 1.0mL or 1.8 mL cryovials, up to 5 cryovials are expected for those 12 years of age and older and up to 2 cryovials are expected for those 5-11 years of age. If less than 1 mL of processed serum is collected, it is a protocol deviation
- All cryovial aliquots will be barcode labelled and contain a unique identifier via REDCap. Numbers should be placed lengthwise on the tube.
- Freeze the cryovials at -80°C in the temperature-monitored research center freezer for future shipment.
- Serum aliquots will be stored in the Duke Human Vaccine Institute Accessioning Lab, the Johns Hopkins Center for Immunization Research laboratory, and at CCHMC Schubert Research Clinic Laboratory until planned HAI analyses at which point the samples stored at JHU and Cincinnati will be shipped to Duke for planned laboratory analysis

6 LABORATORY ANALYSES

6.1 Baseline SARS-CoV-2 antibody

Serum samples obtained at the Visit 1 (prior to vaccination) will be assayed for the presence of pre-existing SARS-CoV-2 antibody using the AdviseDx SARS-CoV-2 IgG II assay and the Alinitiy I SARS-CoV-2 IgG assay. The AdviseDx SARS-CoV-2 IgG II assay is a chemiluminescent microparticle immunoassay intended for the qualitative and semi-quantitative detection of IgG spike antibodies to SARS-CoV-2 in human serum. The Alinity I SARS-CoV-2 IgG assay is a chemiluminescent microparticle immunoassay intended for the qualitative detection of nucleocapsid IgG antibodies to SARS-CoV-2 in human serum. All results will be interpreted as either positive or negative. A positive result on any assay will be interpreted as a positive antibody test result. The assays are intended for use as an aid in identifying individuals with an adaptive immune response to SARS-CoV-2 indicating recent or prior infection. Both assays are currently only for use under the FDA's Emergency Use Authorization.
SARS-CoV-2 antibody assays will be performed in the Immunology Virology Quality Assessment Center (IVQAC) at the DHVI. The IVQAC is a GCLP laboratory which is also accredited by CAP, CLIA and ISO-17043.

6.2 Influenza Hemagglutination Inhibition (HAI) Assay

Influenza Hemagglutination Inhibition (HAI) Assays will be performed on sera collected, contingent on additional funding. Briefly, reference wild-type, reassortant, or vaccine virus strains representative of the specific viral antigens included in the 2021-22 influenza vaccine will be used to evaluate the relative levels of all four influenza strain-specific antibodies in

participant serum samples collected pre- and 21 or 28 days post-vaccination from all study participants. To accomplish these activities, all participant samples will be interrogated for influenza antibodies against the strains of interest using the influenza hemagglutination inhibition assay (HI). This assay is considered the “gold-standard” measure by which to evaluate seroconversion/seroprotection in response to seasonal influenza vaccination. This assay will be performed in accordance with the Duke Regional Biocontainment Laboratory Virology Unit’s fully optimized and approved SOP (RVUSOP004 Influenza HI of Serum Samples). Briefly, test samples will be assayed by HAI as duplicate 2-fold dilution series starting at 1:10. Serum dilutions are then incubated with a concentration of virus verified to possess a known potential for red blood cell (RBC) agglutination. The presence of virus-specific antibodies is visualized via incubation of the virus-serum mixture with a RBC solution; the endpoint titer for a given dilution series is then expressed as the reciprocal of the final dilution in which complete HAI is observed. By convention, seronegative samples are defined as having an endpoint HAI titer < 40 and seropositive samples as having an endpoint titer of ≥1:40; and seroconversion as a 4-fold change in endpoint titer relative to pre-immunization baseline or a change from <10 to ≥1:40[51].

6.3 COVID-19 Immunogenicity

The Duke NAb Laboratory for HIV and SARS-CoV-2 Vaccine Research and Development (Duke NAb Lab, PI: David Montefiori) will assess the magnitude, kinetics and duration of neutralizing antibody responses against SARS-CoV-2 by using a validated assay in an environment that operates in compliance with Good Clinical Laboratory Practices (GCLP).

Neutralization of SARS-CoV-2 Spike-pseudotyped viruses will be assessed in 293T/ACE2 cells as described in SOP “CFAR02-A0026 Measuring Neutralizing Antibodies Against SARS-CoV-2 Using Pseudotyped Virus and 293T/ACE2 Cells.” This assay has been formally validated and is part of Drug Master File # 26862 with the US Federal Drug Administration. Assay validation was performed with human serum samples and monoclonal antibodies using the D614G form of the Wuhan-1 Spike, which is the dominant form of Spike protein in the global epidemic. This assay has also been validated using B.1.351(246R) and B.1.1.529 (BA1) and may be validated for other newly emerged variants as needed.

Neutralization is measured by using lentiviral particles pseudotyped with SARS-CoV-2 Spike and containing a firefly luciferase (Luc) reporter gene for quantitative measurements of infection by relative luminescence units (RLU). SARS-CoV-2 Spike-pseudotyped viruses are prepared and titrated for infectivity by using mutated forms of an expression plasmid encoding codon-optimized full-length Spike of the Wuhan-1 strain (VRC7480) provided by Drs. Barney Graham and Kizzmekia Corbett at the Vaccine Research Center, National Institutes of Health (USA). Mutations were introduced into VRC7480 by site-directed mutagenesis using the QuikChange Lightning Site-Directed Mutagenesis Kit from Agilent Technologies. All mutations were confirmed by full-length Spike gene sequencing. Pseudovirions are produced in HEK 293T/17 cells by transfection using Fugene 6 Transfection Reagent and a combination of Spike plasmid, lentiviral backbone plasmid (pCMV ΔR8.2) and firefly Luc reporter gene plasmid (pHR' CMV Luc) in a 1:17:17 ratio in Opti-MEM (Life Technologies). Pseudovirions are titrated for infectious dose (TCID50) by making serial 3-fold or 5-fold dilutions in quadruplicate for a total of 11 dilutions in 96-well flat-bottom plates.

A pre-titrated dose of virus is incubated with 8 serial 5-fold dilutions of serum samples in duplicate in a total volume of 150 µl for 1 hr at 37°C in 96-well flat-bottom culture plates. Cells are detached using TrypLE Select Enzyme solution, suspended in growth medium (100,000 cells/ml) and immediately added to all wells (10,000 cells in 100 µL of growth medium per well). One set of 8 wells receives cells + virus (virus control) and another set of 8 wells receives cells only (background control). After 71-73 hrs of incubation, medium is removed by gentle aspiration and 30 µl of Promega 1X lysis buffer is added to all wells. After a 10 minute incubation at room temperature, 100 µl of Bright-Glo luciferase reagent is added to all wells. After 1-2 minutes, 110 µl of the cell lysate is transferred to a black/white plate. Luminescence is measured using a GloMax Navigator luminometer (Promega). Neutralization titers are the serum dilution at which RLUs are reduced by either 50% (ID50) or 80% (ID80) compared to virus control wells after subtraction of background RLUs. Serum samples are heat-inactivated for 30 minutes at 56°C prior to assay.

For assay internal quality controls (IQC), a qualified positive control is tested on each assay plate (plate control). Assay run controls may consist of COVID-19 convalescent serum samples or SARS-CoV-2 vaccine recipients with high, medium and low ID50 and ID80 titers against the test virus, plus a normal human serum negative control. Levey-Jennings plots will be generated during testing and checked for violations of standards that are based on the Westgard Rules. Violations of the Westgard Rules are investigated in real-time. ***6.4 Future Studies***

In addition to the specified analyses described thus far, there may be other tests or assays that have yet to be identified that may be important for interpreting our study findings or of relevance to co-administration of mRNA COVID-19 vaccines and IIV4 simultaneously. Additional laboratory assays may test for antibodies against other bacteria or viruses, markers of inflammation, or used in research assessing vaccine co-administration or health outcomes. Specimens banked for use in other studies will be linked to information (including identifying information) that participants provided to the study. Because it is unknown if future testing will be of any utility, results of future testing will not be provided.

7 STATISTICAL CONSIDERATIONS

In collaboration with Johns Hopkins University and Cincinnati Children's Hospital sites, the research team at Duke will oversee the statistical analysis. Data will reside on a secure Duke server maintained by Duke Health Technology Solutions (DHTS). For the study, a database will be developed and a data set for the study without personal identifiers will be made available to the CDC upon request. Duke statisticians will develop a comprehensive Statistical Analysis Plan. The summary points of the analysis plan are presented below.

7.1 Analysis Plan

Should an interim safety analysis be required, the alpha level will be adjusted to assure the overall type I error is maintained at the one-sided alpha 0.025 level for the primary outcome of non-inferiority.

7.1.1 Sample Size

Safety: Four reactogenicity events were included in the primary statistical endpoint: fever, chills, myalgia, or arthralgia of moderate or greater severity. These were considered clinically meaningful and were solicited in both mRNA COVID-19 vaccine trials. Headache and fatigue were also solicited reactogencity in both trials, but each occurred at the moderate-severe grade in >10% of participants after dose 1 placebo (in the Pfizer- Pfizer-BioNTech trial) and were thought to be less specific[13]. Based on data from prelicensure studies[10,52,53], we assume that 15% of participants will have at least one reaction of moderate severity or greater to include fever, chills, myalgia, or arthralgia reactogenicity event in the Sequential group. We have selected a clinically meaningful noninferiority margin of 10%. We plan to recruit a total of 450 participants and assume a 5% drop out rate, leaving N=428 or N=214 per study group. Statistical calculations show that with a one-side alpha level of 0.025, and 214 subjects in each group across all study sites, there is 82.6% power to be able to demonstrate that the proportion of participants with at least one severe solicited local or systemic reactogenicity event in the Simultaneous group is noninferior to the Sequential group. Enrollment in this study shall occur during two influenza season (2021-22, 2022-23).

7.1.2 Analysis Populations

Full Analysis Population 1:

- This population is defined as all subjects who are randomized, vaccinated, and provide at least one day of complete data on the symptom diary.

Full Analysis Population 2:

- This population is defined as all subjects who are randomized and vaccinated.

Immunogenicity Population:

- This population defined as subjects who received vaccine, provide baseline and Visit 3 and Visit 4 blood draws of acceptable volume and quality within the protocol-defined time frame with no protocol violations affecting immunogenicity. Protocol violations affecting the immunogenicity analyses will be defined in the Statistical Analysis Plan (SAP). The acceptable visit window for sample analysis will be -7/+14 days from the target date. Any subject with sufficient data for either or both of the influenza and SARS-CoV-2 analysis will be included in this population. Should 10% or more of the total study population be excluded from the Immunogenicity Population due to missing the visit window, then an additional analysis will be performed including these subjects with the missing visit window exclusion.

The Full Analysis Population 1 is the primary population for analysis unless otherwise stated.

7.1.3 Primary Objective 1

- To compare the proportion of participants with moderate or more severe fever, chills, myalgia, or arthralgia in the group receiving IIV4 simultaneously with mRNA COVID-19 vaccine at Vaccination Visit 1 (Simultaneous group) with the group receiving IIV4 alone one to two weeks later at Vaccination Visit 2 (Sequential group) following both Vaccination Visit 1 and 2
    - Hypothesis: The proportion of participants with moderate or more severe fever, chills, myalgia or arthralgia will be noninferior (not higher) in the Simultaneous group versus the Sequential group.

This objective will be assessed for visits 1 and 2 using a one-sided noninferiority test with the alpha level set at 0.025 and noninferiority margin of 10%. The null hypothesis is the Simultaneous group is inferior (i.e., Simultaneous group will have a higher proportion) to the Sequential group in regards to the proportion of participants with at least one moderate or severe fever, chills, myalgia, or arthralgia event after visits 1 and 2.

Ho: Simultaneous group - Sequential group ≥ 0.10 (10%)

The alternative hypothesis is the Simultaneous group is noninferior to the Sequential group in regards to the proportion of participants with at least one moderate or severe fever, chills, myalgia, or arthralgia event after visits 1 and 2.

Ha: Simultaneous group - Sequential group < 0.10 (10%)

The upper bound of a site-stratified Newcombe binomial confidence interval (Yan and Su 2010) with Cochran-Mantel-Haenszel (CMH) weighting of the difference will be used to make these assessments with no adjustment to the alpha level for multiple comparisons.

7.1.4 Secondary Objective 1

- To compare the proportion of participants with moderate or more severe fever, chills, myalgia, or arthralgia in the Simultaneous versus the Sequential Group following the first vaccination visit

7.1.5 Secondary Objective 2

- To compare the proportion of participants with moderate or more severe fever, chills, myalgia, or arthralgia in the Simultaneous versus Sequential Group following the second vaccination visit

Secondary objectives 1 and 2 will be evaluated using a Mantel-Haenszal statistic in a stratified analysis by site at the alpha 0.05 level. No adjustments will be made to the alpha level for these evaluations.

7.1.6 Secondary Objective 3

- To describe the proportions of participants in the Simultaneous and Sequential vaccination groups with solicited local and systemic reactogenicity events according to severity grade after the first and second vaccination visit and third vaccination visit for those receiving two doses of mRNA COVID-19 vaccine

Tables (one for each visit 1, 2, and 3) will be produced that summarize each solicited local and systemic reactogenicity event by classification (none, mild, moderate, and severe), as well as by moderate or severe for each study group. These tables will have the number and percentage for each classification by study group and the confidence interval of the difference between the study groups.

7.1.7 Secondary Objective 4

- To describe the proportions of participants in the Simultaneous and Sequential vaccination groups experiencing at least one serious adverse event and a description of these events

A table will be produced that summarize participants experiencing at least one serious adverse event during the study period by group. This table will have the number and percentage for each outcome by study group and the confidence interval of the difference between the study groups. Listings with the clinical narratives will also be provided. The primary analysis population will be the Full Analysis Population 2.

7.1.8 Exploratory Objectives

The analysis for the exploratory objectives will be detailed in the comprehensive Statistical Analysis Plan. The Immunogenicity Population will be the primary analysis population for exploratory HAI and COVID immunogenicity objectives.

7.2 Data Management

The novel Vanderbilt-designed resource developed specifically for online collection of research information, the Research Electronic Data Capture (REDCap) platform (https://projectredcap.org/), will be used to design study forms, including the reaction forms, and short customized questionnaires to collect information from study subjects. REDCap provides: 1) a streamlined process for rapidly building a database; 2) an intuitive interface for collecting data, with data validation and audit trail; 3) automated export procedures for seamless data downloads to common statistical packages; 4) branching logic, file uploading, and calculated fields; and 5) a quick and easy protocol set-up. This system will be used by Duke for data management. All electronic linkages will fulfill regulations for protection of human subjects and requirements to minimize the risk of breach of confidentiality.

All study-related documents containing protected health information, e.g. enrollment logs, case report forms, diaries completed by study participants, will be maintained in secure research offices at Duke, John Hopkins CIR, and Cincinnati Children's Hospital, which are accessible to research staff only.

The study team will utilize a secure, encrypted, file transfer method for sharing study documents and data with the CDC. No personal identifiers will be included in any shared documents or datasets.

7.2.1 Research Electronic Data Capture (REDCap)

REDCap (http://project-redcap.org/), assists with the collection and management of data for diverse clinical and translational research studies. REDCap was designed around the concept of giving research teams an easy method to specify project needs and rapidly develop secure, web-based applications for collection, management and sharing of research data. REDCap accomplishes these key functions through use of a single study metadata table referenced by presentation-level operational modules. Based on this abstracted programming model, databases are developed in an efficient manner with little resource investment beyond the creation of a single data dictionary. The concept of metadata-driven application development is

well established, and the critical factor for successful data collection lies in creating a simple workflow methodology allowing research teams to autonomously develop study-related metadata in an efficient manner. Both products include secure institutional data hosting and include full audit-trails in compliance with Health Insurance Portability and Accountability Act (HIPAA) security requirements. The REDCap Consortium is comprised of 647 active institutions. The REDCap currently supports 68,000 projects with over 89,000 users spanning numerous research focus areas across the consortium. The current project will use this software application for the design of electronic forms to collect information from study participants, to link the baseline data, sample collection date, and laboratory results in an automated database family, to perform data cleaning and data quality assurance efficiently, and to design an analytical dataset for the analysis of the project data.

Data will be entered into the REDCap database by members of the study team from Duke, Johns Hopkins CIR, and Cincinnati Children's Hospital using the paper case report forms utilized to record data collected as part of study procedures. Study investigators will be responsible for assuring that all paper records are securely stored according to the requirements of their IRBs. The study investigators will be responsible for assuring the accuracy of the data entered from the paper forms into REDCap. Assigned identifiers will be used in REDCap.

In order to perform data cleaning and data quality assurance efficiently, numerous built-in filters and checks for consistency of the data including range and limit checks, branching logic and pull down menus to limit choices for categorical variables to a pre-specified list will be implemented and performed automatically to minimize data entry error. The data will be randomly sampled and checked against source records on a regular basis. The data and related analytical datasets will also be stored at the lead and contributing sites with secured password-protected computers.

7.3 Role of the CDC Investigators in the Project

This study is funded by a CDC contract with Duke University, Johns Hopkins University and Cincinnati Children's Hospital as Task Orders in the CISA Project Contract. CDC staff will collaborate with all sites to develop the protocol, conduct the study, ensure the study is aligned with US Department of Health and Human Services (CDC) public health priorities, and analyze the data and disseminate the results. CDC may receive access to coded data not containing any directly identifying information.

8 HUMAN SUBJECTS

8.1 Human Subjects Involvement, Characteristics, and Design

Duke, Johns Hopkins University, and Cincinnati Children's Hospital investigators will be responsible for submitting the protocol, informed consent, diaries, recruitment letters, flyers, and any written or verbally conveyed materials specific to this project to their institutional review boards. CDC staff will be responsible for submitting materials to the CDC Human Subjects Review for determination to rely on Duke IRB.

To facilitate subject recruitment at the practices, we will request a waiver of consent and HIPAA authorization for ascertainment (identification, selection) and/or recruitment of potential subjects

while recording identifiable private health information (PHI) prior to obtaining the subject's consent. This information will be obtained from review of the electronic scheduling and medical record systems in the clinics in order to determine eligibility for study enrollment where available. We will review only the minimum amount of information necessary to determine eligibility, i.e. date of birth, medical and surgical history, and recent laboratory test results. The PHI collected prior to consent will be used to recruit and screen only. Use of PHI in this manner involves no more than minimal risk to subjects and no information will leave the study sites.

Requests for continuing review, when required, will be submitted at each engaged institution in accordance with institutional procedures. Protocol deviations or concerns about study integrity will be reported promptly to the overseeing IRB or CDC in accordance with institutional requirements.

8.2 Sources of Material
Medical history and immunization history will be obtained from the medical record when available and from patient report. Demographic information will be obtained from the medical record and patient report. Subjects will record solicited adverse reactogenicity events and any medical intervention sought on study days 1-7 following each vaccination visit on the symptom diary. Diary information will either be reported electronically or on paper which will be given to the study team during a study visit. Diary information reported electronically will be preferred but paper reporting will be allowed. The research staff will assess oral temperature.

8.3 Potential Risks and Benefits

<u>mRNA COVID-19 vaccines</u>
Two COVID-19 vaccine received Emergency Use Authorization (EUA) by the FDA in December 2020. BNT162b2 received FDA approval for individuals ≥ 16 years of age and mRNA-1273 received authorization for those ≥18 years of age. The Pfizer/BioNTech mRNA COVID-19 vaccine received additional EUA for adolescents 12 to 15 years in May 2021 and children 5 to 11 years in October 2021. These vaccines have been recommended at these ages by the ACIP. Participants will be provided with the respective fact sheets for the mRNA COVID-19 vaccine they receive including the BNT162b2 https://www.fda.gov/media/144414/download and the MRNA-1273 https://www.fda.gov/media/144638/download
Side effects that have been reported with mRNA COVID-19 vaccines include both injections site reactions as well as general side effects. Injection site reactions include: pain, tenderness and swelling of the lymph nodes in the same arm of the injection, swelling (hardness), and redness at the injection site. General side effects include: fatigue, headache, muscle pain, joint pain, chills, nausea and vomiting, fever and feeling unwell. There is a remote chance that an mRNA COVID-19 vaccine could cause a severe allergic reaction, usually occurring within a few minutes to hours after getting a dose of vaccine. Signs of a severe allergic reaction can include: difficulty breathing, facial and throat swelling, tachycardia, total body rash, dizziness and weakness. There have been rare reports of cases of inflammation of the heart—called myocarditis and pericarditis—happening after mRNA COVID-19 vaccination[30-32]. The events have mainly occurred in adolescents and young adults and more often after the second dose of vaccine. Available data from short-term follow-up suggest that most individuals have had resolution of symptoms, but information is not yet available about potential long-term sequelae[30,31]. These may not be all the possible side effects of the mRNA COVID-19 vaccines.

Serious and unexpected side effects may also occur. mRNA COVID-19 vaccines are still being studied in clinical trials. Available data support the safety of dose 3 mRNA COVID-19 vaccine in immunocompromised individuals[13]. Available data support the safety of booster doses of mRNA COVID-19 vaccine[13].

SD-IIV4 and HD-4
SD-IIV4 and HD-IIV4 are FDA-licensed vaccines approved for use in persons ≥ 6 months of age and those ≥65 years old, respectively. Both vaccines are standard clinical practice and recommended by the CDC. Participants will be provided with the CDC Vaccine Information Statement (VIS) for IIV4 (https://www.cdc.gov/vaccines/hcp/vis/vis-statements/flu.pdf).

IIV4 risks include minor problems such as soreness, redness, swelling, or pain where the shot was given, hoarseness, sore, red or itchy eyes, cough, fever, aches, headache, itching, fatigue, all of which usually occur within 1-2 days of vaccination and are self-limiting. More serious problems including a small increased risk of Guillain-Barré Syndrome estimated at 1 or 2 additional cases per million people vaccinated can occur. This is much lower than the risk of severe complications from influenza infection, which can be prevented by IIV4[54]. In addition, any medication can cause a severe allergic reaction, or anaphylaxis, which is estimated at ~ 1 in one million doses of IIV4 administered[55].

General Vaccine Risks
Some people get severe pain in the shoulder and have difficulty moving the arm where a shot was given. This happens very rarely. Syncope (fainting) can occur in association with administration of injectable vaccines. Sitting or lying down when space is available for about 15 minutes can help prevent fainting, and injuries caused by a fall, as recommended in the ACIP General Recommendations on Immunization [8]. Subjects should inform their doctor if they feel dizzy or have vision changes or ringing in the ears.

As with any licensed or authorized vaccine, protection may not occur in 100% of vaccinated persons for either COVID-19 or influenza vaccines.

Blood Drawing
Risks of blood drawing include pain, swelling, bleeding, or bruising at the site where the blood sample is collected. Subjects may also experience dizziness or fainting. There is a small risk of infection around the vein where the blood was collected. Each study subject will be asked to have up to 4 blood samplings with the total volume not to exceed 60mL over an approximately 4 month period of time.

Delay of Influenza Protection
There is a potential risk of a short delay in influenza protection by delaying the receipt of influenza vaccine 2 weeks.

Confidentiality
An additional risk of study participation is the potential for loss of confidentiality.

8.4 Adequacy of Protection Against Risks

8.4.1 Protections against Risk

To decrease the possibility of infection at the site of blood drawing, the area on the arm above the vein where blood will be taken will be prepped with 70% isopropyl alcohol antiseptic prior to venipuncture.

Subjects/subject's LAR(s) will be counseled on possible side effects following vaccination and followed closely in the immediate post-vaccination period and during the following week for assessment of moderate to severe local or systemic reactogenicity. In the immediate post-vaccination period, all subjects will be monitored in a sitting or lying position for 30 minutes following vaccinations to help prevent fainting, and injuries caused by a fall. Subjects with a prior history of severe allergic reaction after a previous dose of any influenza vaccine or COVID-19 vaccine, or to a vaccine component, including egg protein, will be excluded from study enrollment. Epinephrine and equipment for managing an airway will be available for immediate use if needed to treat a severe allergic reaction or anaphylaxis. Subjects will be evaluated and cared for as described in the Unscheduled Visit section above

Data Safety monitoring, as described above **(Section 5.4.3)**, shall also be done.

The study team will provide documentation to the participant and primary care provider regarding receipt of COVID-19 vaccine and influenza vaccine. Documentation of COVID-19 vaccine receipt will be provided on the date received. Documentation of influenza vaccine receipt will be provided at Visit 2 when it can be assured that all participants will have received an influenza vaccine.

Participants will be counseled that if they are concerned that they have an influenza-like illness (characterized by fever and irritability, cough, sore throat, or vomiting) or symptoms of COVID-19 which may overlap with influenza, they should seek care for with their health care provider. If the provider suspects or determines that the participant has influenza, there are medications to treat influenza illness and the provider may opt to treat the participant with one of these medications. If a participant's care requires the identity of the date of receipt of influenza vaccine, blinding will be broken for that patient. At the end of the study, the participants/participant's LAR(s) and providers will receive documentation about the timing of influenza vaccination for each participant. If influenza activity become widespread, study enrollment may be paused. The National Institutes of Health (NIH) provides treatment and management recommendations for COVID-19 patients https://www.covid19treatmentguidelines.nih.gov/.

Every effort possible will be made to keep information about participants confidential. Computerized participant information will be kept in password-protected files on secured servers. Paper case report forms will be kept in locked files belonging to the study personnel. Any publications resulting from this work will not contain any identifiable participant information.

The CDC provides a Certificate of Confidentiality to protect the privacy of research participants. This certificate limits the disclosure of subject identifiable information. The investigators cannot disclose identifiable information except for if required by law, if participant consented to disclosure or if the information is used for other scientific research. Information may be disclosed to the CDC or the FDA.

8.4.2 ClinicalTrials.gov Requirements

The project is registered on ClinicalTrials.gov (NCT05028361).

8.5 Human Subjects

In obtaining and documenting informed consent, the Investigator and study team will comply with the applicable regulatory requirements, Good Clinical Practices, and ethical principles. The written informed consent form must be signed and dated by the study participant prior to initiation of any study activities.

8.5.1 Vulnerable Subjects Research

Children are a vulnerable research population and require additional protections when they are potential research subjects. Children will receive approved or authorized vaccines consistent with ACIP recommendations. All children will receive an influenza vaccine at one time point and will receive a placebo at one time point. Thus, all children enrolled in the study will be immunized against seasonal influenza. Because this study is minimal risk with the potential for benefit, the permission of only one parent/LAR will be obtained. Assent from participants 5 through 17 years of age will be obtained in a manner consistent with local IRB regulations. For participants turning 18 years of age during the course of the study, parental consent will be obtained at enrollment and written informed consent will be obtained from the participant after their 18th birthday.

REFERENCES

1. Patel A, Jernigan DB, nCo VCDCRT. Initial Public Health Response and Interim Clinical Guidance for the 2019 Novel Coronavirus Outbreak - United States, December 31, 2019-February 4, 2020. MMWR Morb Mortal Wkly Rep 2020;69(5):140-146. DOI: 10.15585/mmwr.mm6905e1.
2. Organization WH. WHO Director-General's opening remarks at the media briefing on COVID-19-11 March 2020. Geneva, Switzerland2020.
3. COVID Data Tracker. Centers for Disease Control and Prevention. (https://covid.cdc.gov/covid-data-tracker/).
4. Prevention CfDCa. SARS-CoV-2 Variant Classifications and Definitions.
5. Prevention CfDCa. People with Certain Medical Conditions. (https://www.cdc.gov/coronavirus/2019-ncov/need-extra-precautions/people-with-medical-conditions.html?CDC_AA_refVal=https%3A%2F%2Fwww.cdc.gov%2Fcoronavirus%2F2019-ncov%2Fneed-extra-precautions%2Fgroups-at-higher-risk.html).
6. Simonsen L. The global impact of influenza on morbidity and mortality. Vaccine 1999;17 Suppl 1:S3-10. DOI: 10.1016/s0264-410x(99)00099-7.
7. Glezen WP. Serious morbidity and mortality associated with influenza epidemics. Epidemiol Rev 1982;4:25-44. DOI: 10.1093/oxfordjournals.epirev.a036250.
8. Lisa A. Grohskopf EA, Karen R. Broder, Lenee H. Blanton, Alicia M. Fry, Daniel B. Jernigan, Robert L. Atmar. Prevention and Control of Seasonal Influenza with Vaccines: Recommendations of the Advisory Committee on Immunization Practices — United States, 2020–21 Influenza Season. Centers for Disease Control and Prevention. (https://www.cdc.gov/mmwr/volumes/69/rr/rr6908a1.htm?s_cid=rr6908a1_w).
9. Thompson WW, Shay DK, Weintraub E, et al. Influenza-associated hospitalizations in the United States. JAMA 2004;292(11):1333-40. DOI: 10.1001/jama.292.11.1333.
10. Thompson WW, Shay DK, Weintraub E, et al. Mortality associated with influenza and respiratory syncytial virus in the United States. JAMA 2003;289(2):179-86. DOI: 10.1001/jama.289.2.179.
11. Ozaras R, Cirpin R, Duran A, et al. Influenza and COVID-19 coinfection: Report of six cases and review of the literature. J Med Virol 2020;92(11):2657-2665. DOI: 10.1002/jmv.26125.
12. ClinicalTrials.gov. Bethesda, MD: National Library of Medicine; 2021.
13. COVID-19 Vaccines Food and Drug Administration. (https://www.fda.gov/emergency-preparedness-and-response/coronavirus-disease-2019-covid-19/covid-19-vaccines).
14. FDA Authorizes Pfizer-BioNTech COVID-19 Vaccine for Emergency Use in Children 5 through 11 Years of Age. U.S. Food and Drug Administration; 2021.
15. Polack FP, Thomas SJ, Kitchin N, et al. Safety and Efficacy of the BNT162b2 mRNA Covid-19 Vaccine. N Engl J Med 2020;383(27):2603-2615. DOI: 10.1056/NEJMoa2034577.
16. Baden LR, El Sahly HM, Essink B, et al. Efficacy and Safety of the mRNA-1273 SARS-CoV-2 Vaccine. N Engl J Med 2021;384(5):403-416. DOI: 10.1056/NEJMoa2035389.
17. Interim Clinical Considerations for Use of COVID-19 Vaccines Currently Authorized in the United States. Center for Disease Control and Prevention. (https://www.cdc.gov/vaccines/covid-19/clinical-considerations/covid-19-vaccines-us.html).

18. COVID-19 ACIP Vaccine Recommendations. Centers for Disease Control and Prevention. (https://www.cdc.gov/vaccines/hcp/acip-recs/vacc-specific/covid-19.html).
19. Coronavirus (COVID-19) Update: FDA Authorizes Additional Vaccine Dose for Certain Immunocompromised Individuals. FDA. (https://www.fda.gov/news-events/press-announcements/coronavirus-covid-19-update-fda-authorizes-additional-vaccine-dose-certain-immunocompromised).
20. Prevention CfDCa. COVID-19 Vaccine Booster Shot Centers for Disease Control and Prevention. (https://www.cdc.gov/coronavirus/2019-ncov/vaccines/booster-shot.html).
21. Prevention CfDCa. Joint Statement from HHS Public Health and Medical Experts on COVID-19 Booster Shots (https://www.cdc.gov/media/releases/2021/s0818-covid-19-booster-shots.html).
22. CDC Expands Eligibility for COVID-19 Booster Shots to All Adults. Centers for Disease Control and Prevention; 2021.
23. CDC Expands COVID-19 Booster Recommendations to 16-and-17-year-olds. Centers for Disease Control and Prevention; 2021.
24. Kate R. Woodworth MDM, MPH; Jennifer P. Collins, MD; Stephen C. Hadler, MD; Jefferson M. Jones, MD; Sujan C. Reddy, MD;, Mary Chamberland MDC-O, MD; Rebecca L. Morgan, PhD; Oliver Brooks, MD; H. Keipp Talbot, MD; Grace M. Lee, MD;, Beth P. Bell MMFD, MD; Sarah Mbaeyi, MD; Kathleen Dooling, MD; Sara E. Oliver, MD. The Advisory Committee on Immunization Practices' Interim Recommendation for Use of Pfizer-BioNTech COVID-19 Vaccine in Children Aged 5–11 Years — United States, November 2021. Morbidity and Mortality Weekly Report. Centers for Disease Control and Prevention, November 21, 2021 2021. (https://www.cdc.gov/mmwr/volumes/70/wr/pdfs/mm7045e1-H.pdf).
25. Administration FaD. FDA Briefing Document: Pfizer-BioNTech COVID-19 Vaccine. Vaccine and Related Biological Products Advisory Committee Meeting2020.
26. Administration FaD. FDA Briefing Document: Moderna COVID-19 Vaccine. Vaccine and Releated Biological Products Advisory Committee Meeting2020.
27. Frenck RW, Jr., Klein NP, Kitchin N, et al. Safety, Immunogenicity, and Efficacy of the BNT162b2 Covid-19 Vaccine in Adolescents. N Engl J Med 2021. DOI: 10.1056/NEJMoa2107456.
28. E.B. Walter KRT, C. Sabharwal, A. Gurtman, S. Lockhart, G.C. Paulsen, E.D. Barnett, F.M. Muñoz, Y. Maldonado, B.A. Pahud, J.B. Domachowske, E.A.F. Simões, U.N. Sarwar, N. Kitchin, L. Cunliffe, P. Rojo, E. Kuchar, M. Rämet, I. Munjal, J.L. Perez, R.W. Frenck, Jr., E. Lagkadinou, K.A. Swanson, H. Ma, X. Xu, K. Koury, S. Mather, T.J. Belanger, D. Cooper, Ö. Türeci, P.R. Dormitzer, U. Şahin, K.U. Jansen, and W.C. Gruber. Evaluation of the BNT162b2 Covid-19 Vaccine in Children 5 to 11 Years of Age. N Engl J Med 2021:35-46. DOI: 10.1056/NEJMoa2116298.
29. Shimabukuro TT, Cole M, Su JR. Reports of Anaphylaxis After Receipt of mRNA COVID-19 Vaccines in the US-December 14, 2020-January 18, 2021. JAMA 2021;325(11):1101-1102. DOI: 10.1001/jama.2021.1967.
30. Emergency Use Authorization (EUA) of the Pfizer-BioNTech COVID-19 Vaccine to Prevent Coronavirus Disease 2019 (COVID-19). Fact Sheet for Healthcare Providers Administering Vaccine(Vaccination Providers) U.S. Food and Drug Administration, 2021. (https://www.fda.gov/media/144413/download).
31. Emergency Use Authorization (EUA) of the Moderna COVID-19 Vaccine to Prevent Coronavirus Disease 2019 (COVID-19). Fact Sheet for Healthcare Providers

Administering Vaccine (Vaccination Providers). U.S. Food and Drug Administration (FDA), 2021. (https://www.fda.gov/media/144637/download).

32. Clinical Considerations: Myocarditis and Pericarditis after Receipt of mRNA COVID-19 Vaccines Among Adolescents and Young Adults. Centers for Disease Control and Prevention. (https://www.cdc.gov/vaccines/covid-19/clinical-considerations/myocarditis.html).
33. Moulia D. Myocarditis and COVID-19 Vaccine Intervals: Internaltional Data and Policies Centers for Disease Control and Prevention; 2021.
34. Comirnaty (COVID-19 Vaccine, mRNA) Evaluation of a Booster Dose (Third Dose) In: Committee VaRBPA, ed.: Food and Drug Administration; 2021:1-39.
35. FACT SHEET FOR HEALTHCARE PROVIDERS ADMINISTERING VACCINE (VACCINATION PROVIDERS) EMERGENCY USE AUTHORIZATION (EUA) OF THE MODERNA COVID-19 VACCINE TO PREVENT CORONAVIRUS DISEASE 2019 (COVID-19). 2022. (https://www.modernatx.com/covid19vaccine-eua/eua-fact-sheet-providers.pdf).
36. Marks P. CBER assessment of a second booster dose of the PfizerBioNTech COVID-19 Vaccine (0.3 mL) administered following a first booster dose of any FDA authorized or approved COVID-19 vaccine in certain individuals. Review Memorandum: FDA US Food and Drug; 2022.
37. Lisa A. Grohskopf EA, Karen R. Broder, Lenee H. Blanton, Alicia M. Fry, Daniel B. Jernigan, Robert L. Atmar. Prevention and Control of Seasonal Influenza with Vaccines: Recommendations of the Advisory Committee on Immunization Practices — United States, 2020–21 Influenza Season. Recommendations and Reports Centers for Disease Control and Prevention, 2021. (https://www.cdc.gov/mmwr/volumes/69/rr/rr6908a1.htm).
38. Grohskopf LA, Blanton LH, Ferdinands JM, et al. Prevention and Control of Seasonal Influenza with Vaccines: Recommendations of the Advisory Committee on Immunization Practices - United States, 2022-23 Influenza Season. MMWR Recomm Rep 2022;71(1):1-28. DOI: 10.15585/mmwr.rr7101a1.
39. Influenza(Flu) Vaccine (Inactivated or Recombinanat): What you need to know. Vaccine Information Sheet. Centers for Disease Control and Prevention, 2019. (https://www.cdc.gov/vaccines/hcp/vis/vis-statements/flu.html).
40. Prevention CfDCa. Interim Clinical Considerations for Use of COVID-19 Vaccines Currently Authorized in the United States alert icon. (https://www.cdc.gov/vaccines/covid-19/info-by-product/clinical-considerations.html).
41. Ruvim Izikson DB, Jean-Sébastien Bolduc, Pierre Bourron, Marion Fournier, Tamala Mallett Moore, Aseem Pandey, Lucia Perez,, Nessryne Sater AS, Sophie Wague, Sandrine I Samson. Safety and immunogenicity of a high-dose quadrivalent influenza vaccine administered concomitantly with a third dose of the mRNA-1273 SARS-CoV-2 vaccine in adults aged ⩾ 65 years: a phase 2, randomised, open-label study. Lancet Respir Med 2022. DOI: 10.1016/S2213-2600(21)00557-9.
42. Sara E. Oliver MMW, DrPH; Isaac See, MD; Sarah Mbaeyi, MD1; Monica Godfrey, MPH; Stephen C. Hadler, MD; Tara C. Jatlaoui, MD; Evelyn Twentyman, MD; Michelle M. Hughes, PhD; Agam K. Rao, MD; Anthony Fiore, MD; John R. Su, MD, PhD; Karen R. Broder, MD; Tom Shimabukuro, MD; Allison Lale, MD; David K. Shay, MD; Lauri E. Markowitz, MD; Melinda Wharton, MD; Beth P. Bell, MD; Oliver Brooks, MD; Veronica McNally, JD; Grace M. Lee, MD; H. Keipp Talbot, MD; Matthew F. Daley, MD. Use of the Janssen (Johnson & Johnson) COVID-19 Vaccine: Updated Interim Recommendations

from the Advisory Committee on Immunization Practices - United States, December 2021. MMWR and Morb Mortal Wkly Rep. Centers for Disease Control and Prevention, 2022.

43. COVID-19 Vaccine Emergency Use Authorization (EUA) Fact Sheets for Recipients and Caregivers. Centers for Disease Control and Prevention. (https://www.cdc.gov/vaccines/covid-19/eua/index.html).
44. Vaccines Licensed for Use in the United States U.S. Food and Drug Administration. (https://www.fda.gov/vaccines-blood-biologics/vaccines/vaccines-licensed-use-united-states).
45. Chang LJ, Meng Y, Janosczyk H, Landolfi V, Talbot HK, Group QHDS. Safety and immunogenicity of high-dose quadrivalent influenza vaccine in adults >/=65years of age: A phase 3 randomized clinical trial. Vaccine 2019;37(39):5825-5834. DOI: 10.1016/j.vaccine.2019.08.016.
46. Brazier J, Jones N, Kind P. Testing the validity of the Euroqol and comparing it with the SF-36 health survey questionnaire. Qual Life Res 1993;2(3):169-80. DOI: 10.1007/BF00435221.
47. Herdman M, Gudex C, Lloyd A, et al. Development and preliminary testing of the new five-level version of EQ-5D (EQ-5D-5L). Qual Life Res 2011;20(10):1727-36. DOI: 10.1007/s11136-011-9903-x.
48. van Hout B, Janssen MF, Feng YS, et al. Interim scoring for the EQ-5D-5L: mapping the EQ-5D-5L to EQ-5D-3L value sets. Value Health 2012;15(5):708-15. DOI: 10.1016/j.jval.2012.02.008.
49. Gandek B, Ware JE, Aaronson NK, et al. Cross-validation of item selection and scoring for the SF-12 Health Survey in nine countries: results from the IQOLA Project. International Quality of Life Assessment. J Clin Epidemiol 1998;51(11):1171-8. DOI: 10.1016/s0895-4356(98)00109-7.
50. Ware J, Jr., Kosinski M, Keller SD. A 12-Item Short-Form Health Survey: construction of scales and preliminary tests of reliability and validity. Med Care 1996;34(3):220-33. DOI: 10.1097/00005650-199603000-00003.
51. Katz JM, Hancock K, Xu X. Serologic assays for influenza surveillance, diagnosis and vaccine evaluation. Expert Rev Anti Infect Ther 2011;9(6):669-83. DOI: 10.1586/eri.11.51.
52. Deng Y, Jing Y, Campbell AE, Gravenstein S. Age-related impaired type 1 T cell responses to influenza: reduced activation ex vivo, decreased expansion in CTL culture in vitro, and blunted response to influenza vaccination in vivo in the elderly. J Immunol 2004;172(6):3437-46. (https://www.ncbi.nlm.nih.gov/pubmed/15004143).
53. Shahid Z, Kleppinger A, Gentleman B, Falsey AR, McElhaney JE. Clinical and immunologic predictors of influenza illness among vaccinated older adults. Vaccine 2010;28(38):6145-51. DOI: 10.1016/j.vaccine.2010.07.036.
54. Kwong JC, Vasa PP, Campitelli MA, et al. Risk of Guillain-Barre syndrome after seasonal influenza vaccination and influenza health-care encounters: a self-controlled study. Lancet Infect Dis 2013;13(9):769-76. DOI: 10.1016/S1473-3099(13)70104-X.
55. McNeil MM, Weintraub ES, Duffy J, et al. Risk of anaphylaxis after vaccination in children and adults. J Allergy Clin Immunol 2016;137(3):868-78. DOI: 10.1016/j.jaci.2015.07.048.

APPENDIX

Appendix A: EQ-5D-5L and VAS
Appendix B: Corticosteroid Dose Charts
