# Supplementary material for: Safety of Simultaneous vs Sequential mRNA COVID-19 and Inactivated Influenza Vaccines: A Randomized Clinical Trial
Source: JAMA Netw Open. 2024 Nov 6;7(11):e2443166. doi: 10.1001/jamanetworkopen.2024.43166 (PMC11541642; doi:10.1001/jamanetworkopen.2024.43166)
Supplement: Supplement 2. — eMethods. Participant Eligibility Criteria and Participant Reactogenicity Assessments eTable 1. Local Injection Site and Systemic Reactions by Severity, Including Moderate or Greater Severity, Following Visit 1 eTable 2. Local Injection Site and Systemic Reactions by Severity, Including Moderate or Greater Severity, Following Visit 2 eTable 3. Description of Serious Adverse Events [file jamanetwopen-e2443166-s002.pdf]

Supplementary Online Content

Walter EB, Schlaudecker EP, Talaat KR, et al. Safety of simultaneous vs sequential mRNA COVID-19 and inactivated influenza vaccines: a randomized clinical trial. *JAMA Netw Open*. 2024;7(11):e2443166. doi:10.1001/jamanetworkopen.2024.43166

eMethods. Participant Eligibility Criteria and Participant Reactogenicity Assessments

eFigure. CONSORT Diagram

eTable 1. Local Injection Site and Systemic Reactions by Severity, Including Moderate or Greater Severity, Following Visit 1

eTable 2. Local Injection Site and Systemic Reactions by Severity, Including Moderate or Greater Severity, Following Visit 2

eTable 3. Description of Serious Adverse Events

This supplementary material has been provided by the authors to give readers additional information about their work.

© 2024 Walter EB et al. *JAMA Network Open*.

eMethods:

Participant Eligibility Criteria

1.1 Subject Inclusion Criteria

Subjects who meet all of the following criteria will be eligible to participate in this interventional study.

1. Persons aged ≥5 years[†] if receiving primary two-dose mRNA COVID-19 vaccine series or persons aged ≥12 years if receiving a booster mRNA COVID-19 vaccine dose according to FDA authorization or approval and ACIP recommendation. Note: receipt of an mRNA COVID-19 vaccine within 8 hours of enrollment is permitted
   * Individuals age 5-11 receiving a booster may be enrolled in the event a booster for individuals age 5-11 is authorized or approved and recommended by the ACIP.
2. English or Spanish literate
3. Intention of receiving influenza vaccine and mRNA COVID-19 vaccine based on CDC guidelines
4. Willing to provide written informed consent
5. Intention of being available for entire study period and complete all relevant study procedures, including follow-up phone calls and clinic visits

1.2 Subject Exclusion Criteria

Subjects who meet any of the following criteria will not be eligible to participate in this study:

1. Currently pregnant, planning to become pregnant within the first three months of the study per participant self-report or likely to be pregnant per screening criteria as defined in Section 5.1 of the study protocol at Visit 1
2. Prior receipt of IIV4 during the respective influenza season in which they are being enrolled
3. < 9 years of age and recommended to receive two doses of IIV4 during the respective influenza season in which they are being enrolled
4. Prior receipt of non-mRNA COVID-19 vaccine
5. Documented COVID-19 infection within 6 weeks prior to enrollment confirmed by either medical history or lab testing
6. History of severe allergic reaction after a previous dose of any influenza vaccine; or to an influenza vaccine component, including egg protein
7. History of severe adverse reaction associated with a vaccine and/or severe allergic reaction (e.g. anaphylaxis) to any component of an mRNA vaccine
8. Receipt of any licensed inactivated vaccine within 2 weeks prior to enrollment in this study, receipt of any licensed live vaccine within 4 weeks prior to enrollment in this study, or receipt of Shingrix (Zoster Vaccine Recombinant, Adjuvanted) or HEPLISAV-B (Hepatitis B Vaccine (Recombinant), Adjuvanted) vaccine within 6 weeks prior to enrollment in this study or planning receipt of any vaccines following enrollment until 6 weeks after receipt of the second dose of mRNA COVID-19 vaccine
9. Has an active neoplastic disease (excluding non-melanoma skin cancer or prostate cancer that is stable in the absence of therapy) or a history of any hematologic malignancy*
   **Participants with a history of malignancy may be included if, after previous treatment by surgical excision, chemotherapy or radiation therapy, the participant has been observed for a period that in the investigator's estimation provides a reasonable assurance of sustained cure*
10. Thrombocytopenia, bleeding disorder, or anticoagulant use contraindicating intramuscular injection (a daily aspirin may be acceptable).
11. Has immunosuppression as a result of an underlying illness or medications, such as antirejection/transplant regimens or immunomodulatory agents. Stable HIV disease is permitted per the following parameters:
    a. Confirmed stable HIV disease defined as document viral load <50 copies/mL and CD4 count >200 within 6 months before enrollment, and on stable antiretroviral therapy for at least 6 months
12. Has known hepatitis B (HBV) or hepatitis C (HBC). Stable HBV or HBC are permitted per the following parameters:
    a. If known HBV: confirmed inactive chronic HBV infection: HBsAg present for ≥6 months and HBeAg negative, anti-HBe positive; serum HBV DNA <2000 IU/mL; persistently normal ALT or AST levels; in those who had liver biopsy, findings that confirm absence of significant necroinflammation

© 2024 Walter EB et al. *JAMA Network Open*.

    b. If known HCV: evidence of sustained virological response for ≥12 weeks after treatment or without evidence of HCV RNA viremia (undetectable HCV RNA)
13. Use of oral, parenteral, or high-dose inhaled glucocorticoids*
    **For definition of high-dose inhaled glucocorticoids, reference Appendix B.*
14. History of Guillain-Barré syndrome
15. Prior enrollment in this study during the 2021-22 flu season
16. Anyone who is already enrolled or plans to enroll in another clinical trial with an investigational product during the study period.*
    **Per protocol, co-enrollment in observational or behavioral intervention studies are permitted at any time. An investigational product may be permitted for therapy of an illness condition that occurs during the study period e.g. COVID-19 illness.*
17. Hearing loss determined by the investigators to prevent successful communication over the phone
18. History of myocarditis or pericarditis
19. History of multisystem inflammatory syndrome in children (MIS-C) or adults (MIS-A).
20. Has injury or other reason why deltoid site on both arms cannot be used for vaccinations.
21. Any condition which, in the opinion of the investigators, may pose a health risk to the subject or interfere with the evaluation of the study objectives.
22. Anyone who is a relative of any research study personnel.
23. Anyone who is an employee of any research study personnel.

1.3 Temporary Delay Criteria (Visit 1, 2 and 3 (for primary COVID-19 vaccine series))

1. History of febrile illness (> 100.0°F or 37.8°C) within the past 72 hours prior to vaccine administration

†During the 2021-2022 season persons 12 years of age and older were considered eligible. During the 2022-2023 season persons 5 years of age and older were considered eligible.

© 2024 Walter EB et al. *JAMA Network Open*.

Participant Reactogenicity Assessments: Those submitting memory aid information electronically received daily electronic reminders and electronic information was reviewed at least twice between Days 2 and 4 and Days 8 and 10 for completeness. Participants were contacted to ascertain missing diary information. Others using a paper memory aid received a single reminder phone call between Days 2 and 4

**Injection-site Reactogenicity Grading**				
**Local Reaction to Injectable Product**	**Mild (Grade 1)**	**Moderate (Grade 2)**	**Severe (Grade 3)**	**Potentially Life Threatening (Grade 4)**
**Pain**	Noticeable but does not interfere with activity	Interferes with activity but did not need a medical visit or absenteeism [i.e. missing work or school]	Significant; prevents daily activity and/or resulted in medical visit and/or absenteeism [i.e. missing work or school]	Requires an emergency room (ER) visit or hospitalization
**Induration/Swelling (≥ 12 years of age)**	2.5 – 5 cm	5.1 – 10 cm	> 10 cm	Requires an emergency room (ER) visit or hospitalization
**Induration/Swelling (< 12 years of age)**	0.5 – 2 cm	2.0 -7.0 cm	> 7 cm	Requires an emergency room (ER) visit or hospitalization
**Erythema/Redness (≥ 12 years of age)**	2.5 – 5 cm	5.1 – 10 cm	> 10 cm	Requires an emergency room (ER) visit or hospitalization
**Erythema/Redness (< 12 years of age)**	0.5 – 2 cm	2.0 -7.0 cm	> 7 cm	Requires an emergency room (ER) visit or hospitalization
**Axillary (underarm) swelling or tenderness ipsilateral to side of injection**	Noticeable but does not interfere with activity	Interferes with activity but did not need a medical visit or absenteeism [i.e. missing work or school]	Significant; prevents daily activity and/or resulted in medical visit and/or absenteeism [i.e. missing work or school]	Requires an emergency room (ER) visit or hospitalization

© 2024 Walter EB et al. *JAMA Network Open*.

Systemic Reactogenicity Grading (FDA modified)				
**Systemic**	**Mild (Grade 1)**	**Moderate (Grade 2)**	**Severe (Grade 3)**	**Potentially Life Threatening (Grade 4)**
**Fever (°C)**<br>**(°F)**	38.0 - 38.4<br>100.4 - 101.1	38.5 - 38.9<br>101.2 - 102.0	39.0 – 40.0<br>102.1-104.0	> 40.0<br>>104.0
**Nausea/vomiting**	Noticeable but does not interfere with activity or 1 – 2 episodes/24 hours	Some interference with activity or > 2 episodes/24 hours	Significant; prevents daily activity and/or resulted in medical visit and/or absenteeism [i.e. missing work or school]	Requires an ER visit or hospitalization
**Diarrhea**	Noticeable but does not interfere with activity or 2 – 3 loose stools/24 hours	Some interference with activity or 4-5 loose stools/24 hours	Significant; prevents daily activity and/or resulted in medical visit and/or absenteeism [i.e. missing work or school] or 6 or more watery stools or > 24 hours	Requires an ER visit or hospitalization
**Headache**	Noticeable but does not interfere with activity	Some interference with activity but did not need a medical visit or absenteeism [i.e. missing work or school]	Significant; prevents daily routine activity and/or resulted in medical visit and/or absenteeism [i.e. missing work or school]	Requires an ER visit or hospitalization
**Fatigue**	Noticeable but does not interfere with activity	Some interference with activity but did not need a medical visit or absenteeism [i.e. missing	Significant; prevents daily routine activity and/or resulted in medical visit and/or absenteeism [i.e. missing	Requires an ER visit or hospitalization

© 2024 Walter EB et al. *JAMA Network Open*.

Systemic Reactogenicity Grading (FDA modified)				
**Systemic**	**Mild (Grade 1)**	**Moderate (Grade 2)**	**Severe (Grade 3)**	**Potentially Life Threatening (Grade 4)**
		work or school]	work or school]	
**Myalgia**	Noticeable but does not interfere with activity	Some interference with activity but did not need a medical visit or absenteeism [i.e. missing work or school]	Significant; prevents daily routine activity and/or resulted in medical visit and/or absenteeism [i.e. missing work or school]	Requires an ER visit or hospitalization
**Arthralgia**	Noticeable but does not interfere with activity	Some interference with activity but did not need a medical visit or absenteeism [i.e. missing work or school]	Significant; prevents daily routine activity and/or resulted in medical visit and/or absenteeism [i.e. missing work or school]	Requires an ER visit or hospitalization
**Chills**	Noticeable but does not interfere with activity	Some interference with activity but did not need a medical visit or absenteeism [i.e. missing work or school]	Significant; prevents daily routine activity and/or resulted in medical visit and/or absenteeism [i.e. missing work or school]	Requires an ER visit or hospitalization

© 2024 Walter EB et al. *JAMA Network Open*.

**Unsolicited Adverse Event, Serious Adverse Event and Adverse Event of Special Interest Grading**			
**Mild (Grade 1)**	**Moderate (Grade 2)**	**Severe (Grade 3)**	**Potentially Life Threatening (Grade 4)**
Noticeable but does not interfere with activity or measurement	Interferes with activity but did not need a medical visit or absenteeism [i.e. missing work or school]	Significant; prevents daily activity and/or resulted in medical visit and/or absenteeism [i.e. missing work or school]	Requires an ER visit or hospitalization

© 2024 Walter EB et al. *JAMA Network Open*.

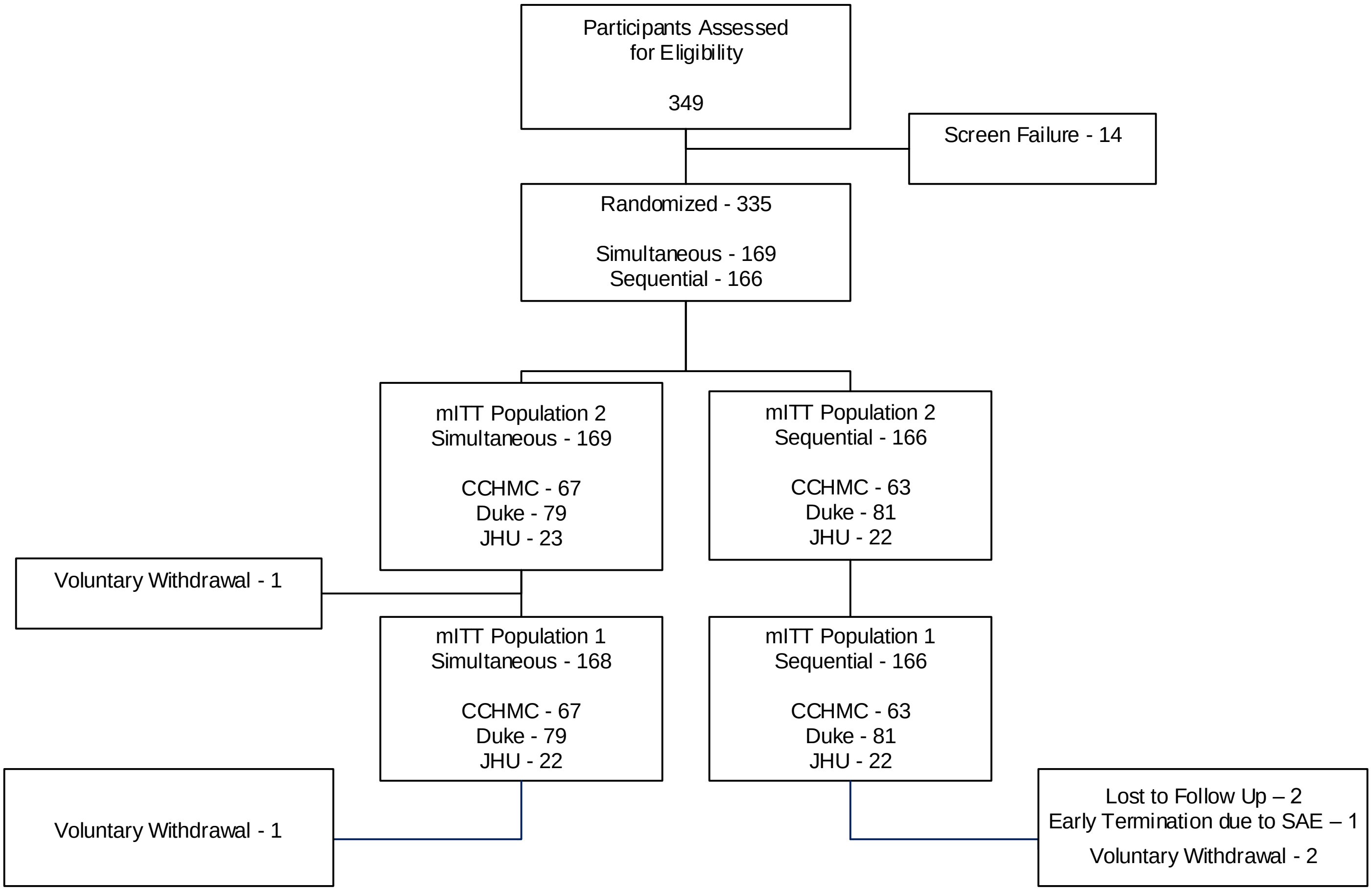

eFigure: Consolidated Standards of Reporting Trials (CONSORT) Diagram

© 2024 Walter EB et al. *JAMA Network Open*.

eTable 1: Local Injection Site and Systemic Reactions by Severity, Including Moderate or Greater Severity, Following Visit 1

		Local Injection Site Reactions Following Visit 1												
		**None**		**Mild**		**Moderate**		**Severe**		**Life Threatening**		**Moderate or Greater**		**Simultaneous minus Sequential[1]**
**Outcome**	**Group**	N	%	**N**	**%**	**N**	**%**	**N**	**%**	**N**	**%**	**N**	**%**	**Percentage Point Difference 95%pt. CI**
Pain: COVID Site	Simultaneous	39	23.2	102	60.7	27	16.1	0	0.0	0	0.0	27	16.1	
	Sequential	27	16.3	97	58.4	40	24.1	2	1.2	0	0.0	42	25.3	-9.23 (-17.87, -0.59)
Pain: Flu/Placebo Site	Simultaneous	65	38.7	86	51.2	16	9.5	1	0.6	0	0.0	17	10.1	
	Sequential	145	87.3	20	12.0	1	0.6	0	0.0	0	0.0	1	0.6	9.52 (4.81, 14.23)
Swelling: COVID Site	Simultaneous	164	97.6	4	2.4	0	0.0	0	0.0	0	0.0	0	0.0	
	Sequential	156	94.0	4	2.4	5	3.0	1	0.6	0	0.0	6	3.6	-3.61 (-6.45, -0.78)
Swelling: Flu/Placebo Site	Simultaneous	164	97.6	2	1.2	2	1.2	0	0.0	0	0.0	2	1.2	
	Sequential	164	98.8	1	0.6	1	0.6	0	0.0	0	0.0	1	0.6	0.59 (-1.43, 2.61)
Redness: COVID Site	Simultaneous	164	97.6	4	2.4	0	0.0	0	0.0	0	0.0	0	0.0	
	Sequential	157	94.6	6	3.6	2	1.2	1	0.6	0	0.0	3	1.8	-1.81 (-3.83, 0.22)
Redness: Flu/Placebo Site	Simultaneous	165	98.2	1	0.6	1	0.6	1	0.6	0	0.0	2	1.2	
	Sequential	165	99.4	0	0.0	1	0.6	0	0.0	0	0.0	1	0.6	0.59 (-1.43, 2.61)
Axillary Swell/Tenderness: COVID Site	Simultaneous	120	71.4	40	23.8	8	4.8	0	0.0	0	0.0	8	4.8	
	Sequential	111	66.9	43	25.9	11	6.6	1	0.6	0	0.0	12	7.2	-2.47 (-7.56, 2.62)
Axillary Swell/Tenderness: Flu/Placebo Site	Simultaneous	135	80.4	29	17.3	4	2.4	0	0.0	0	0.0	4	2.4	
	Sequential	158	95.2	5	3.0	3	1.8	0	0.0	0	0.0	3	1.8	0.57 (-2.50, 3.64)

[1] Moderate or higher reactions only

© 2024 Walter EB et al. *JAMA Network Open*.

		Systemic Reactions Following Visit 1												
		None		Mild		Moderate		Severe		Life Threatening		Moderate or Greater		Simultaneous minus Sequential[1]
**Outcome**	**Group**	**N**	**%**	**N**	**%**	**N**	**%**	**N**	**%**	**N**	**%**	**N**	**%**	**Percentage Point Difference 95%pt. CI**
Fever	Simultaneous	159	94.6	4	2.4	3	1.8	2	1.2	0	0.0	5	3.0	
	Sequential	155	93.4	6	3.6	2	1.2	3	1.8	0	0.0	5	3.0	-0.04 (-3.69, 3.62)
Chills	Simultaneous	121	72.0	29	17.3	15	8.9	3	1.8	0	0.0	18	10.7	
	Sequential	116	69.9	26	15.7	19	11.4	5	3.0	0	0.0	24	14.5	-3.74 (-10.85, 3.36)
Fatigue	Simultaneous	73	43.5	50	29.8	41	24.4	4	2.4	0	0.0	45	26.8	
	Sequential	61	36.7	61	36.7	34	20.5	10	6.0	0	0.0	44	26.5	0.28 (-9.20, 9.76)
Myalgia	Simultaneous	71	42.3	62	36.9	31	18.5	4	2.4	0	0.0	35	20.8	
	Sequential	65	39.2	60	36.1	33	19.9	8	4.8	0	0.0	41	24.7	-3.87 (-12.85, 5.12)
Headache	Simultaneous	90	53.6	53	31.5	21	12.5	4	2.4	0	0.0	25	14.9	
	Sequential	80	48.2	58	34.9	25	15.1	3	1.8	0	0.0	28	16.9	-1.99 (-9.82, 5.85)
Arthralgia	Simultaneous	141	83.9	14	8.3	12	7.1	1	0.6	0	0.0	13	7.7	
	Sequential	129	77.7	21	12.7	11	6.6	5	3.0	0	0.0	16	9.6	-1.90 (-7.94, 4.14)
Nausea/Vomiting	Simultaneous	145	86.3	15	8.9	7	4.2	1	0.6	0	0.0	8	4.8	
	Sequential	142	85.5	19	11.4	3	1.8	2	1.2	0	0.0	5	3.0	1.75 (-2.39, 5.89)
Diarrhea	Simultaneous	151	89.9	11	6.5	6	3.6	0	0.0	0	0.0	6	3.6	
	Sequential	146	88.0	17	10.2	2	1.2	1	0.6	0	0.0	3	1.8	1.76 (-1.70, 5.23)

[1] Moderate or higher reactions only

© 2024 Walter EB et al. *JAMA Network Open*.

eTable 2: Local Injection Site and Systemic Reactions By Severity Including Moderate or Greater Severity Following Visit 2

		Local Injection Site Reactions Following Visit 2												
		None		Mild		Moderate		Severe		Life Threatening		Moderate or Greater		Simultaneous minus Sequential[1]
**Outcome**	**Group**	**N**	**%**	**N**	**%**	**N**	**%**	**N**	**%**	**N**	**%**	**N**	**%**	**Percentage Point Difference 95%pt. CI**
Pain: Flu/Placebo Site	Simultaneous	161	95.8	6	3.6	0	0.0	1	0.6	0	0.0	1	0.6	
	Sequential	82	49.4	75	45.2	8	4.8	1	0.6	0	0.0	9	5.4	-4.83 (-8.46, -1.19)
Swelling: Flu/Placebo Site	Simultaneous	168	100.0	0	0.0	0	0.0	0	0.0	0	0.0	0	0.0	
	Sequential	160	96.4	4	2.4	2	1.2	0	0.0	0	0.0	2	1.2	-1.20 (-2.86, 0.45)
Redness: Flu/Placebo Site	Simultaneous	168	100.0	0	0.0	0	0.0	0	0.0	0	0.0	0	0.0	
	Sequential	164	98.8	2	1.2	0	0.0	0	0.0	0	0.0	0	0.0	0.00 (0.00, 0.00)
Axillary Swell/Tenderness: Flu/Placebo Site	Simultaneous	165	98.2	3	1.8	0	0.0	0	0	0	0.0	0	0.0	
	Sequential	141	84.9	21	12.7	3	1.8	1	0.6	0	0.0	4	2.4	-2.41 (-4.74, -0.08)

[1] Moderate or higher reactions only

© 2024 Walter EB et al. *JAMA Network Open*.

		Systemic Reactions Following Visit 2												
		None		Mild		Moderate		Severe		Life Threatening		Moderate or Greater		Simultaneous minus Sequential[1]
Outcome	Group	N	%	N	%	N	%	N	%	N	%	N	%	Percentage Point Difference 95%pt. CI
Fever	Simultaneous	165	98.2	2	1.2	1	0.6	0	0.0	0	0.0	1	0.6	
	Sequential	162	97.6	3	1.8	1	0.6	0	0.0	0	0.0	1	0.6	-0.01 (-1.66, 1.65)
Chills	Simultaneous	167	99.4	0	0.0	1	0.6	0	0.0	0	0.0	1	0.6	
	Sequential	157	94.6	7	4.2	2	1.2	0	0.0	0	0.0	2	1.2	-0.61 (-2.64, 1.42)
Fatigue	Simultaneous	150	89.3	10	6.0	4	2.4	4	2.4	0	0.0	8	4.8	
	Sequential	121	72.9	34	20.5	10	6.0	1	0.6	0	0.0	11	6.6	-1.86 (-6.83, 3.10)
Myalgia	Simultaneous	159	94.6	5	3.0	4	2.4	0	0.0	0	0.0	4	2.4	
	Sequential	124	74.7	34	20.5	7	4.2	1	0.6	0	0.0	8	4.8	-2.44 (-6.43, 1.55)
Headache	Simultaneous	141	83.9	17	10.1	9	5.4	1	0.6	0	0.0	10	6.0	
	Sequential	129	77.7	26	15.7	11	6.6	0	0.0	0	0.0	11	6.6	-0.67 (-5.88, 4.53)
Arthralgia	Simultaneous	161	95.8	5	3.0	2	1.2	0	0.0	0	0.0	2	1.2	
	Sequential	156	94.0	7	4.2	3	1.8	0	0.0	0	0.0	3	1.8	-0.62 (-3.22, 1.99)
Nausea/Vomiting	Simultaneous	163	97.0	4	2.4	1	0.6	0	0.0	0	0.0	1	0.6	
	Sequential	160	96.4	4	2.4	2	1.2	0	0.0	0	0.0	2	1.2	-0.61 (-2.64, 1.42)
Diarrhea	Simultaneous	163	97.0	5	3.0	0	0.0	0	0.0	0	0.0	0	0.0	
	Sequential	152	91.6	14	8.4	0	0.0	0	0.0	0	0.0	0	0.0	0.00 (0.00, 0.00)

[1] Moderate or higher reactions only

© 2024 Walter EB et al. *JAMA Network Open*.

eTable 3: Descriptions of Serious Adverse Events

Group	Vaccine	Onset since enrollment vaccination	Sex	Age Group years	Category	Relatedness	Description
Sequential	Pfizer-BioNTech Bivalent	14 days*<br>*Occurred same day following Visit 2.	Female	50-64	Hospitalization	Unlikely related	Small bowel obstruction with incarcerated ventral hernia. Past medical history included abdominal surgeries and cancer
Simultaneous	Pfizer-BioNTech Monovalent	19 weeks	Female	18-49	Other medically important event	Not related	Spontaneous abortion occurring at 16 weeks gestation. Past medical history included COVID-19 illness (mild) 9 weeks after visit 1 (considered an AESI); (Note: Participant did not report being pregnant or intention of becoming pregnant at enrollment)

© 2024 Walter EB et al. *JAMA Network Open*.
