# Supplementary material for: Safety of Simultaneous vs Sequential mRNA COVID-19 and Inactivated Influenza Vaccines: A Randomized Clinical Trial
Source: JAMA Netw Open. 2024 Nov 6;7(11):e2443166. doi: 10.1001/jamanetworkopen.2024.43166 (PMC11541642; doi:10.1001/jamanetworkopen.2024.43166)
Supplement: Supplement 3. — Data Sharing Statement [file jamanetwopen-e2443166-s003.pdf]

Data Sharing Statement

Walter. Safety of Simultaneous vs Sequential mRNA COVID-19 and Inactivated Influenza Vaccines. *JAMA Netw Open*. Published November 06, 2024.
doi:10.1001/jamanetworkopen.2024.43166

Data
Additional Information: Clinicaltrials.gov - Simultaneous mRNA COVID-19 and IIV4 Vaccination StudyURL: https://clinicaltrials.gov/study/NCT05028361?term=NCT05028361%20&rank=1 Trial registration number: NCT05028361
Data available: No

Additional Information
Explanation for why data not available: Informed Consent for this trial did not allow for data sharing
